# *Pseudomonas aeruginosa* induces the generation of pathogenic IFNγ^+^Th17 cells that promote lung damage and chronic inflammation in cystic fibrosis

**DOI:** 10.1038/s41564-026-02469-2

**Published:** 2026-09-24

**Authors:** Irene Dusetti, Gianmarco Conte, Matteo Chiara, Simone Puccio, Daniela Guidone, Eugenia Ricciardelli, Javier Cibella, Mirko Ronzio, Diletta Dolfini, Elio Rossi, Paolo Landini, Miriam Ascagni, Riccardo Orlandi, Francesco Damarco, Alessandro Palleschi, Andrea Gramegna, Francesco Blasi, Enrico Lugli, Luis J. Galietta, Søren Molin, Helle K. Johansen, Clelia Peano, Moira Paroni

**Affiliations:** 1 Department of Biosciences, Università degli Studi di Milano, Milan, Italy; 2 Laboratory of Translational Immunology, IRCCS Humanitas Research Hospital, Milan, Italy; 3 Institute of Genetics and Biomedical Research, UoS of Milan, National Research Council, Milan, Italy; 4 Telethon Institute of Genetics and Medicine (TIGEM), Pozzuoli, Italy; 5 Human Technopole, Milan, Italy; 6 Unitech NOLIMITS, Università degli studi di Milano, Milan, Italy; 7 Thoracic Surgery and Lung Transplant Unit, Fondazione IRCCS Ca’ Granda Ospedale Maggiore Policlinico di Milano, Milan, Italy; 8 Department of Pathophysiology and Transplantation, Università degli Studi di Milano, Milan, Italy; 9 Respiratory and Cystic Fibrosis Unit, Fondazione IRCCS Cà Granda Ospedale Maggiore Policlinico di Milano, Milan, Italy; 10 Department of Translational Medical Science, Università degli Studi di Napoli ‘Federico II’, Napoli, Italy; 11 The Novo Nordisk Foundation Center for Biosustainability, Technical University of Denmark, Kgs Lyngby, Denmark; 12 Department of Clinical Microbiology, Rigshospitalet, Copenhagen, Denmark; 13 Department of Clinical Medicine, Faculty of Health and Medical Sciences, University of Copenhagen, Copenhagen, Denmark

**Keywords:** Cellular microbiology, Mucosal immunology

## Abstract

Cystic fibrosis (CF) involves cycles of bacterial infection and sustained inflammation. IFNγ^+^Th17 cells are implicated in chronic inflammation, yet their role in CF remains undefined. Here we identify two pathogenic Th17 subsets, Th1/17^+^ and pro-inflammatory Th17 (pTh17) cells, selectively enriched in CF lungs colonized by *Pseudomonas aeruginosa*. These pathogenic subsets derive from protective conventional Th17 (cTh17) precursors and, following activation, directly disrupt pulmonary epithelial integrity and trigger exaggerated inflammatory responses. Transcriptomic and T cell receptor profiling reveal in situ reprogramming and clonal selection of Th1/17^+^ and pTh17 cells in CF lungs. Mechanistically, clinical *P. aeruginosa* strains persist within dendritic cells and induce a polarizing IL-1β/IL-23 cytokine axis that promotes IFNγ^+^Th17 differentiation over classical Th1 lineage commitment. *P. aeruginosa* early isolates, lacking chronic adaptation signatures, still potently reprogramme cTh17 cells, inducing a transcriptional programme that mirrors CF lung-resident Th1/17^+^ and pTh17 profiles. These findings show that *P. aeruginosa* subverts mucosal immunity and establishes a self-sustaining immunopathological loop, offering potential targets for CF precision therapies.

## Abstract (web summary)

*Pseudomonas aeruginosa* drives harmful immune responses in cystic fibrosis by reprogramming protective Th17 cells into pathogenic populations that damage lung tissue and sustain chronic inflammation.

## Main

Cystic fibrosis (CF) is a life-limiting autosomal recessive disorder characterized by a cycle of infection and sustained inflammation, which synergistically drive progressive lung function decline and, ultimately, respiratory failure^1^. Mutations in the gene encoding the CF transmembrane conductance regulator (CFTR) protein give rise to pulmonary epithelial abnormalities promoting an aberrant activation of the mucosal immune system^2^ that, while failing to eradicate bacterial infections, primarily driven by *Pseudomonas aeruginosa*^3^, further contribute to lung damage.

In the CF lung, this dysregulated immune response is marked by excessive neutrophilic infiltration^4^, overproduction of pro-inflammatory mediators^5^ and progressive epithelial barrier damage^6^. Consistently, current therapeutic strategies mainly include antimicrobial therapies^7^, which remain a cornerstone in CF management despite *P. aeruginosa* often being antibiotic resistant^8^, anti-inflammatory drugs^9^ and CFTR modulators^10^. Moreover, despite their effectiveness in restoring chloride channel function, CFTR modulators still fail to prevent bacterial infections^11^ and their efficacy in reducing CF airway inflammation is still debated^12^. Furthermore, current anti-inflammatory treatments are limited by their broad immunosuppressive effects^13^, underlining the critical need for precision immunomodulatory strategies that selectively target pathogenic pathways while preserving protective mucosal immune responses.

Traditionally, CF airway inflammation was attributed to an imbalanced activation of protective Th1 cells, characterized by IFNγ secretion, and an atypical Th2 response with enhanced production of IL-4 and IL-5, which inversely correlates with lung function in chronically *P. aeruginosa*-infected patients^14^. The discovery of the Th17 lineage fundamentally reshaped this paradigm.

Elevated IL-17 levels in CF airways initially led to the assumption that Th17 cells were intrinsically pathogenic, owing to their role in sustaining neutrophil recruitment and exacerbating tissue-destructive inflammation^15–17^. However, subsequent studies have redefined this view, revealing functional heterogeneity within Th17 cells^18,19^. Protective conventional Th17 (cTh17) cells, co-producing IL-17, IL-10 and IL-22, promote epithelial integrity and mucosal homeostasis^20,21^. By contrast, a distinct pro-inflammatory population, defined Th1/17 cells for their IFNγ/IL-17 co-expression but lacking IL-10, has been implicated in several chronic inflammatory diseases^22–25^.

IFNγ^+^Th17 cell differentiation is driven by exposure to specific antigens in the presence of elevated IL-1β and IL-23 levels^25–27^. These cytokines are highly enriched in CF airways chronically infected with *P. aeruginosa*, and their levels positively correlate with disease severity and lung function decline^28–30^. Nevertheless, the specific involvement of IFNγ^+^Th17 cells in promoting and sustaining CF airway immune dysregulation, and in directly driving epithelial damage and lung function decline, remains undefined.

Here we provided direct evidence linking IFNγ^+^Th17 subset activation to lung tissue damage, providing mechanistic insight into pulmonary pathogenic T cell generation. We showed that, contrary to protective cTh17 cells, pathogenic IFNγ^+^Th17 cell subsets—particularly CCR5^+^Th1/17 (Th1/17^+^) and pro-inflammatory CCR5^+^Th17 (pTh17) cells—are selectively expanded in CF lungs, directly contribute to epithelial barrier disruption and show distinct transcriptional signatures and T cell receptor (TCR) clonal expansions unique to CF. Furthermore, we proved that the cytokine milieu elicited by *P. aeruginosa* strains drives the recruitment of cTh17 precursors and their transdifferentiation into pathogenic Th1/17^+^ and pTh17 cells with distinct transcriptional programmes.

Collectively, our findings identify Th1/17^+^ and pTh17 subsets, and the immunological processes driving their generation, as key components of CF lung immunopathology and suggest that these pathways may represent potential targets for more selective immunomodulatory strategies.

## Results

### IFNγ^+^Th17 cells are expanded in CF lungs, associated with chronic *P. aeruginosa* infection, and directly promote pulmonary epithelial damage

IFNγ^+^Th17 cells have a pathogenic role in several chronic inflammatory diseases^22–25^, yet their contribution to CF mucosal immune pathology remains unclear.

**Fig. 1:**
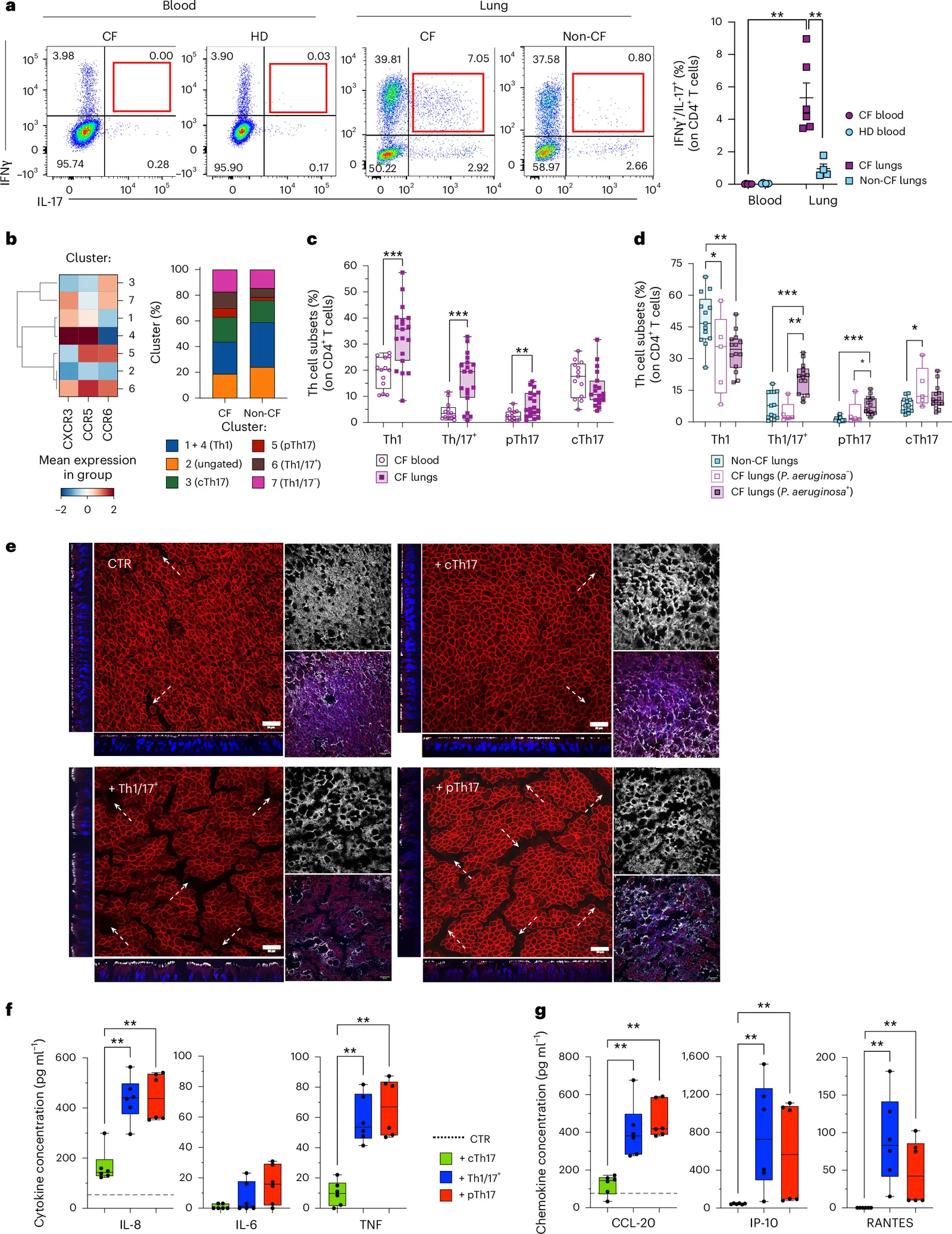
Pathogenic IFNγ^+^Th17 subsets are enriched in CF lungs and directly promote epithelial cell damage and inflammatory response. **a**, Percentage of IFNγ^+^/IL-17^+^ co-expression on ex vivo polyclonally stimulated CD4^+^ T cells isolated from blood of patients with CF (*n* = 6) and HDs (*n* = 6), or from lung tissues of patients with CF (*n* = 6) and without CF (*n* = 4), were measured by intracellular staining. One representative flow cytometry dot plot along with the corresponding graphical summary is shown. **b**, Heat map and corresponding stacked bar plots showing clustering profiles of CD4^+^IL-7R^+^CD25^low^ T cells from lung tissue of patients with CF (*n* = 9) and without CF (*n* = 9). Clusters were identified based on the combinatorial expression of the indicated chemokine receptors using the PhenoGraph algorithm implemented in the CRUSTY software^63^ and were manually annotated according to surface marker expression as detailed in Methods. Each row represents an individual cluster and each column a chemokine receptor, with colours representing scaled (*z*-scored) mean expression values for each receptor across clusters. Stacked bar plots on the right depict the mean percentage of each cluster within CF and non-CF lung samples, illustrating disease-associated differences in cluster distribution. **c**, Mean frequencies of indicated T cell subsets in lung tissues (*n* = 19) and blood (*n* = 13) of patients with CF. **d**, Percentage of indicated T cell subsets in CF lungs stratified according to chronic *P. aeruginosa* infection (*P. aeruginosa*^*+*^, *n* = 14; *P. aeruginosa⁻*, *n* = 5) and lungs of patients without CF (*n* = 13). **e**, Representative immunofluorescence images of ZO-1 (tight junction, red), acetylated tubulin (cilia, white) and nuclei (DAPI, blue) in primary ALI-cultured human bronchial epithelial cells co-cultured with the indicated T cell subsets isolated from CF blood (*n* = 6) or left untreated (CTR). The main picture shows the apical view, with the side panels *YZ* (left) and *XZ* (bottom) showing sagittal sections of the Transwell membrane. Scale bars, 20 μm. White arrows indicate areas of epithelial disruption characterized by discontinuity of the epithelial layer. Data are representative of six independent experiments; two additional replicates are shown in Extended Data Fig. 3f–g). **f**,**g**, Cytokine (**f**) and chemokine (**g**) concentrations in the basolateral medium of ALI-culture human primary bronchial epithelial cells co-cultured with the indicated ex vivo polyclonally stimulated T cell subsets (six independent experiments) or untreated (CTR, four independent experiments). Data (**c**, **d**, **f** and **g**) are presented as box-and-whisker plots showing the median (centre line), minimum-to-maximum range (whiskers) and individual data points. Statistical significance was calculated using unpaired *t*-test with Welch’s correction (**a** and **c**), one-way ANOVA (**d**) or Mann–Whitney test (**f** and **g**) and reported as *P < 0.05, **P < 0.01 and ***P < 0.001. Data distribution in bar graphs are presented as median ± interquartile range (IQR). Source data

**Extended Data Fig. 1:**
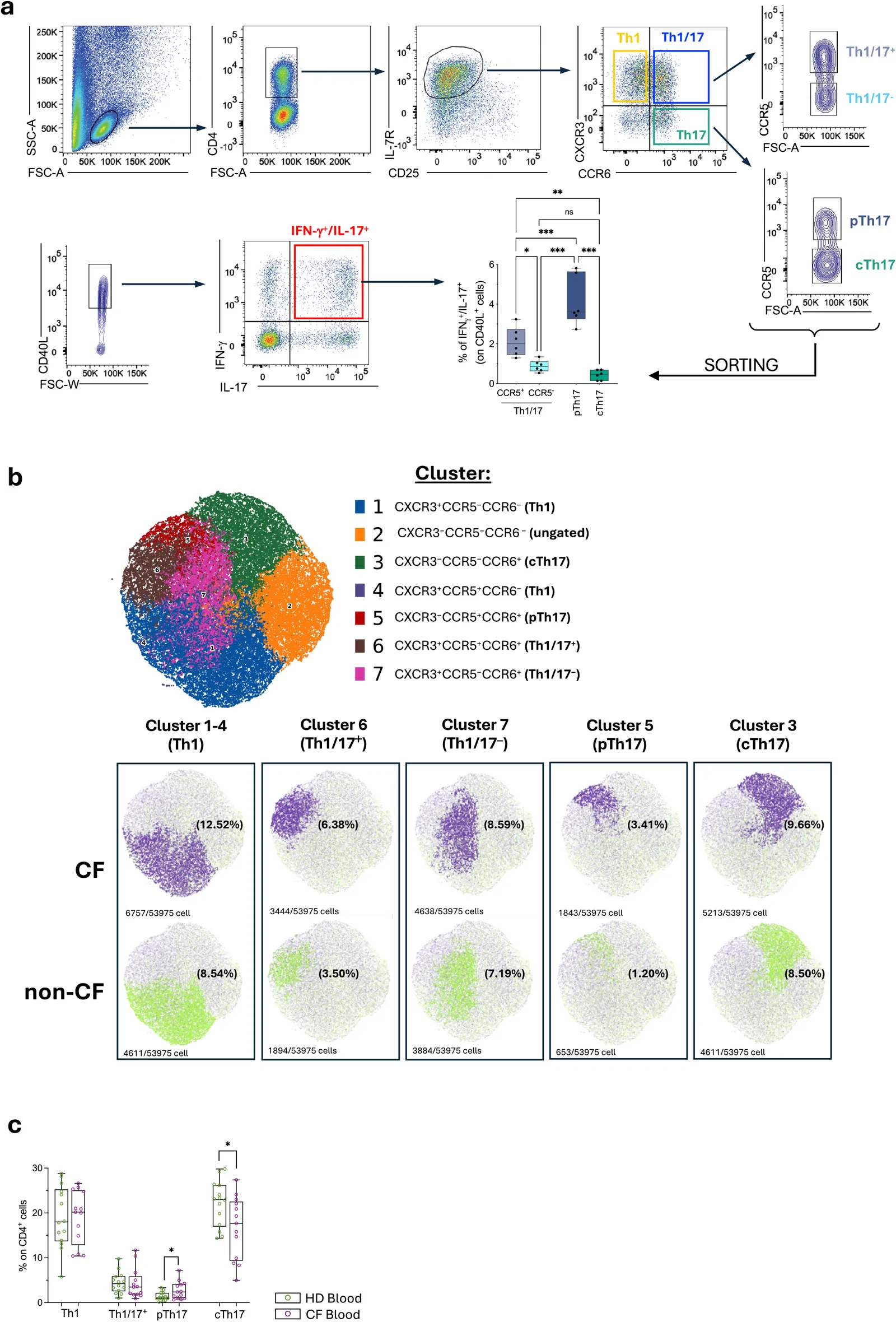
Phenotypic characterization and tissue distribution of IFNγ-producing Th17 subsets. **a)** Gating strategy to sort-purify conventional Th17 (cTh17), pro-inflammatory Th17 (pTh17), and Th1/17 cell subsets, according to the expression of indicated surface markers and chemokine receptors. The co-expression of IFNγ and IL-17A were evaluated for each sorted subset and reported in the graphical summary on the right presented as box-and-whisker plots showing the median (center line), minimum-to-maximum range (whiskers), and individual data points of 6 independent experiments. **b)** UMAP projection of concatenated CD4^+^IL-7R^+^CD25^low^ T cells from lung samples of CF (n = 9) and non-CF (n = 9) patients, coloured by PhenoGraph-defined clusters. A total of 576567 cells were analysed. Dimensionality reduction and clustering were performed using the CRUSTY workflow^63^ applied to flow cytometry data. Clusters are annotated according to lineage-defining chemokine receptor signatures — CXCR3 (Th1), CCR6 (Th17), and CCR5 (mucosal migration) — as detailed in the Methods. Each point represents a single cell, and colours denote distinct clusters corresponding to the T cell subsets reported on the right. The panel below displays the spatial distribution of clusters together with the relative frequency of each T cell subset across CF and non-CF lung samples. **c**) Mean frequencies of Th1, Th1/17^+^, pTh17 and cTh17 subsets in peripheral blood of HD (n = 14) and CF patients (n = 13). Data are presented as box-and-whisker plots showing the median (center line), minimum-to-maximum range (whiskers), and individual data points. Statistical significance was calculated using Unpaired t test with Welch’s correction and reported as *p < 0.05, ** p < 0.01, *** p < 0.001. Source data

**Extended Data Fig. 2:**
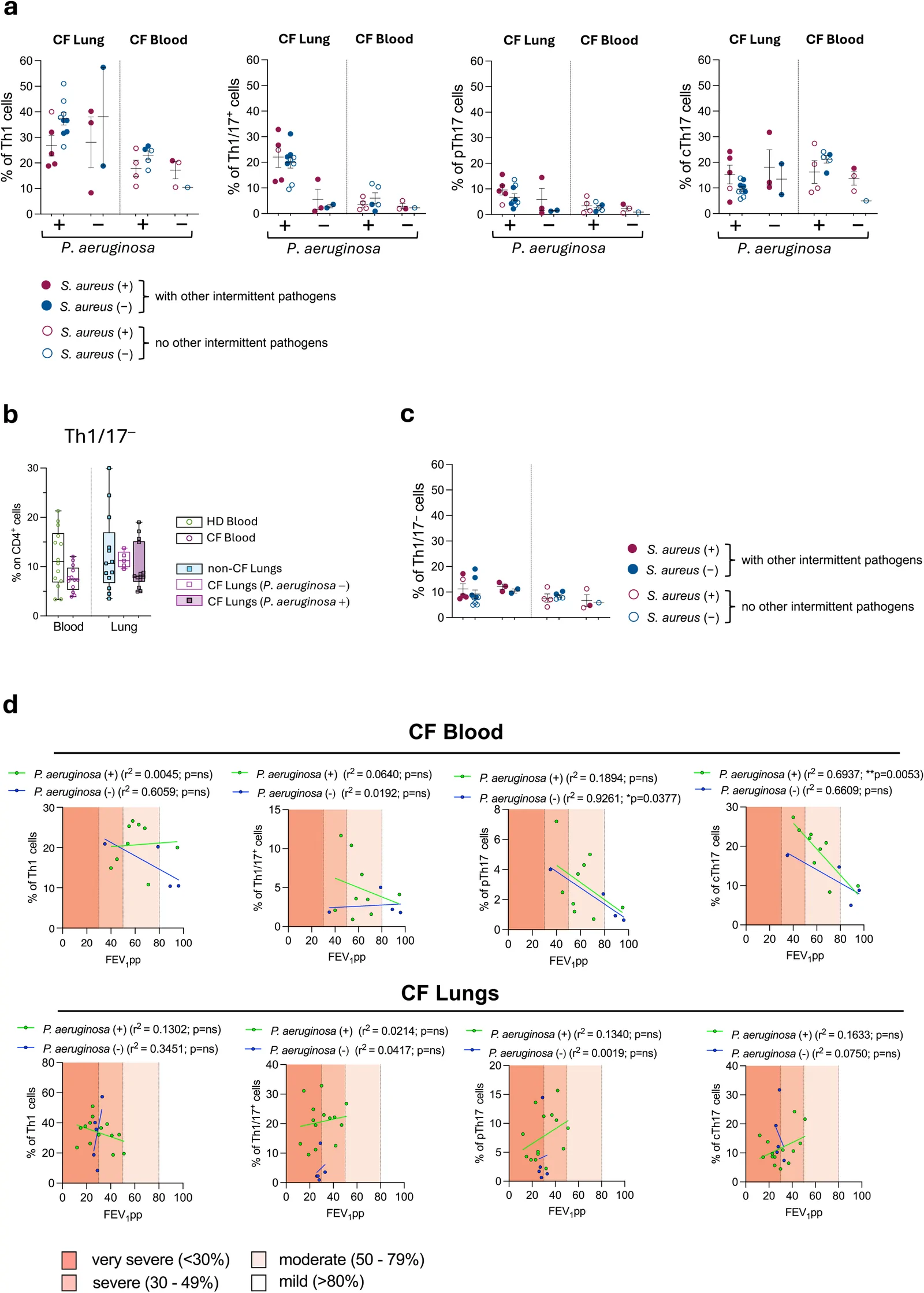
Stratification of peripheral and lung-resident CD4^+^ T cell subsets in pwCF according to pulmonary infection and disease severity. **a)** Frequencies of the indicated T-cell subsets in lungs (n = 19) and blood (n = 13) of CF patients stratified according to chronic *P. aeruginosa* and/or *S. aureus* lung infection. Within each chronic lung infection group the concomitant presence (full circles) or absence (empty circles) of other intermittent pathogens is shown. Each dot represents an individual CF patient. Data distributions are represented as mean ± SEM. **b)** Mean percentages of Th1/17 CCR5⁻ (Th1/17⁻) cells in peripheral blood of HD (n = 14) or CF (n = 13), non-CF lungs (n = 13), or in CF lungs stratified according to the presence (*P. aeruginosa*^+^, n = 14) or absence (*P. aeruginosa*⁻, n = 5) of chronic *P. aeruginosa* lung infection. Data are presented as box-and-whisker plots showing the median (center line), minimum-to-maximum range (whiskers), and individual data points. **c)** Frequencies of Th1/17 CCR5⁻ (Th1/17⁻) cells in lungs (n = 19) and blood (n = 13) of CF patients stratified according to chronic *P. aeruginosa* and/or *S. aureus* lung infection. Within each chronic lung infection group the concomitant presence (full circles) or absence (empty circles) of other intermittent pathogens is shown. **d**) Correlation analysis between FEV_1_pp and the percentage of the indicated T cell subsets in CF blood (upper panels, n = 13) and CF lung tissues (lower panels, n = 19). Background colour bands indicate ranges of FEV_1_pp corresponding to distinct levels of lung disease severity in CF, from mild to very severe, as defined in clinical practice. Positive or negative correlations between variables were evaluated using Pearson correlation analysis. R^2^ together with p values are indicated within each graph. Source data

Flow cytometric profiling revealed a significant enrichment of IFNγ^+^/IL-17^+^ CD4^+^ T cells in CF lungs compared with blood and non-CF lungs (Fig. 1a). As several human IFNγ^+^/IL-17^+^ co-producing CD4^+^ T cells were identified based on surface marker expression^25,31^, we focused on T cell subsets expressing the highest IFNγ^+^/IL-17^+^ levels, namely, CCR5^+^Th1/17 (Th1/17^+^) and pro-inflammatory CCR5^+^Th17 (pTh17) cells (Extended Data Fig. 1a). Unsupervised uniform manifold approximation and projection clustering based on CXCR3, CCR6 and CCR5 expression confirmed a marked increase of lung-resident Th1/17^+^ and pTh17 cells in CF lungs compared with non-CF lungs (Fig. 1b and Extended Data Fig. 1b). Manual gating analysis corroborated a significant enrichment of both lung-resident Th1/17^+^ and pTh17 cells compared with CF blood (Fig. 1c). By contrast, cTh17 cells were slightly reduced in CF lungs compared with CF blood, with a further significant reduction in CF blood compared with healthy donors (HDs) (Fig. 1c and Extended Data Fig. 1c). Stratification of patient cohorts according to key clinical variables (Supplementary Tables 1–4) revealed no correlation between T cell subsets and age, whereas forced expiratory volume in 1 second percent predicted (FEV_1_pp) negatively correlated exclusively with cTh17 and pTh17 cell frequencies in CF blood (Supplementary Fig. 1a,b). Notably, pulmonary Th1/17^+^ and pTh17 cells were significantly enriched exclusively in patients with CF chronically infected with *P. aeruginosa*, irrespective of the concomitant presence of *Staphylococcus aureus* or other intermittent pathogens (Fig. 1d and Extended Data Fig. 2a). Th1 cells predominated in non-CF lungs, while lung-resident cTh17 were increased in *P. aeruginosa*-negative patients with CF (Fig. 1d). By contrast, the frequency of Th1/17^−^ cells remained unchanged across all compartments and was not influenced by microbial status (Extended Data Fig. 2b,c).

Stratification by FEV_1_pp revealed increased peripheral pTh17 cells in patients with CF and more severe disease and higher circulating cTh17 cells exclusively in chronically *P. aeruginosa*-infected patients with CF, whereas no correlation between T cell subsets and FEV_1_pp was detected in CF lungs, probably reflecting the end-stage disease status of the lung cohort (Extended Data Fig. 2d).

**Extended Data Fig. 3:**
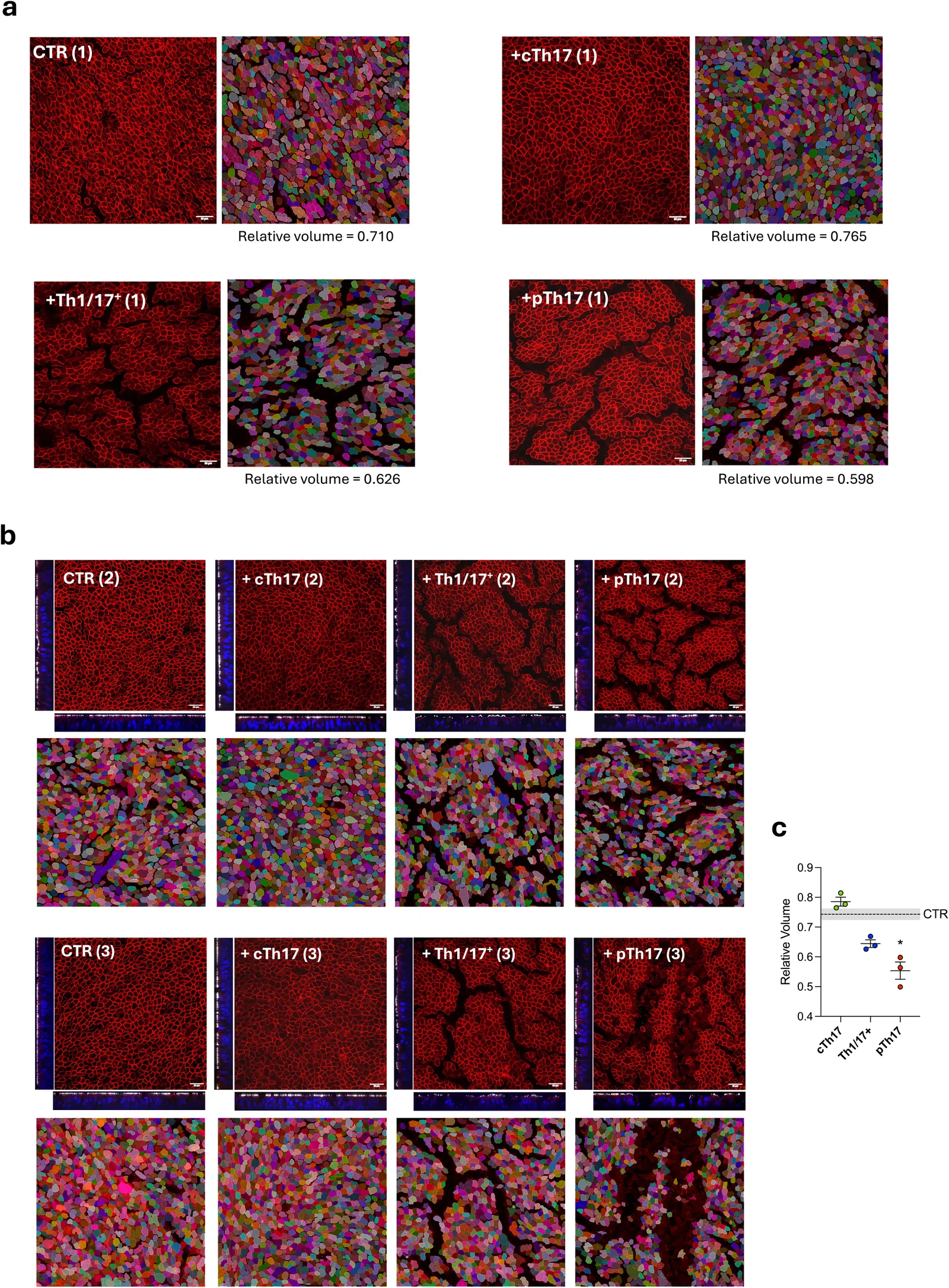
AI-assisted quantitative analysis reveals selective IFNγ^+^Th17-driven disruption of epithelial integrity in ALI-culture human airway epithelial cells. **a)** Quantitative image analysis of ZO-1 staining performed on maximum intensity projections of confocal image stacks from Fig. 1e using AI-assisted image analysis of Arivis Pro v.4.4 (Carl Zeiss Microscopy, GmbH). Segmentation masks were generated to detect ZO-1 signal-defined structures within each field. The total volume was calculated as the sum of pixels within each segmented object, and the relative volume was obtained as the ratio of segmented image size to the total image size. These measurements provide a quantitative estimate of the spatial representation of ZO-1 signal and epithelial junction continuity. Regions lacking ZO-1 signal (black areas in the segmentation output) correspond to junctional discontinuities and epithelial barrier disruption. Scale bar, 20 μm. **b)** Additional representative immunofluorescence images of primary ALI-culture human bronchial epithelial cells co-cultured basolaterally for 24 h with the indicated ex vivo-activated Th17 cell subsets from CF patients or left untreated (CTR). Confocal images show ZO-1 (red), acetylated tubulin (cilia, white) and nuclei (DAPI, blue). The main panels show a top view, with orthogonal projections (YZ, right; XZ, bottom) providing sagittal views of the epithelial layer across the Transwell membrane. Corresponding AI-derived segmentation maps are shown below each condition, where ZO-1-negative areas (black) highlight regions of junctional disruption and epithelial damage. Scale bar, 20 μm. **c)** Quantification of the relative volume of ZO-1 signal-defined structures normalized to the total field, as obtained by AI-assisted analysis. Reduced relative volume reflects loss of junctional continuity and decreased epithelial barrier integrity, providing a quantitative measure of epithelial damage across experimental conditions. Data from 3 independent experiments are represented as mean ± SEM. Statistical analysis was performed using one-way ANOVA and reported as * *p* < 0.05. Source data

We next investigated whether the enrichment of Th1/17^+^ and pTh17 cells merely reflected phenotypic skewing or directly contributed to airway epithelium dysfunction using a primary bronchial epithelial air–liquid interface (ALI) model. Immunofluorescence analysis showed that only activated Th1/17^+^ and pTh17 subsets markedly compromised bronchial epithelial integrity, as indicated by extensive damaged areas with a discontinuous staining pattern (Fig. 1e). Quantitative artificial intelligence (AI)-assisted image analysis of zonula occludens-1 (ZO-1) staining confirmed a significant increase in junctional discontinuity and epithelial damage selectively in cultures exposed to Th1/17^+^ and pTh17 cells (Extended Data Fig. 3a–c). Furthermore, Th1/17^+^ and pTh17 cells elicited a significantly higher pro-inflammatory response from epithelial cells compared with the basal response induced by cTh17 cells (Fig. 1f,g). Together, these findings suggest that Th1/17^+^ and pTh17 cells may act as pathogenic effectors in CF lungs, where their expansion is associated with *P. aeruginosa* airway infection, and their activation directly compromises epithelial barrier integrity, thereby potentially contributing to CF lung function decline.

### Pathogenic IFNγ^+^Th17 cells show distinct transcriptomic and TCR profiles in CF lungs

To define the molecular and antigenic features of Th1/17^+^ and pTh17 cells in CF, we compared transcriptomic and TCR repertoire profiles of ex vivo-sorted peripheral and pulmonary T cell subsets from *P. aeruginosa*-positive patients with CF with those from non-CF lungs.

**Fig. 2:**
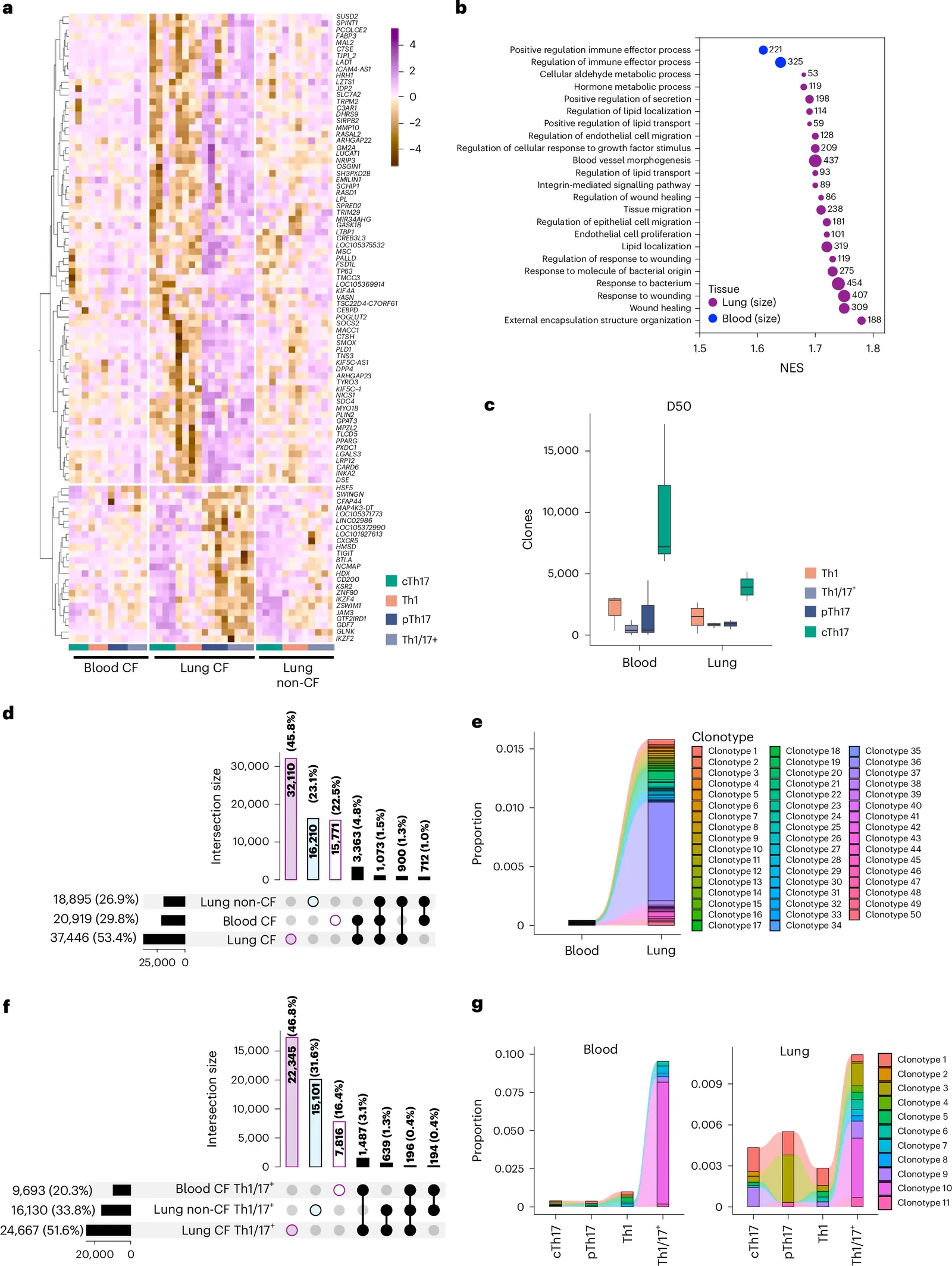
Distinct transcriptional signatures and clonal TCR expansions define pathogenic Th1/17^+^ and pTh17 cells in CF lungs. **a**, Heat map showing the CF lung-specific pathogenic transcriptional signature of pTh17 and Th1/17^+^ cells. The filtering strategy used to identify lung-specific transcriptional signatures and statistical analysis applied are described in Methods. Genes are shown as rows and samples as columns. Expression values represent mean-centred and scaled log_2_(counts per million). Hierarchical clustering was applied to genes only; samples are shown without clustering. All CF samples were derived from patients with chronic *P. aeruginosa* infection (CF blood *n* = 3; CF lung *n* = 4; non-CF lung *n* = 4). **b**, Lollipop plot showing GSEA enrichment of Gene Ontology Biological Process terms in peripheral or lung-resident pTh17 and Th1/17^+^ cells isolated from patients with CF. Enriched terms are ranked by normalized enrichment score (NES) on the *Y*-axis, with NES values shown on the *X*-axis. The circle size indicates the gene set size. **c**, TCR clonal diversity of indicated T cell subsets isolated from CF blood (*n* = 3) or *P. aeruginosa*-infected CF lungs (*n* = 4). Box plots show the D50 index, representing the minimum number of unique clonotypes accounting for 50% of the total TCR repertoire. Data are presented as median ± IQR. **d**, UpSet plot showing overlap of TCR clonotypes among non-CF lungs (*n* = 4), CF blood (*n* = 3) and CF lungs (*n* = 4). Left bars indicate the total number of clonotypes in each group (with percentages relative to the total repertoire), while top bars show the size of intersections between groups, with exact counts and percentages indicated. **e**, Stacked bar plots showing tissue distribution of expanded TCR clonotypes in the CF blood (*n* = 3) and lung of patients with CF (*n* = 4). Each colour corresponds to individual TCR clonotypes (legend in the right panel). **f**, UpSet plot showing overlap of Th1/17^+^ clonotypes among CF blood (*n* = 3), non-CF lung (*n* = 4) or CF lungs (*n* = 4) samples. Left bars indicate the total number of clonotypes per group (with percentages relative to the total repertoire), while top bars show the intersection sizes between groups, with exact counts and percentages indicated. **g**, Stacked bar plots showing the relative abundance of dominant clonotypes across indicated T cell subsets isolated from the blood (left) or lungs (right) of patients with CF. Each colour corresponds to an individual clonotype (listed in the right panel), highlighting subset- and tissue-specific clonal expansions. Source data

RNA sequencing revealed a distinctive transcriptional signature of pulmonary pathogenic Th1/17^+^ and pTh17 cells with 210 genes selectively upregulated and 40 downregulated in *P. aeruginosa*^+^ CF lungs, compared with both CF blood and non-CF lungs (Fig. 2a). To assess whether these pathogenic IFNγ^+^Th17 subsets show CF compartment-dependent transcriptional programmes, we performed a secondary analysis restricted to people with CF (pwCF), comparing pathogenic (Th1/17^+^ and pTh17) and protective (Th1 and cTh17) subsets isolated from either blood or lungs.

**Extended Data Fig. 4:**
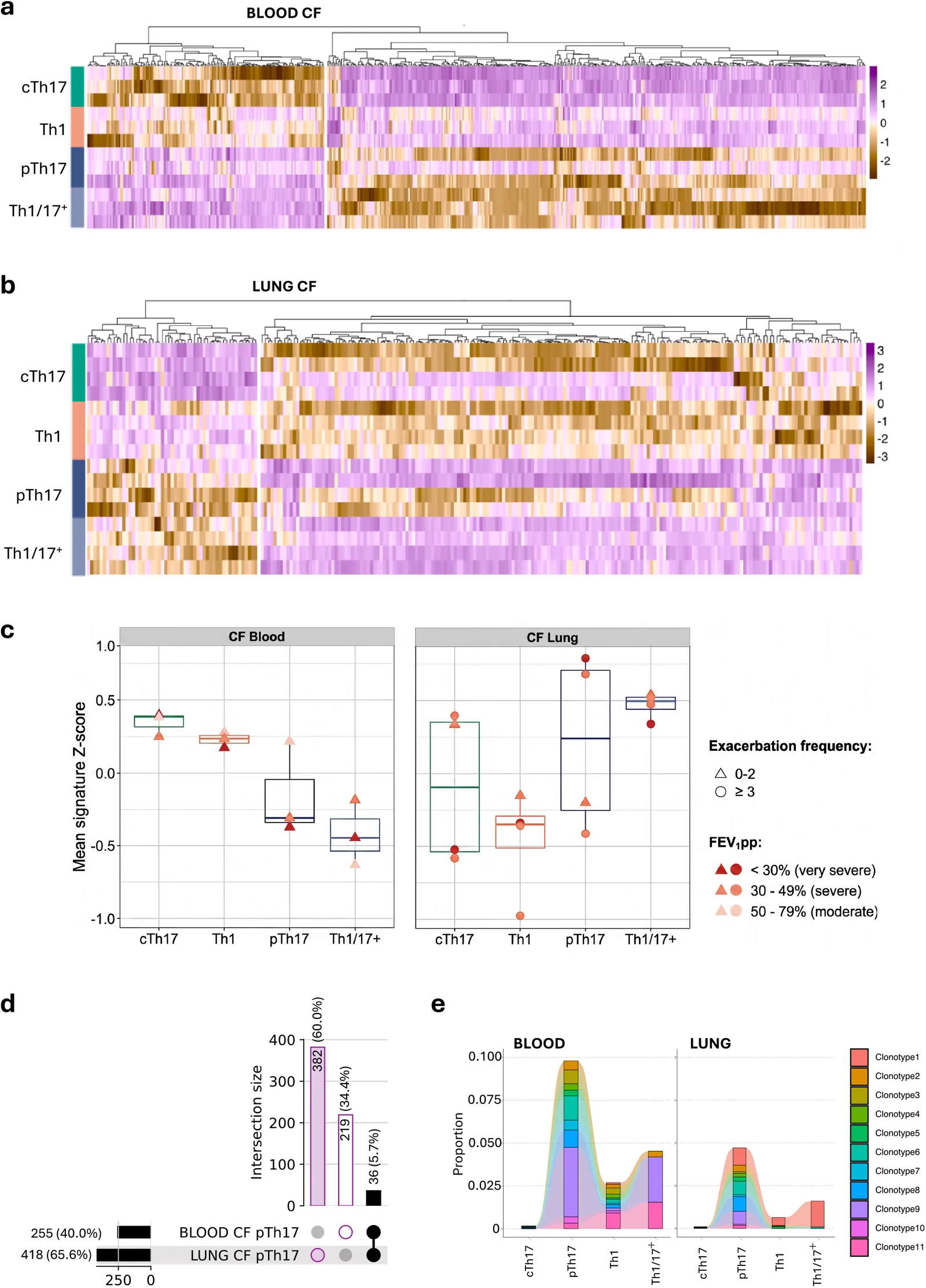
Pulmonary Th1/17^+^ and pTh17 cells in CF exhibit unique gene expression signatures and restricted clonal TCR expansions. **a-b)** Heatmap of differential gene expression in pathogenic (pTh17 and Th1/17^+^) versus non-pathogenic (cTh17 and Th1) subsets in (**a**) CF blood (n = 3) or (**b**) CF lung (n = 4). Genes are shown as columns and samples as rows. Expression values are displayed as mean-centered and standardized log_2_counts per million (CPM). Hierarchical clustering was applied to genes only; samples were not clustered. These panels show independent differential expression analyses performed in CF blood and CF lung, respectively, and are therefore based on distinct, compartment-specific gene sets; direct comparison between panels or with Fig. 2a are not meaningful. All samples were derived from CF patients with chronic *P. aeruginosa lung* infection. **c**) Boxplots showing the distribution of per-sample Z-scores derived from CF compartment-specific DEGs across indicated T cell subsets in CF blood (n = 3) and CF lung (n = 4). Within each boxplot, individual points represent single samples. Boxes represent the interquartile range (IQR), with the centre line indicating the median and whiskers extending to 1.5× IQR. **d**) Overlap of pTh17 clonotypes between blood and lung of CF patients. UpSet plot illustrating the intersection of TCR clonotypes within the pTh17 cells isolated from CF blood (n = 3) or CF lung (n = 4). Left bars indicate the total number of clonotypes detected in each group (with percentages relative to the total repertoire), while top bars represent the intersection size between groups, with exact counts and percentages indicated. **e**) Distribution of expanded clonotypes across Th subsets in blood and lung. Stacked bar plots show the relative abundance of TCR clonotypes cTh17, pTh17, Th1, and Th1/17^+^ cells isolated from blood (left, n = 3) and lung (right, n = 4) of CF patients. Each colour represents a distinct clonotype (listed in the right panel), highlighting the heterogeneity and subset-specific distribution of expanded clones. Source data

Notably, peripheral CF-derived Th1/17^+^ and pTh17 cells showed a predominance of downregulated differentially expressed genes (DEGs), whereas their lung-derived counterparts showed widespread gene upregulation (Extended Data Fig. 4a,b and Supplementary Data 1 and 2), indicating a marked compartment-specific transcriptional divergence. However, these compartment-specific transcriptional modules were not associated with clinical features in either compartment (Extended Data Fig. 4c).

Gene set enrichment analyses (GSEA) of Gene Ontology terms and Kyoto Encyclopedia of Genes and Genomes pathways revealed that only two pathways were significantly upregulated in CF peripheral Th1/17^+^ and pTh17 cells, both linked to immune effector regulation and including high-ranking genes such as *IL23R* and *IL1R1* (Fig. 2b). Conversely, 21 pathways were significantly upregulated in CF lungs, predominantly linked to bacterial response, inflammation and tissue remodelling, including top-ranking genes such as *PPARG*, *CEBPD* and *IRAK2*—transcriptional regulators differently implicated in regulating Th17 cell differentiation and function^32–34^.

Analysis of the Diversity 50 (D50) index of TCR repertoires, performed on the same T cell subsets used for transcriptomic profiling, revealed a significant reduction in clonal diversity in Th1 and cTh17 cells isolated from CF lungs compared with CF blood, indicative of a selective clonal expansion (Fig. 2c). Conversely, Th1/17^+^ cells showed slightly more elevated D50 index values in CF lungs, suggesting a broader clonal repertoire. A comparative clonotype profiling among CF and non-CF samples indicated a significant expansion of private clonotypes within the CF lung (45.8%), exceeding those observed in CF blood (23.1%), and non-CF lung (22.5%) (Fig. 2d). Within the top 50 most expanded clonotypes in CF lungs, some specific sequences showed marked dominance, indicating a selective expansion of pathogenic antigen-specific T cells (Fig. 2e). Next, by excluding clonotypes shared with Th1/17^+^ cells from CF blood and non-CF lungs, we identified 22,345 (46.8%) clonotypes uniquely enriched in pulmonary Th1/17^+^ cells compared with 7,816 (16.4%) exclusively enriched in their peripheral counterpart (Fig. 2f). By contrast, pTh17 cells showed fewer unique clonotypes in both lungs (60%) and blood (34.4%) (Extended Data Fig. 4d). Notably, among the 22,345 lung-restricted Th1/17^+^ clonotypes, the top 11 most expanded showed distinct distributions across subsets (Fig. 2g): some were exclusively found in Th1/17^+^ cells (for example, clonotype 10), while others shared with pTh17 but minimally expanded in Th1 or cTh17 subsets (for example, clonotype 3), suggesting common antigenic experience and functional convergence. A similar pattern was observed among the predominant pTh17 lung clonotypes, several of which overlapped with Th1/17^+^ cells (Extended Data Fig. 4e), indicating shared antigenic imprinting.

Collectively, these findings reveal that pathogenic Th1/17^+^ and pTh17 cells in CF lungs are transcriptionally reprogrammed and clonally selected in situ.

### Clinical *P. aeruginosa* strains persist within DCs, driving the secretion of polarizing cytokines linked to IFNγ^+^Th17 cell generation

**Fig. 3:**
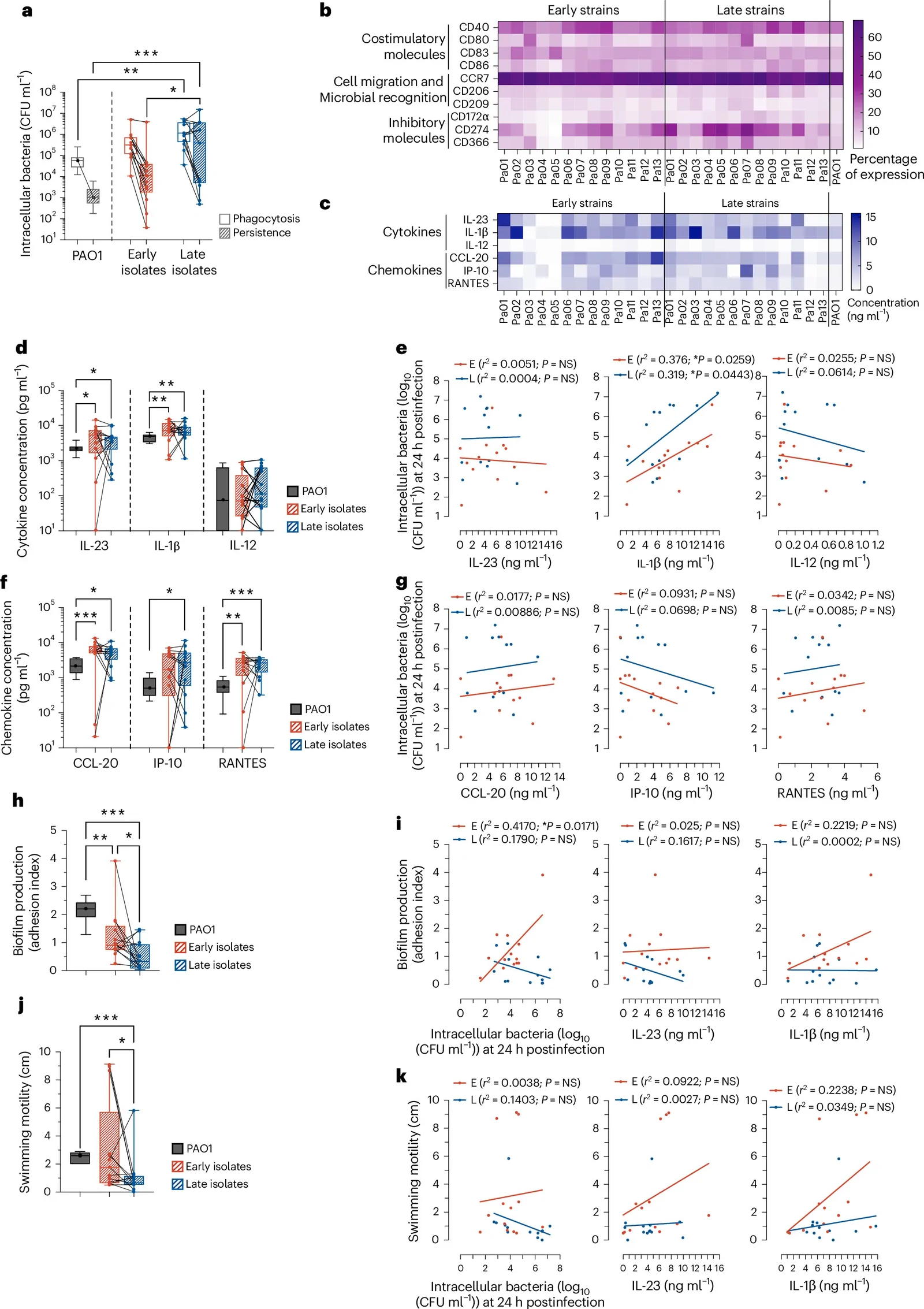
Clinical *P. aeruginosa* isolates stimulate a specific pro-inflammatory cytokine profile, dominated by IL-1β, IL-23 and CCL20, linked to IFNγ^+^Th17 cell generation. **a**, Intracellular bacterial load of the *P. aeruginosa* reference strain PAO1 and clinical clonal strains, isolated from the lungs of patients with CF either at disease onset (Early) or after long-term persistence (Late), assessed in human HD-derived moDCs at 90 min (phagocytosis) or 24 h (intracellular persistence) postinfection. Individual dots represent the mean values of 19 independent experiments for PAO1, and 6–11 independent experiments for individual early or late *P. aeruginosa* isolates. **b**, Heat map showing the mean percentage of marker-positive DCs for a panel of co-stimulatory molecules, chemokine and pattern-recognition receptors, and inhibitory molecules, in human HD-derived moDCs at 24 h postinfection with the indicated *P. aeruginosa* clinical isolates. Data are expressed as mean percentage of positive DCs for each marker, calculated across four independent donors. **c**, Heat map of the full panel of cytokines and chemokines (rows) released by HD-derived moDCs after 24 h of infection with clinical clonal *P. aeruginosa* isolates (early versus late) or with PAO1 (columns). Data are expressed as the mean amount of indicated cytokine and chemokine concentrations (ng ml^−1^), calculated across 19 independent experiments for PAO1, and 6–11 independent experiments for individual early or late *P. aeruginosa* isolates. **d**,**f**, Concentration of polarizing cytokines IL-23, IL-1β and IL-12 (**d**) and chemokines CCL-20, IP-10 and RANTES (**f**) secreted by moDCs infected with PAO1 or clinical *P. aeruginosa* isolates (early versus late). Individual dots represent the mean values of 19 independent experiments for PAO1, and 6–11 independent experiments for individual early or late *P. aeruginosa* isolates. **e**,**g**, Correlation between cytokine (**e**) or chemokine (**g**) concentration after 24 h of infection and intracellular persistence of clinical *P. aeruginosa* early (E) and late (L) strains within human moDCs. **h**, Biofilm production by the reference PAO1 strain and clonal *P. aeruginosa* isolates (early versus late). Individual dots represent the mean values of 12 independent experiments for PAO1, and 4–6 independent experiments for clinical *P. aeruginosa* isolates. **j**, Swimming motility of the reference PAO1 strain and clonal *P. aeruginosa* isolates (early versus late). Individual dots represent the mean values of three independent experiments for each *P. aeruginosa* strain. **i**,**k**, Correlation between biofilm formation (**i**) or motility (**k**) of early (E) and late (L) clinical *P. aeruginosa* strains and their intracellular bacterial load within moDCs, IL-23 and IL-1β secretion, at 24 h postinfection. Data (**a**, **d**, **f**, **h** and **j**) are presented as box-and-whisker plots showing the median (centre line) and minimum-to-maximum range (whiskers). Data of clonal strains isolated from the same patient with CF are connected by lines. Statistical significance was calculated using one-way ANOVA and reported as *P < 0.05, **P < 0.01 and ***P < 0.001. Positive or negative correlations (**e**, **g**, **i** and **k**) were determined using Pearson analysis. Correlation coefficients (*r*^2^) together with *P* values are reported in the graphs. Source data

**Extended Data Fig. 5:**
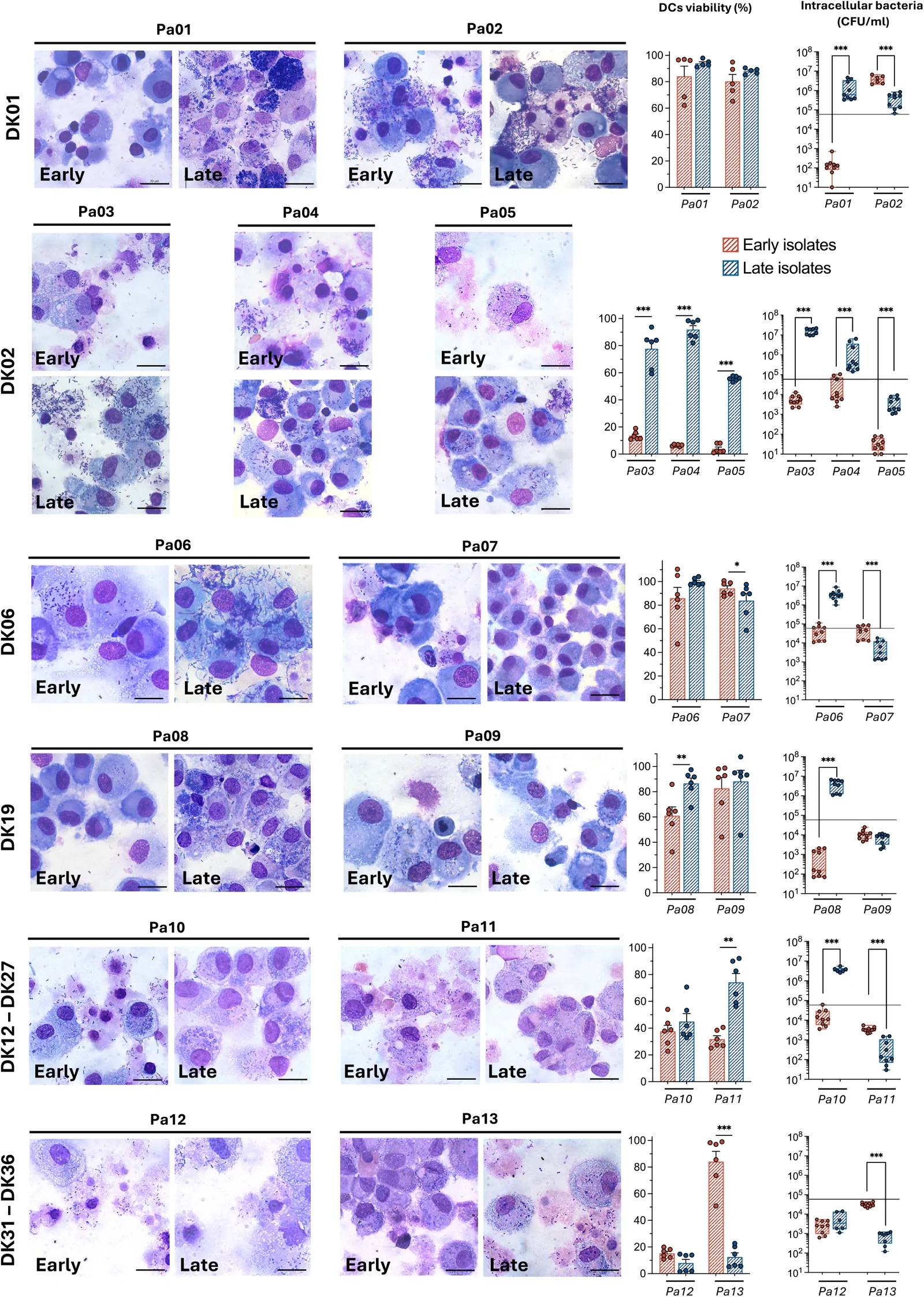
Intracellular persistence and host cytotoxicity of clinical *P. aeruginosa* isolates in human moDCs. Representative images from 3 independent experiments of Giemsa-stained human HD-derived moDCs after 24 h of infection with clinical *P. aeruginosa* strains isolated from lungs of CF patients, either at disease onset (Early) or after long-term persistence (Late). *P. aeruginosa* strains are grouped by CF patient origin and clonotype (DK). Scale bars: 20 µm, original magnification x1000. Bar graphs displayed alongside each row of Giemsa-stained images depict: - HD-derived moDCs viability, measured by WST-1 assay, after 24 h of infection with individual clonal *P. aeruginosa* clinical strains (early vs late). Viability is expressed relative to uninfected control moDCs (set to 100%). Bars represent mean ± SEM and individual dots the mean values of 5-6 independent experiments for each strain. - the intracellular bacterial load (CFU/ml) of individual clonal *P. aeruginosa* clinical strains (early vs late), obtained by serial dilution of moDC lysates at 24 hours post-infection. Data are presented as box-and-whisker plots showing the median (center line), minimum-to-maximum range (whiskers) and individual data points from 6−11 independent experiments. Statistical analysis was performed using Mann-Whitney test for paired clonal *P. aeruginosa* strains and reported as * *p* < 0.05, ** *p* < 0.01, *** *p* < 0.001. Source data

We next investigated whether *P. aeruginosa* can promote the upstream processes driving pathogenic IFNγ^+^Th17 cell generation by assessing the immunogenicity of 26 *P. aeruginosa* strains longitudinally isolated from CF lungs^35^ (Supplementary Table 5) in HD monocyte-derived dendritic cells (moDCs). Clinical *P. aeruginosa* strains collected after years of lung colonization (late isolates) were more efficiently internalized by DCs and showed enhanced intracellular persistence, compared with the reference strain PAO1 and with early clinical isolates (Fig. 3a), without affecting DC viability (Extended Data Fig. 5). Of note, increased intracellular persistence within DCs was not restricted to specific clonotypes and unrelated to the duration of lung colonization in pwCF (Extended Data Fig. 5 and Supplementary Table 5).

**Extended Data Fig. 6:**
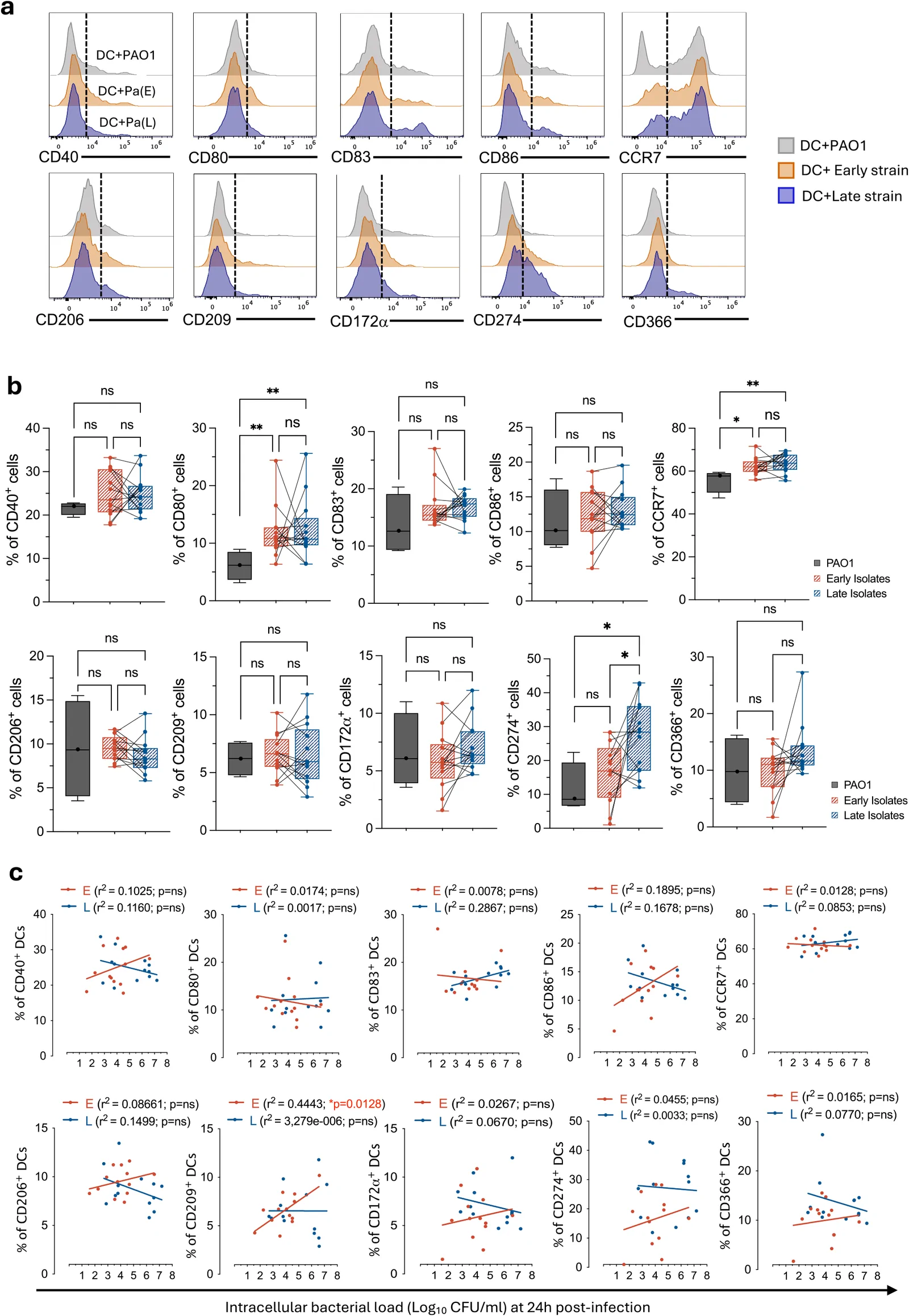
Differential activation of human DCs in response to early and late *P. aeruginosa* clinical isolates. **a)** Representative histogram overlays showing expression levels of the indicated surface markers in HD-derived moDCs infected with PAO1 or clonal early (E) or late (L) *P. aeruginosa* clinical strains. **b)** Percentage of HD-derived moDCs expressing the indicated marker following 24 h of infection with the reference strain PAO1 or *P. aeruginosa* clinical strains isolated at disease onset (Early) or after long-term persistence (Late) in CF lungs. Data are presented as box-and-whisker plots showing the median (center line) and minimum-to-maximum range (whiskers). Individual dots represent the mean values of 4 independent experiments. Data of clonal strains isolated from the same CF patient are connected by lines. Statistical significance was calculated using one-way ANOVA and reported as * *p* < 0.05, ** *p* < 0.01. **c)** Correlation analysis between expression levels of the indicated surface markers in infected HD-derived moDCs and intracellular bacterial load of clonal *P. aeruginosa* clinical strains (early vs late) at 24 h post-infection. Positive or negative correlations between variables were evaluated using Pearson correlation analysis. R^2^ and p values are indicated within each graph. Source data

To gain mechanistic insight into DC functional states, we next analysed whether clinical isolates modulate DC activation and inflammatory response. Compared with PAO1, clinical isolates induced a significantly higher expression of CD80 and CCR7 in infected moDCs, with comparable levels between early and late isolates. By contrast, late strains induced significantly higher expression of the inhibitory molecule CD274 (Fig. 3b and Extended Data Fig. 6a,b). Interestingly, clinical strains elicited an imbalanced cytokine response by DCs (Fig. 3c), stimulating a greater release of IL-23 and IL-1β, key drivers of IFNγ^+^Th17 cells, rather than of the Th1-polarizing cytokine IL-12 (Fig. 3d). However, exclusively, IL-1β levels positively correlated with the intracellular bacterial persistence (Fig. 3e). Similarly, compared with PAO1, clinical isolates triggered higher chemokine secretion, particularly CCL20, which is linked to DCs and CCR6^+^ cell recruitment (Fig. 3f), although their levels did not correlate with intracellular persistence (Fig. 3g). Likewise, intracellular bacterial persistence was unrelated to increased expression of the surface markers analysed, except for CD209, which showed a significant positive correlation exclusively in response to early isolates (Extended Data Fig. 6c).

**Extended Data Fig. 7:**
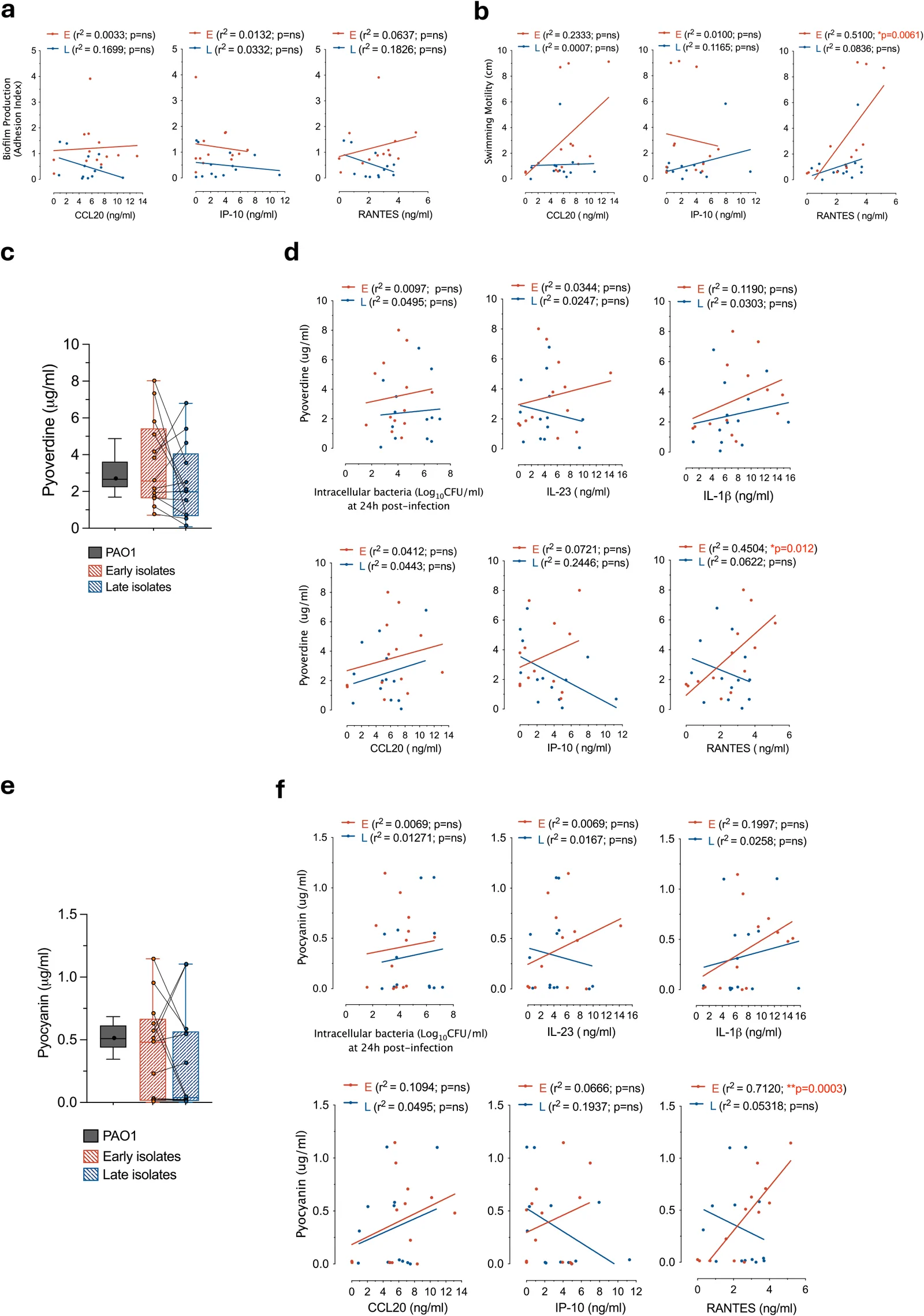
Correlation analysis between virulence-associated traits of clinical *P. aeruginosa* isolates and host responses. **a-b)** Correlation analysis between biofilm production (**a**), or swimming motility (**b**), of clonal *P. aeruginosa* clinical strains (early vs late) and indicated chemokine concentration secreted by HD-derived moDCs at 24 h post-infection. Positive or negative correlations between variables were determined using Pearson correlation analysis. R squares together with p values for each correlation were reported in the graphs. **c)** Quantification of pyoverdine production by the reference strain PAO1 and clonal *P. aeruginosa* clinical isolates (early vs late). Data are presented as box-and-whisker plots showing the median (center line) and minimum-to-maximum range (whiskers). Individual dots represent the mean values of 10 independent experiments for PAO1 and of 5–11 independent experiments for individual early or late *P. aeruginosa* isolates. **d)** Correlation analysis between pyoverdine production of clonal *P. aeruginosa* clinical strains (early vs late) and intracellular bacterial load (top left), IL-23 and IL-1β secretion (top center), and indicated chemokine levels (bottom panels). Positive or negative correlations between variables were evaluated using Pearson correlation analysis. R^2^ together with p values are indicated within each graph. **e)** Quantification of pyocyanin production by the reference strain PAO1 and clonal *P. aeruginosa* clinical isolates (early vs late). Data are presented as box-and-whisker plots showing the median (center line) and minimum-to-maximum range (whiskers). Individual dots represent the mean values of 14 independent experiments for PAO1 and of 5–11 independent experiments for individual early or late *P. aeruginosa* isolates. **f)** Correlation analysis between pyocyanin production of clonal *P. aeruginosa* clinical strains (early vs late) and intracellular bacterial load (top left), IL-23 and IL-1β secretion (top center), and indicated chemokine levels (bottom panels). Positive or negative correlations between variables were evaluated using Pearson correlation analysis. R^2^ and p values are indicated within each graph. Source data

We next evaluated whether these immunostimulatory traits were associated with common *P. aeruginosa* virulence adaptations^36^. Consistent with literature data^37^, late strains showed reduced biofilm formation and motility compared with both PAO1 and early strains (Fig. 3h,j). Biofilm formation showed distinct correlation patterns between early and late isolates, with differential associations with intracellular persistence, cytokine production, chemokine levels and DC activation markers (Fig. 3i, Extended Data Fig. 7a and Supplementary Fig. 2a). Similarly, motility in early and late strains showed differential associations with intracellular persistence and cytokine and chemokine production, while showing similar associations with DC activation markers, with the exception of CD206 (Fig. 3k, Extended Data Fig. 7b and Supplementary Fig. 2b). No significant differences were observed in pyoverdine or pyocyanin production among clinical isolates (Extended Data Fig. 7c,e), although opposite trends in correlation between these virulence factors and the levels of IL-23, IP-10 and regulated on activation, normal T-cell expressed and secreted (RANTES) were evident between early and late strains (Extended Data Fig. 7d,f). Overall, these results show that *P. aeruginosa* strains can efficiently persist within DCs and drive a coordinated remodelling of DC activation together with an IL-1β/IL-23-skewed inflammatory programme linked to IFNγ^+^Th17 cell differentiation. This process probably begins during the early stage of *P. aeruginosa* infection and may substantially contribute to sustained CF lung inflammation.

### Clinical *P. aeruginosa* strains reprogramme protective cTh17 cells into pathogenic IFNγ^+^Th17 cells

**Fig. 4:**
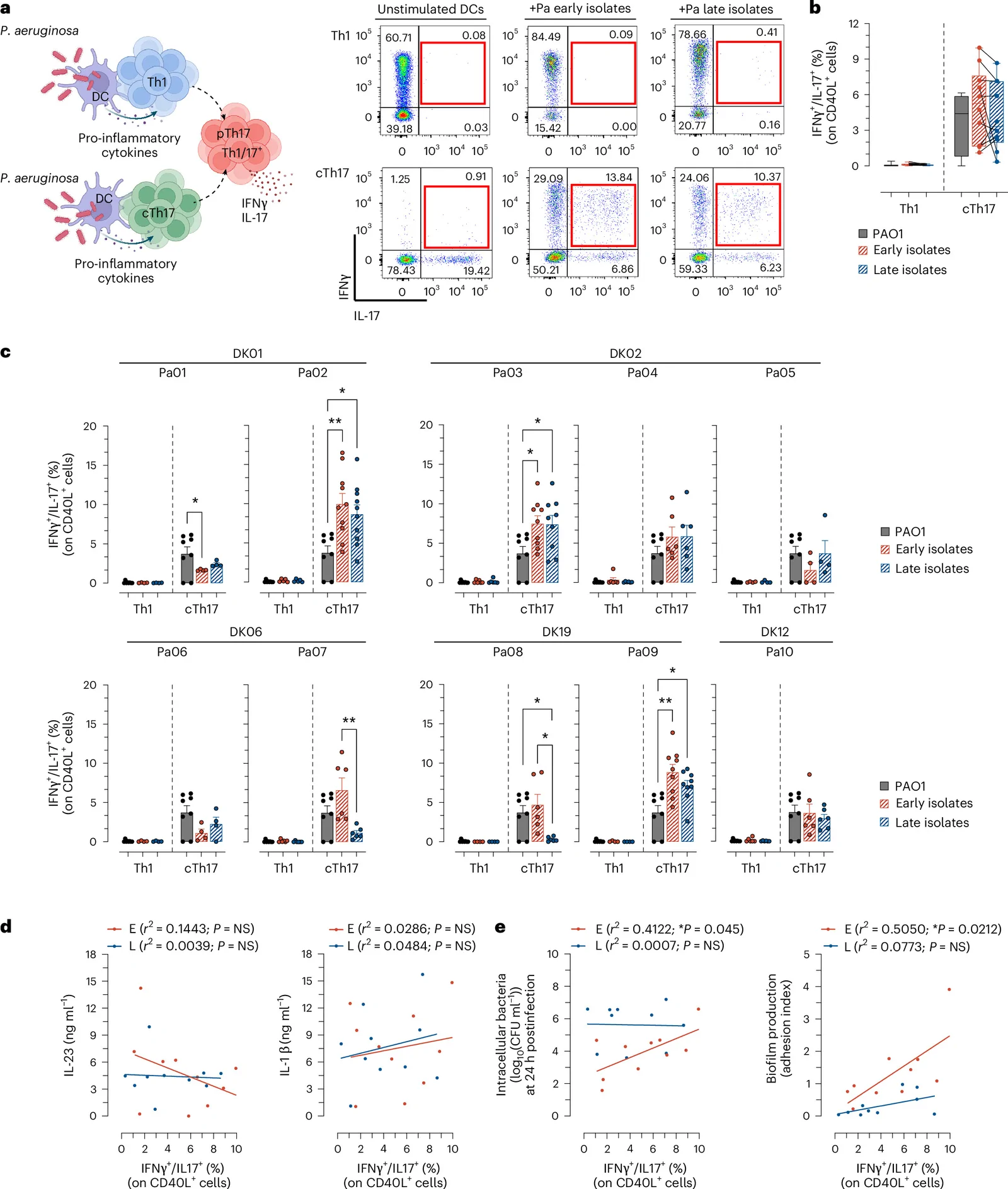
Clinical *P. aeruginosa* isolates, regardless of CF lung adaptation stage, induce the transdifferentiation of pathogenic IFNγ^+^Th17 cells exclusively from conventional Th17 precursors. **a**, Schematic representation of the experimental strategy for assessing *P. aeruginosa*-induced transdifferentiation of peripheral Th1 or cTh17 into IFNγ^+^-producing Th17 cells. Sorted Th1 or cTh17 cells were co-cultured with autologous moDCs infected with clonal *P. aeruginosa* clinical strains (Early versus Late). After 10 days, IFNγ^+^/IL-17^+^ co-expression was evaluated by flow cytometry. **b**, Mean percentage of IFNγ^+^/IL-17^+^ co-expression among peripheral CD40L^+^ Th1 or cTh17 cells after 10 days of co-culture with autologous HD-derived moDCs infected with the reference strain PAO1 or clonal *P. aeruginosa* clinical isolates (early versus late). Data are presented as median ± IQR from at least five independent experiments per strain. Data are presented as box-and-whisker plots showing the median (centre line) and minimum-to-maximum range (whiskers). Individual dots represent the mean values of eight independent experiments for PAO1, and four to ten independent experiments for individual early or late *P. aeruginosa* isolates. Data of clonal strains isolated from the same patient with CF are connected by lines. **c**, Frequency of IFNγ^+^/IL-17^+^ double-positive cells among peripheral CD40L^+^ Th1 or cTh17 cells following 10 days of co-culture with autologous HD-derived moDCs infected with individual *P. aeruginosa* clinical strains isolated from CF lungs, either at disease onset (Early) or after long-term persistence (Late). *P. aeruginosa* strains are grouped by origin of the patient with CF and clonotype (DK). Bars represent mean ± s.e.m., and individual dots represent the mean values of eight independent experiments for PAO1, and four to ten independent experiments for individual early or late *P. aeruginosa* isolates. Statistical significance was calculated using one-way ANOVA and reported as *P < 0.05 and **P < 0.01. **d**, Pearson correlation analysis between the frequencies of IFNγ^+^/IL-17^+^ co-expression on peripheral CD40L^+^ cTh17 cells and IL-23 (left panel), or IL-1β levels (right panel), secreted by *P. aeruginosa-*infected HD-derived moDCs after 10 days of co-culture. Correlation coefficients (*r*^2^) and *P* values are indicated in each graph. **e**, Pearson correlation analysis between the percentage of IFNγ^+^/IL-17^+^ co-expression on peripheral CD40L^+^ cTh17 cells and the intracellular persistence of clonal *P. aeruginosa* clinical isolates (early versus late) at 24 h postinfection (left panel), or biofilm production (right panel). The *r*^2^ and *P* values for each correlation are reported in the graphs. Schematic in **a** created in BioRender; Paroni, M. https://biorender.com/is0fbd8 (2026). Source data

**Extended Data Fig. 8:**
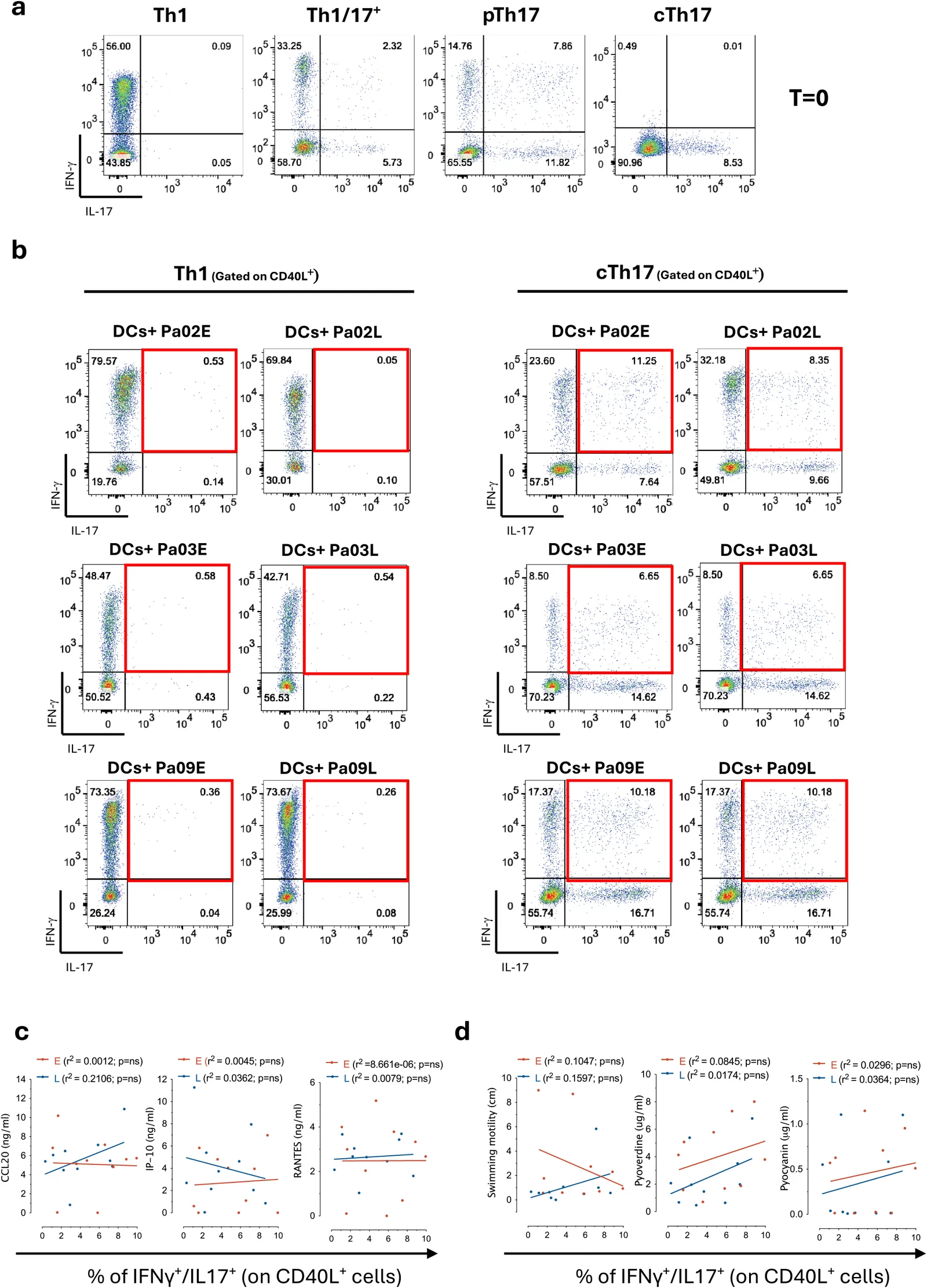
Functional skewing of cTh17 induced by clinical *P. aeruginosa* strains occurs independently of bacterial virulence traits or chemokine levels. **a)** Representative flow cytometry analysis (out of 6 independent experiments) showing the frequency of IFNγ^+^, IL-17^+^ and IFNγ^+^/IL-17^+^ double-positive cells among ex-vivo (T = 0) polyclonally stimulated peripheral Th1, Th1/17^+^, pTh17, and cTh17 cells, sort-purified from peripheral blood of HD patients. Cytokine expression was assessed by intracellular staining with flow cytometry. **b)** Representative flow cytometry analysis (out of 4–10 independent experiments) showing the frequency of IFNγ^+^/IL-17^+^ co-expressing cells among peripheral Th1 (left panel) or cTh17 cells (right panel), sort-purified from blood of HD, and co-cultured for 10 days with autologous moDCs infected with individual clonal *P. aeruginosa* clinical isolates collected at early (**e**) or late (**l**) stages of CF lung colonization. **c)** Correlation analysis between concentrations of indicated chemokines and the percentage of IFNγ^+^/IL-17^+^ co-expressing CD40L^+^ cTh17 cells after 10 days of co-culture with autologous HD-derived moDCs infected with clinical *P. aeruginosa* strains (Early vs Late). **d)** Correlation analysis between *P. aeruginosa* phenotypes - namely swimming motility, pyoverdine, and pyocyanin production - and the frequency of IFNγ^+^/IL-17^+^ co-expression on peripheral CD40L^+^ cTh17 cells after 10 days of co-culture with autologous infected HD-derived moDCs. Positive or negative correlations (**c** and **d**) between variables were evaluated using Pearson correlation analysis. R^2^ together with p values are indicated within each graph. Source data

Here we sought to identify the T cell precursors of pathogenic IFNγ^+^Th17 and determine whether early or late clinical *P. aeruginosa* strains differentially promote their generation using an antigen-specificity in vitro assay with HD-derived peripheral Th1 and cTh17 cells (Fig. 4a), and a subset of *P. aeruginosa* strains representative of prevalent Danish CF clonal lineages (Supplementary Table 5). Our data unequivocally showed that Th1 cells failed to transdifferentiate into IFNγ^+^Th17 cells in response to any *P. aeruginosa* strain tested (Fig. 4b,c). By contrast, cTh17 cells emerged as the principal precursors of Th1/17^+^ and pTh17 cells, showing strong IFNγ/IL-17 co-expression following stimulation with *P. aeruginosa* (Fig. 4b,c), irrespective of the duration of bacterial colonization of the CF lungs (Supplementary Table 5). Notably, while no specific *P. aeruginosa* clonotype uniquely drove cTh17-to-IFNγ^+^Th17 conversion, some isolates, particularly Pa02, Pa03 and Pa09, elicited significantly higher levels of IFNγ/IL-17 co-production than PAO1 (Fig. 4c), even exceeding levels observed in ex vivo polyclonally activated Th1/17^+^ and pTh17 cells (Extended Data Fig. 8a,b). Unexpectedly, late isolates were not more effective in promoting IFNγ^+^Th17 cell differentiation. Rather, early isolates, including Pa02E, Pa07E, Pa08E and Pa09E, elicited higher IFNγ/IL-17 co-expression than their late-stage clonal counterparts (Fig. 4c).

To explore possible links between *P. aeruginosa* virulence determinants and Th17 cell reprogramming, we correlated IFNγ/IL-17 co-production with strain-specific immunogenicity. Unexpectedly, neither IL-1β, IL-23 nor other examined chemokines released by *P. aeruginosa*-infected DCs significantly correlated with IFNγ/IL-17 co-production (Fig. 4d and Extended Data Fig. 8c). By contrast, IFNγ/IL-17 co-production significantly correlated with intracellular persistence within DCs and the biofilm-forming capacity of *P. aeruginosa* exclusively in early isolates (Fig. 4e). Conversely, no correlation was observed between IFNγ/IL-17 co-production and other virulence factors, including swimming motility and pyoverdine or pyocyanin production (Extended Data Fig. 8d).

Taken together, these findings uncover a direct and potent capacity of *P. aeruginosa* to reprogramme protective cTh17 into pathogenic IFNγ^+^Th17 cells, a property evident even in early clinical strains lacking phenotypic traits typically acquired by late isolates during chronic infection in CF lungs.

### Pathogenic reprogramming of cTh17 cells by *P. aeruginosa* recapitulates CF lung Th1/17^+^ and pTh17 profiles

**Extended Data Fig. 9:**
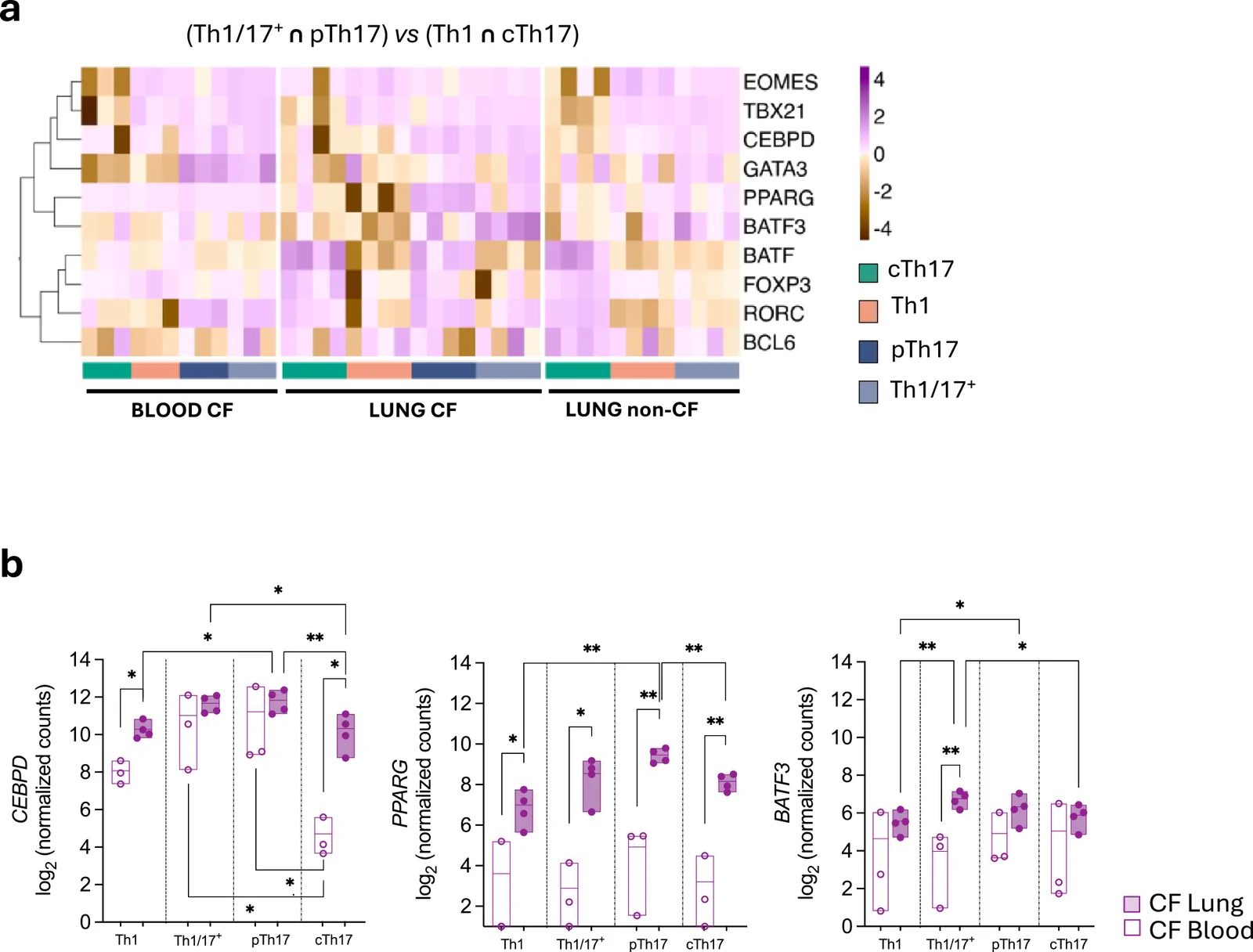
Pathogenic pulmonary Th1/17^+^ and pTh17 cells in CF exhibit selective upregulation of transcriptional regulators. **a**) Heatmap of expression patterns of selected key transcription factors between T cell subsets in the lung (n = 4) and blood (n = 3) of CF patients, and in the lung of non-CF patients (n = 4). Genes are shown on the rows, and samples are shown on the columns. Colours indicate subset-type annotations. Expression values are presented as mean-centered and standardized log_2_ counts per million (CPM). **b**) Log_2_ of normalized counts of selected transcription factors (*CEBPD*, *PPARG*, and *BATF3*) across the indicated T-cell subsets isolated from lungs (n = 4) or peripheral blood (n = 3) of CF patients. Statistical significance was calculated using one-way ANOVA within tissue comparisons, and Unpaired t test with Welch’s correction between the same T-cell subset across tissues, and reported as * *p* < 0.05, ** *p* < 0.01. Source data

We next evaluated whether *P. aeruginosa*-driven skewing of cTh17 towards the IFNγ^+^Th17 phenotype was associated with the upregulation of transcription factors identified by ex vivo transcriptomic analysis as enriched in CF lung-resident Th1/17^+^ and pTh17 cells (Fig. 2a and Extended Data Fig. 9a).

**Fig. 5:**
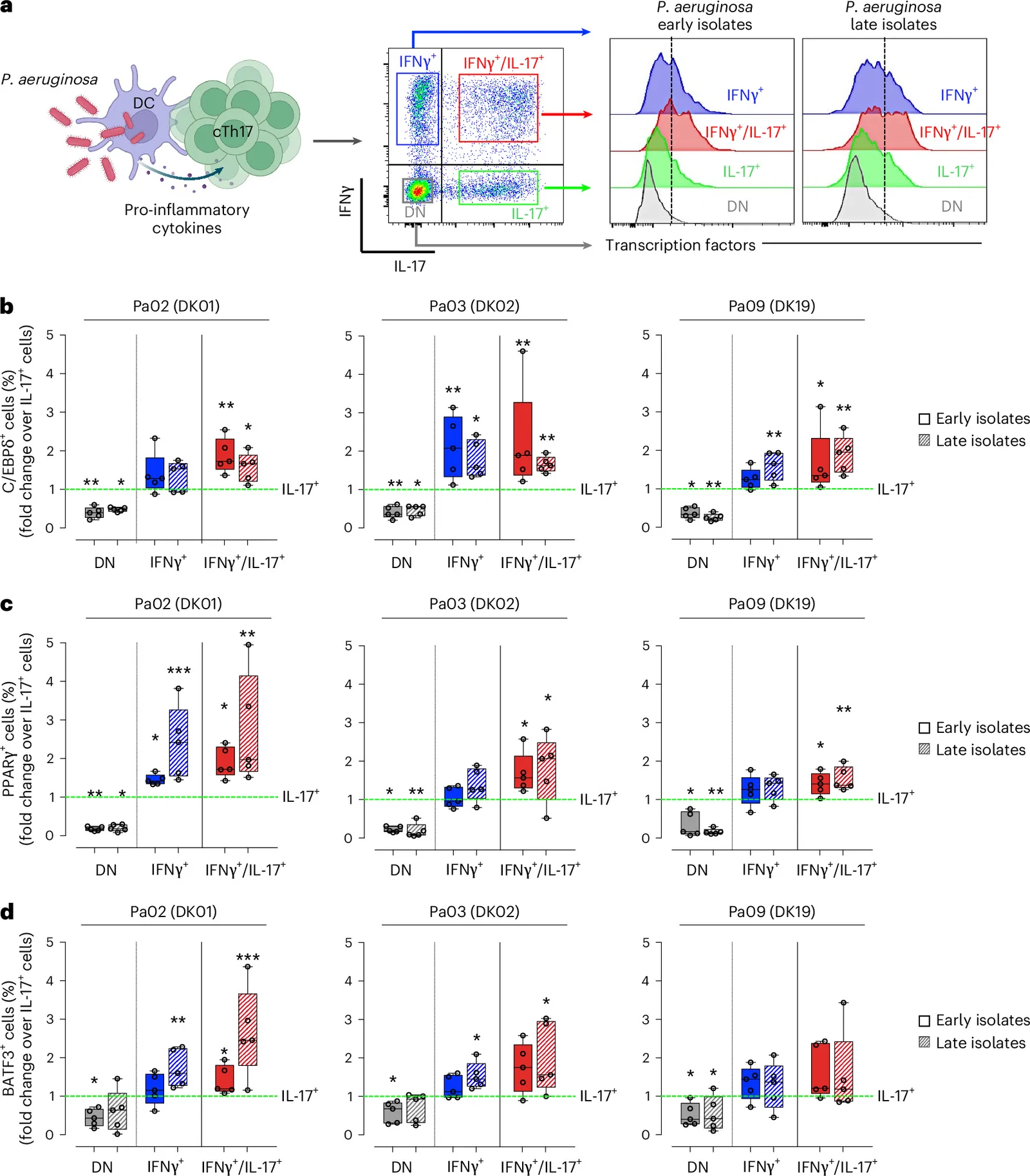
*P. aeruginosa* drives the skewing of protective cTh17 into pathogenic IFNγ^+^Th17 subsets recapitulating the transcriptional programme of lung-infiltrating Th1/17^+^ and pTh17 subsets in CF. **a**, Schematic representation of the experimental strategy used to evaluate the upregulation of selected transcription factors in *P. aeruginosa*-activated cTh17 cells. Sorted peripheral cTh17 cells from patients with CF were co-cultured for 10 days with autologous CF-derived moDCs infected with specific clonal *P. aeruginosa* clinical strains (early versus late) previously shown to induce the highest transdifferentiation into IFNγ^+^Th17 cells. Intranuclear expression of the indicated transcription factors was assessed by flow cytometry on gated IFNγ^+^, IL-17^+^, IFNγ^+^/IL-17^+^ double-positive and IFNγ^−^/IL-17^−^ (DN) cells. **b**–**d**, Fold change in the percentage of C/EBPδ^+^ (**b**), PPARγ^+^ (**c**) and BATF3^+^ (**d**) expression in peripheral CD40L^+^ cTh17 cells after 10 days of co-culture with autologous CF-derived moDCs infected with the indicated clonal *P. aeruginosa* clinical strains (early versus late). Data are expressed as the fold change expression of the indicated transcription factor within IFNγ^+^, IFNγ^+^/IL-17^+^ (double positive) and IFNγ^−^/IL-17^−^ (DN) populations, normalized to the transcription factor expression within IL-17^+^ cells. Data are presented as box-and-whisker plots showing the median (centre line) and minimum-to-maximum range (whiskers). Individual dots represent the mean values of five independent experiments. Statistical significance was calculated using one-way ANOVA and reported as *P < 0.05, **P < 0.01 and ***P < 0.001. Schematic in **a** created in BioRender; Paroni, M. https://biorender.com/r0vdi28 (2026). Source data

Owing to the limited feasibility of ex vivo functional analysis on human lung-resident T cell subsets, we used the antigen-specificity assay using peripheral CF-derived cTh17 cells together with selected clinical *P. aeruginosa* strains showing the highest capacity to induce IFNγ^+^Th17 cell transdifferentiation (Fig. 4c and Extended Data Fig. 8b). We evaluated the expression of CCAAT/enhancer-binding protein delta (C/EBPδ), peroxisome proliferator-activated receptor gamma (PPARγ) and basic leucine zipper ATF-like transcription factor 3 (BATF3) at the protein level, three transcription factors differently implicated in Th17 cell fate and function^32,33,38^ which resulted selectively upregulated in CF lung-resident Th1/17^+^ and pTh17 cells (Extended Data Fig. 9b). Expression was quantified across functionally distinct populations: IFNγ^+^/IL-17^+^, IFNγ^+^/IL-17^−^ and IFNγ^−^/IL-17^−^ double-negative (DN), with IFNγ^−^/IL-17^+^ cells as baseline reference (Fig. 5a).

Intracellular staining revealed that all the *P. aeruginosa* strains tested induced significant upregulation of both C/EBPδ (Fig. 5b) and PPARγ (Fig. 5c), predominantly in IFNγ^+^/IL-17^+^ cells. Notably, this expression pattern closely mirrored the transcriptional profile observed ex vivo in Th1/17^+^ and pTh17 cells isolated from CF lungs (Extended Data Fig. 9a,b). By contrast, only Pa03 (early and late isolates) and Pa09L induced higher expression of C/EBPδ in IFNγ^+^/IL-17^−^ cells (Fig. 5b), whereas PPARγ upregulation in IFNγ^+^/IL-17^−^ cells was uniquely triggered by Pa02, with no concurrent C/EBPδ induction. Strikingly, *BATF3* mRNA expression—which was detected at lower levels than *CEBPD* and *PPARG* in lung-resident Th1/17^+^ and pTh17 (Extended Data Fig. 9b)—was upregulated in IFNγ^+^/IL-17^+^ cells in response to Pa02 (early and late isolates) and Pa03L, but not to Pa09 (Fig. 5d). Importantly, DN cells failed to upregulate any of the transcription factors analysed, further highlighting the specificity of the underlying transcriptional programme.

Together, these results show that *P. aeruginosa* can promote the reprogramming of peripheral cTh17 into IFNγ^+^Th17 cells, activating a transcriptional network that mirrors the pathogenic Th1/17^+^ and pTh17 cell signature observed in CF lungs.

## Discussion

Chronic *P. aeruginosa* infections, together with an exaggerated mucosal immune response, represent hallmarks of CF lung disease and remain the principal drivers of progressive lung function decline^1^. While the impact of *P. aeruginosa* on innate immunity has been extensively characterized^2,4^, its role in reshaping and functionally skewing adaptive T cell responses remains poorly understood.

**Extended Data Fig. 10:**
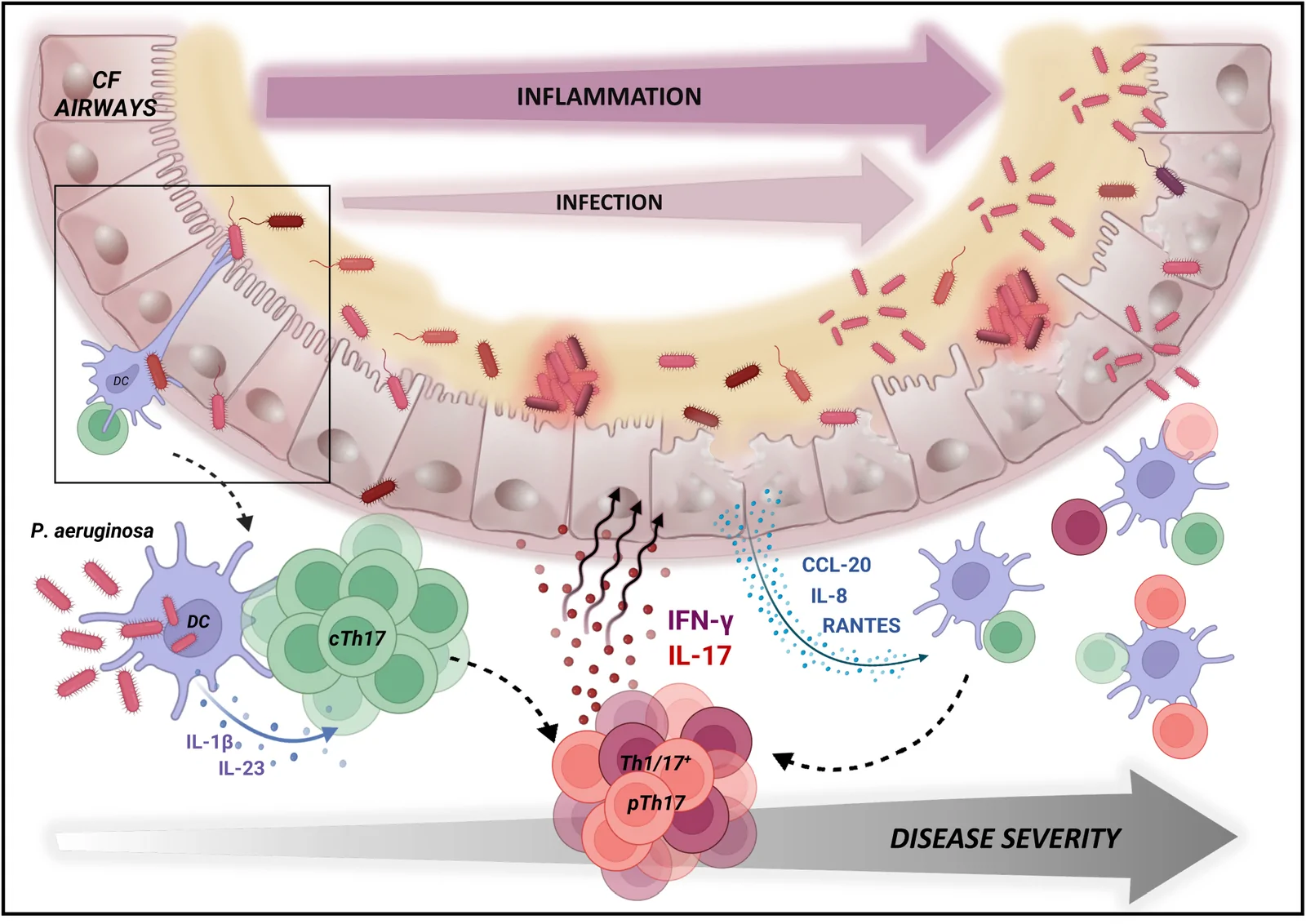
*Pseudomonas aeruginosa* subverts Th17 immunity, driving pathogenic inflammation in CF. *P. aeruginosa* infection reprograms protective conventional Th17 (cTh17) cells into IFNγ-producing pathogenic subsets (Th1/17^+^ and pTh17). These cells are highly enriched in CF lungs, causing epithelial damage and amplifying immune cell recruitment through the release of high IL-17 and IFNγ levels. In response, epithelial cells secrete increased amounts of chemokines (CCL20, IL-8, RANTES) reinforcing a self-sustaining inflammatory loop that links *P. aeruginosa* infection to immune dysregulation and progressive disease severity. (Part of the Figure was created with BioRender.com).

In this study, by combining the ex vivo analysis of the human CF lung with mechanistic in vitro functional models, we have delineated a previously undescribed immunopathological axis in CF, whereby *P. aeruginosa* drives the expansion and functional reprogramming of IFNγ^+^Th17 cells, specifically the Th1/17^+^ and pTh17 subsets, culminating in epithelial barrier disruption and potentially promoting the perpetuation of non-resolving mucosal inflammation (Extended Data Fig. 10).

Several previous studies have debated the role of Th17 cells in CF lung disease^15–17^. Indeed, while IL-17-producing cells mediate protective functions by promoting bacterial clearance and preserving lung structure^18^, excessive and chronic IL-17 secretion is linked to worsened CF airway pathology^17,39^. Here we provided evidence for a distinct immunological and functional subdivision within CF lung-resident Th17 populations. In stark contrast to protective cTh17, pulmonary-resident IFNγ^+^Th17 cells are selectively enriched in CF and show a functional divergence beyond a phenotypic shift: following activation, Th1/17^+^ and pTh17 cells acquire a pathogenic effector profile that directly compromises epithelial barrier integrity and elicits a detrimental pro-inflammatory response from airway epithelial cells in vitro.

Multiple lines of evidence indicate that IFNγ^+^Th17 cell differentiation is driven by elevated IL-1β and IL-23 levels^25–27^, both of which are markedly enriched in the CF lung environment^28–30^. These cytokines have been implicated in the pathogenesis of several chronic inflammatory disorders^40,41^, in which targeted biologic therapies, particularly IL-23 inhibitors, have shown substantial clinical efficacy^40^. In CF, IL-1 receptor blockade with anakinra mitigates *P. aeruginosa*-driven airway inflammation^42^, but its selective impact on pathogenic Th1/17^+^ and pTh17 cells remains unexplored. In addition, despite the harmful role of IL-1β in driving neutrophilic inflammation^30^ and pathogenic IFNγ^+^Th17 cell differentiation^27^, some clinical evidence indicates increased susceptibility to severe infections following prolonged IL-1β inhibition^43^, thereby raising safety concerns about its therapeutic blockade in pwCF.

The immunophenotyping of our patient cohort revealed that Th1/17^+^ and pTh17 cell expansion was restricted exclusively to *P. aeruginosa*-positive CF lungs, implicating exposure to this pathogen as a key upstream driver. Indeed, while late *P. aeruginosa* clinical isolates showed enhanced intracellular persistence within DCs, early strains already induced high IL-1β/IL-23 secretion by DCs and efficiently triggered differentiation of IFNγ^+^Th17 subsets. Moreover, expansion of IFNγ^+^/IL-17^+^ T cells positively correlated with biofilm formation exclusively among early isolates, whereas late-adapted strains showed reduced biofilm production but still strongly drove the skewing of cTh17 into pathogenic IFNγ^+^Th17 subsets. These findings indicate that *P. aeruginosa* harbours specific virulence determinants able to promote IFNγ^+^Th17 cell generation already in the early phases of colonization, and that these immunostimulatory traits are retained throughout bacterial adaptation to the CF lungs. Although the molecular identity of these virulence determinants still remains undefined, their presence across both early and late isolates highlights them as potential therapeutic targets in preventing the harmful generation of pathogenic Th17 cells.

Notably, we establish that cTh17 cells are the primary precursors of pathogenic IFNγ^+^Th17 cells, supporting a revised view of Th17 plasticity in *P. aeruginosa* lung infections, and challenging the long-standing paradigm that adaptive immune dysfunction in CF arises only during late-stage infections. A limitation of this study is the reliance on explanted lungs, which primarily reflect advanced CF disease; whether similar Th17 reprogramming occurs at earlier or intermediate disease stages remains to be defined. Nonetheless, the combined immunological, transcriptional and TCR profiling, together with the use of clinically relevant *P. aeruginosa* strains isolated across different stages of disease, provides a rationale for targeting pathogenic IFNγ^+^Th17 cells and their upstream differentiation pathways as a therapeutic strategy.

At the molecular level, CF-derived pulmonary Th1/17^+^ and pTh17 cells show a distinct transcriptional profile characterized by increased transcriptional activation compared with their circulating counterparts—consistent with the heightened immunological pressure at the primary site of disease involvement. These upregulated gene clusters indicate that the CF lung environment not only promotes pathogenic IFNγ^+^Th17 expansion but also functionally reconfigures their transcriptional programmes. In this regard, we demonstrate that *P. aeruginosa* can directly drive the transcriptional reprogramming of cTh17 cells in vitro by promoting the upregulation of C/EBPδ and PPARγ. While PPARγ is traditionally considered a negative regulator of T cell activation^44^ and a suppressor of Th17 differentiation in specific contexts^32^, its immunomodulatory effects appear highly dependent on the inflammatory environment and immune cell types^45–47^. Similarly, our data show that *PPARG* is markedly upregulated in pathogenic pulmonary Th1/17^+^ and pTh17 subsets compared with cTh17 precursors, indicating a potential pro-inflammatory role for PPARγ. While its direct involvement in cTh17 transdifferentiation within the CF lung remains to be established, our findings highlight the striking contrast between the pro-inflammatory role of PPARγ in pathogenic IFNγ^+^Th17 cells and its anti-inflammatory function in CF airway epithelial cells^45,46^, supporting the concept that both disease context and immune cell type shape its immunomodulatory activity. Likewise, opposing roles for C/EBPδ have been described in pulmonary infections, with protective effects reported during *Klebsiella pneumoniae* infection^48^ while detrimental effects promoting tissue damage and bacterial dissemination were reported in *Streptococcus pneumoniae* models^49^. Interestingly, in the CF context, we observed selective upregulation of *CEBPD* in CF lung-resident Th1/17^+^ and pTh17 cells, as well as at the protein level in in vitro-reprogrammed cTh17 cells, supporting a causative role for *P. aeruginosa* in driving the pathogenic skewing of cTh17 cells. Moreover, despite the limited cohort size, TCR sequencing further supports this hypothesis by revealing extensive clonal expansion of private Th1/17^+^ and pTh17 clonotypes in CF lungs, indicative of persistent antigen-driven selection and tissue adaptation. Importantly, we observed a partial TCR overlap between IFNγ^+^Th17 subsets and cTh17 cells, but not Th1, reinforcing our experimental evidence that cTh17 cells are the main precursors of pathogenic Th1/17^+^ and pTh17 cells in the CF lung.

Despite these important advances, the *P. aeruginosa*- or host-derived antigens specifically driving cTh17 cell skewing remain unknown, highlighting the need for further studies to elucidate the antigenic specificity of pathogenic pulmonary Th1/17^+^ and pTh17 cells. Nevertheless, our findings reveal an immunological target with the potential to addresses a critical unmet need in CF: the selective depletion of Th1/17^+^ and pTh17 cells, or the blockade of cTh17 reprogramming, could represent a promising precision immunomodulatory strategy. Moreover, although our findings indicate that IFNγ^+^Th17 cells may act as pathogenic effectors in the CF lung, it remains to be determined whether targeting these subsets—or the upstream pathways driving their differentiation—can effectively restore mucosal immune homeostasis without compromising protective host defence. This is particularly relevant even in the era of CFTR modulators, in which inflammation remains an important clinical challenge, especially in patients with CF having only partial or no response to modulators and ongoing pulmonary bacterial colonization^12^.

In summary, our study uncovers a mechanism by which *P. aeruginosa* subverts CF mucosal immunity, reprogramming protective cTh17 cells into pathogenic Th1/17^+^ and pTh17 effectors that directly sustain epithelial damage and chronic inflammation. These findings not only identify Th1/17^+^ and pTh17 subsets as important drivers of CF immunopathology and potentially compelling targets for future therapeutic intervention, but they also lay the conceptual foundation for precision therapies that aim to restore immune homeostasis, leaving protective Th17 cells unaffected.

## Methods

### Ethics statement

The studies involving human participants were carried out in compliance with the Declaration of Helsinki protocols and were reviewed and approved by the Ethics Commission (Milano Area 2, code 15_2021). Written informed consent was obtained from all human participants included in the study.

Human bronchial epithelial cells were obtained from the facility (Servizio Colture Primarie) of the Italian Cystic Fibrosis Research Foundation (FFC Ricerca). The procedures for obtaining human bronchi from explanted lungs to establish epithelial cell cultures were approved by the appropriate Ethics Committee under registration ANTECER 042-09/07/2018. All donors signed an informed consent form that was also approved by the Ethics Committee.

### Study design

In this study, we included peripheral blood and lung samples from pwCF, whose clinical characteristics are listed in Supplementary Tables 1–4. The CF blood cohort comprised pwCF not listed for lung transplantation at enrolment; patients taking oral corticosteroids within the preceding 4 weeks, immunomodulators within the preceding 3 months or biologics within the preceding 12 weeks were excluded. The HD blood cohort consisted of volunteer participants without a personal or family history of allergic or immune-mediated diseases. A stricter exclusion criterion was specifically applied to the peripheral blood cohort to minimize potential confounding effects from concomitant inflammatory or infectious conditions and enable an unbiased comparison of circulating immune cell frequencies between patients with CF and HDs.

The lung cohorts included 22 pwCF with or without chronic lung infections (including *P. aeruginosa*, *S. aureus* or other clinically relevant intermittent pathogens) and 14 patients without CF undergoing lung transplantation for end-stage lung diseases. All patients were enrolled at the Thoracic Surgery and Lung Transplant Unit and CF center of the Fondazione IRCCS Ca’ Granda, Ospedale Maggiore Policlinico (Milan, Italy), during scheduled visits according to clinical practice.

### Growth conditions and phenotypic characterization of *P. aeruginosa* clinical strains

The *P. aeruginosa* strains used in this work, selected from a larger collection of clinical *P. aeruginosa* strains isolated from lungs of Danish pwCF^35^, are listed in Supplementary Table 5. The laboratory strain PAO1 was used as reference control. Bacteria were grown in LB medium (10 g l^−1^ tryptone, 5 g l^−1^ yeast extract powder and 5 g l^−1^ NaCl) under aerobic conditions at 37 °C overnight (O/N).

Phenotypical characterization, which included flagellar motility, pyocyanin and pyoverdine production, and biofilm formation, was performed. To evaluate flagellar motility, 3 μl of O/N bacterial cultures normalized to an optical density at 600 nm (OD_600_) = 1 was spotted in the middle of LB 0.3% soft-agar plates. The migration diameter on soft-agar plates was measured after 24 h of growth at 37 °C. To assess biofilm formation, O/N bacterial cultures were diluted to OD_600_ = 0.02 in 96-well plates and incubated for 16 h at 37 °C. Thereafter, plates were washed in ddH_2_O and stained with 1% Gram’s crystal violet solution for 20 min at RT. After three washes with ddH_2_O, adherent bacteria were dissolved in 96% ethanol and quantified at 595 nm in a microplate reader (SAFAS, MP96). The adhesion index was calculated using the equation OD_595_ (crystal violet)/OD_600_ (planktonic culture). Pyocyanin production was quantified with the chloroform–HCl extraction assay: 1 ml of O/N bacterial cultures was centrifuged and, after evaluation of OD_600_, was centrifugated, and the supernatant was mixed with 1 ml of chloroform. After centrifugation for 5 min at maximum speed, the bottom layer was mixed with an equal volume of HCl 0.2 N, thoroughly vortexed and centrifuged at maximum speed for 5 min. The absorbance of the up layer was measured at 520 nm in a microplate reader. Pyocyanin concentration was calculated using the equation (OD_520_/OD_600_) × 17.072. For pyoverdine production, 1 ml of O/N bacterial cultures was centrifuged and, after evaluation of OD_600_, was centrifugated, and 100 μl of supernatant was mixed with 900 μl of Tris–HCl (pH 7.5). The solution absorbance was then acquired at 405 nm. Pyoverdine production was calculated using the equation (OD_405_/OD_600_) × 16.500.

### Isolation of primary human dendritic cells and CD4^+^ T_H_-cell subsets

Human monocytes were purified using CD14 positive selection (CD14 MicroBeads, Miltenyi Biotec) from heparinized blood samples derived from HDs and patients with CF after density gradient separation by Lympholyte. moDCs were generated using 50 ng ml^−1^ of recombinant human granulocyte–monocyte colony stimulating factor and 20 ng ml^−1^ of human IL-4 for 7 days (ref. ^50^).

Pulmonary T_H_cell subsets were isolated from explanted lung tissues, which were processed rapidly after transplantation in calcium- and magnesium-free Hanks’ balanced salt solution (HBSS, Euroclone), supplemented with 1% penicillin–streptomycin and 300 µg ml^−1^ gentamycin (HBSS-P/S/G). Tissues were incubated for 15 min in HBSS containing dithiothreitol (0.16 mg ml^−1^, Sigma-Aldrich) to remove viscous mucus and pulmonary bacteria and, after an extensive wash, incubated for an additional 60 min at 37 °C in HBSS-P/S/G supplemented with 1 mM EDTA (Sigma). Tissues were subsequently digested for 1–3 h at 37 °C in RPMI 1640 medium containing 1 mg ml^−1^ Collagenase D (Roche) in a 5% CO_2_ incubator. Pulmonary mononuclear cells released from lung tissues were filtered through a 70-μm cell strainer and washed with complete RPMI 1640 medium supplemented with 10% FBS, before cell sorting.

Th1 and Th17 cell subsets were sort-purified (FACSAria III, BD Biosciences, or Aurora CS, Cytek) from peripheral blood of HDs and patients with CF, or from lung tissue of patients with or without CF, according to the expression of a specific surface marker combination among CD4^+^IL-7R^+^CD25^low^ cells (Extended Data Fig. 1a):

- CCR6^−^CXCR3^+^ (Th1 cells)
- CCR6^+^CXCR3^−^CCR5^−^ (conventional Th17 cells; cTh17)
- CCR6^+^CXCR3^−^CCR5^+^ (pro-inflammatory Th17 cells; pTh17)
- CCR6^+^CXCR3^+^CCR5^−^ (Th1/17^−^ cells)
- CCR6^+^CXCR3^+^CCR5^+^ (Th1/17^+^ cells)

### *P. aeruginosa* infection assays of moDCs

HD-derived moDCs were seeded at 2 × 10^5^ cells per well in complete RPMI without antibiotics and infected with 4 × 10^6^ colony forming units (CFU) ml^−1^ of each *P. aeruginosa* strain (multiplicity of infection 1:20). After 90 min of infection, 300 µg ml^−1^ of gentamycin was added for 1 h to kill extracellular bacteria. To assess bacterial uptake (phagocytosis), moDCs were washed with PBS and lysed with 1% Triton X-100 (Sigma-Aldrich), and intracellular CFU were quantified by plating serial dilutions on LB agar. For intracellular persistence, following the initial gentamicin treatment, the culture medium was replaced with complete RPMI medium containing gentamicin (200 µg ml^−1^) to prevent extracellular bacterial regrowth. After 24 h of infection, moDCs were washed and lysed, and intracellular CFU were quantified as described above. At 24 h postinfection, supernatants of infected cells were collected and stored at −20 °C for the subsequent quantification of cytokine and chemokine concentration by enzyme-linked immunosorbent assay (ELISA).

To further support *P. aeruginosa* intracellular persistence and moDC viability, Giemsa staining was performed on cytospinned moDCs after 24 h of infection. In parallel, moDC viability was also quantified using the WST-1 assay (Roche), according to the manufacturer’s instructions. Results were expressed as percentages of viable infected moDCs compared with non-infected moDCs taken as 100%.

### Immunophenotypic analysis and inflammatory response of *P. aeruginosa*-infected moDCs

After 24 h of infection with *P. aeruginosa* clinical strains, as described above, the activation state of human moDCs was assessed by evaluating the expression of a panel of surface markers. Briefly, *P. aeruginosa*-infected moDCs were collected, washed with PBS–2% FBS and stained for 15 min at 37 °C with a combination of monoclonal antibodies against CD40, CD80, CD83, CD86, CCR7, CD206 (mannose receptor), CD209 (DC-SIGN), CD172α (SIRPα), CD274 (PD-L1) and CD366 (TIM-3) (all from BioLegend). Data acquisition was performed using a Northern Lights cytometer (Cytek), and data were analysed using FlowJo software.

The inflammatory response of *P. aeruginosa*-infected moDCs was assessed by quantifying cytokine and chemokine levels in culture supernatants collected at 24 h postinfection. Concentrations of IL-23 (Thermo Fisher), IL-1β, IL-12 (BioLegend), IP-10, CCL20 (MIP-3α) and RANTES (CCL5) (R&D Systems) were measured by ELISA according to the manufacturers’ instructions. The ELISA plates were read on a microplate reader (SAFAS MP96), and data were analysed with Prism software (v.10, GraphPad Software).

### ALI Bronchial epithelial cell model and co-culture with Th17 cell subsets

Human bronchial epithelial cells from individuals with no CF were obtained by ‘Servizio Colture Primarie’ of FFC Ricerca. Bronchial cell isolation, expansion and differentiation were performed as previously described^51,52^. The ALI conditions were obtained by plating bronchial epithelial cells at high density on Transwell porous inserts (cc3470, Corning Costar). After 24 h, the medium on the basolateral side was replaced with the differentiative medium PneumaCult ALI (Stemcell Technology), whereas the medium on the apical side was removed to obtain the ALI condition. This condition allowed the formation of a tight epithelium with full mucociliary differentiation in 2–3 weeks (ref. ^52^).

For co-culture experiments, bronchial epithelial cells were exposed basolaterally to PneumaCult ALI medium alone (control) or to ex vivo-activated Th17 cell subsets, including cTh17, Th1/17^+^ or pTh17 cells. Briefly, 300,000 sort-purified cTh17, Th1/17^+^ or pTh17 cells were isolated from the peripheral blood of patients with CF and stimulated with phorbol 12-myristate 13-acetate (PMA)–ionomycin for 4 h. Cells were then washed, resuspended in PneumaCult ALI medium and added to the lower chamber of Transwell inserts. After 24 h, basolateral supernatants were collected for cytokine and chemokine quantification by ELISA, and epithelial cells were fixed for immunofluorescence analysis.

The amount of IL-8, IL-6 (ImmunoTools), TNF (eBioscience), IP-10, CCL20 (MIP-3α) and RANTES (CCL5) (R&D Systems) in the basolateral medium of human bronchial epithelial cells was quantified by ELISA, according to manufacturer’s protocol. The ELISA plates were read on a microplate reader (SAFAS MP96), and data were analysed with Prism software (v.10, GraphPad Software).

### Immunofluorescence analysis

After 24 h of co-culture, Transwell membranes containing bronchial epithelial cells, either untreated (CTR) or co-cultured with Th17 cell subsets, were fixed in 10% neutral buffered formalin and then washed with PBS 3 times. After antigen retrieval with 10 mM citrate buffer, samples were permeabilized with 0.3% Triton X-100 in PBS for 5 min, blocked with 1% BSA in PBS for 2 h and then incubated overnight at 4 °C with primary anti-tight junction protein ZO-1 (Thermo Scientific, 1:500) and anti-acetylated tubulin antibodies (Sigma-Aldrich, 1:300). After three washes with PBS, cells were incubated with appropriate combinations of secondary fluorescent antibodies. After 1 h, cells were washed in PBS and slides were sealed with Fluoroshield mounting medium with DAPI (Sigma-Aldrich) for nuclear counterstaining. Images were acquired by a Nikon Yokogawa CSU W1 spinning disk confocal microscope using a ×60 oil immersion objective (NA 1.4). The Z-stacks were acquired by taking an image every 0.3 μm. For quantitative analysis of ZO-1 staining, maximum projections of the image stacks were generated, and segmentation masks were created using the AI-assisted Draw Object Tool of Arivis Pro v.4.4 software (Carl Zeiss Microscopy), which automatically detects structures within selected regions. Both the total segmented area and the relative volume normalized to the total fields were calculated, providing a quantitative measure of junctional discontinuity, epithelial structural integrity and spatial representation in the 2D projection.

### Transdifferentiation of IFNγ^+^Th17 cells

An antigen-specificity assay was performed to assess the transdifferentiation of cTh17 or Th1 cells into IFNγ-producing Th17 cells following stimulation with *P. aeruginosa*-infected moDCs. Briefly, HD-derived moDCs were seeded at 1 × 10^4^ cells per well into 96-well round-bottom plates and infected with *P. aeruginosa* strains (multiplicity of infection 1:20). After 90 min, extracellular bacteria were killed by adding 300 μg ml^−1^ of gentamicin, and after washing, infected moDCs were maintained in RPMI supplemented with 200 μg ml^−1^ of gentamycin. Autologous cTh17 and Th1 cells were sort purified from peripheral blood of HDs and co-cultured with infected moDCs at a 5:1 ratio in RPMI medium supplemented with 200 μg ml^−1^ gentamycin and 20 U ml^−1^ of recombinant human IL-2. Complete medium was replaced every 3 days. After 10 days, cTh17 and Th1 cells were analysed for intracellular cytokine expression after a further polyclonal stimulation with 50 ng ml^−1^ PMA and 1 µg ml^−1^ ionomycin for 2 h. Subsequently, brefeldin A (10 µg ml^−1^) was added for an additional 5–6 h, and cells were then fixed with 2% paraformaldehyde solution. After permeabilization with PBS–0.5% saponin, cells were stained with anti-CD40L (Biolegend), anti-IL-17A (Biolegend) and anti-IFNγ (Biolegend). Background activation of cTh17 or Th1 cells stimulated with autologous uninfected moDCs was subtracted to calculate antigen-dependent cell transdifferentiation.

To evaluate the upregulation of the transcription factors PPARγ, C/EBPδ and BATF3 among cTh17 cells expressing IL-17 and/or IFNγ, the same antigen-specificity assay was repeated with CF-derived moDCs and autologous cTh17 cells. After 10 days of co-culture, cells were restimulated as described above and stained intracellularly using the FOXP3 Fix/Perm kit (eBioscience) according to the manufacturer’s protocol. In addition to anti-CD40L, anti-IL-17A and anti-IFNγ, antibodies against PPARγ (Novusbio), C/EBPδ (Novusbio) and BATF3 (Novusbio) were included in the staining panel to assess their expression within cTh17 cells co-expressing IL-17 and IFNγ (IL-17^+^IFNγ^+^), expressing either IL-17 or IFNγ alone (IL-17^+^IFNγ^−^, IL-17^−^IFNγ^+^), or lacking both cytokines (IL-17^−^IFNγ^−^, double-negative). Acquisition was performed with FACSCanto II (BD Biosciences) or NorthernLight cytometer (Cytek) and data were analysed using FlowJo software.

### RNA sequencing

mRNA sequencing was performed on ex vivo-isolated cTh17, Th1/17^+^, pTh17 and Th1 cells, sort purified from peripheral blood (*n* = 3), lung tissue of patients with CF (*n* = 4) and lung tissue of patients without CF (*n* = 4), except for pTh17 cells, which were not included owing to their extremely low frequency in this patient group. Total RNAs were extracted using the kit Quick-RNA MicroPrep (Zymo Research), the quality of RNAs was evaluated using TapeStation 4200 (Agilent) and only RNAs showing an RNA integrity number > 7 were used for library preparation. Sequencing libraries were prepared using the SMART Seq v4 PLUS kit (Takara) starting from 5 ng total RNA per sample and sequenced with the NovaSeq6000 (Illumina), generating on average 57.6 million 100-bp paired-end reads per sample. Paired-end reads were aligned to the ‘analysis-set’ GRCh38.p14 reference assembly of the human genome using STAR^53^, and gene expression levels were quantified by RSEM^54^ on the RefSeq annotation of the GRCh38 reference assembly of the human genome^55^. Reads counts were batch corrected for donors using Combat-seq^56^. Differential gene expression analyses were performed by the exact test for a difference in mean between two groups of negative binomial random variables as implemented by the exactTest function in edgeR^57^. *P* values were corrected using the Benjamini–Hochberg procedure for the control of the false discovery rate (FDR)^58^. Only genes with an FDR < 0.05 and an absolute fold change of expression of at least two (|log_2_(fold change)| > 1) were considered differentially expressed (DEGs).

The following comparisons were performed:

1. Pathogenic (pTh17 and Th1/17^+^) versus non-pathogenic (cTh17 and Th1) cell subsets in CF lungs
2. Pathogenic (pTh17 and Th1/17^+^) versus non-pathogenic (cTh17 and Th1) cell subsets in CF blood
3. Pathogenic Th1/17^+^ versus non-pathogenic (cTh17 and Th1) cell subset in non-CF lungs

To define a robust transcriptional signature specifically associated with pathogenic T cell subsets in CF lungs, we selected only those genes that were differentially expressed in comparison (1), but not in comparison (2) or (3). In addition, only genes showing a consistent direction of expression change (up- or downregulated) in at least three out of four donors were considered.

To summarize pathogenic T cell subset signatures across CF compartment-specific transcriptional modules, a mean *z*-score was computed on lung or blood DEGs derived from the above-described comparisons (1) and (2). The following formula was applied to compute a single DEG *z*-score:

$$z=(x-\mu )/\sigma$$

where *x* is the expression value of a single DEG in one sample and *μ* and *σ* are the mean expression and standard deviation, respectively, of the same DEG across all samples.

Functional enrichment analyses for the identification of underlying molecular pathways were executed by GSEA^59^ v.4.4.0. Genes were ranked for GSEA by a signed log_2_(fold change) weighted by −log_10_(FDR) (log(FDR)) to incorporate both effect size and statistical significance.

### TCR–RNA sequencing

TCR sequencing was performed on the same T cell subsets ex vivo purified and analysed for RNA sequencing. RNA libraries were constructed using the Takara SMARTer Human TCR α/β Profiling Kit with integrated unique molecular identifiers to facilitate precise quantification of clonotypes. Raw FASTQ files underwent processing with MiXCR (v.4.7.0)^60^ using the analyse takara-human-rna-tcr-umi-smarter-v2 pipeline, which was specifically optimized for this kit. Clonotypes were assembled based on CDR3 sequences using the --assemble-clonotypes-by CDR3 parameter and the human genome (--species hsa). Only productive CDR3 nucleotide sequences were preserved for subsequent analytical processes. The counts of functional clonotypes per sample showed a range from 4,651 to 84,355. Repertoire diversity, clonal expansion and clonotype distributions were examined individually for TCR α and β chains using the Immunarch R package (v.1.0.0)^61^. The overlap among repertoires was quantitatively assessed using VDJtools (v.1.2.1)^62^. To focus on public responses, we excluded private clonotypes, defined as productive CDR3 amino acid sequences detected in exactly one patient and absent from all others. Operationally, we built a Boolean presence–absence matrix of CDR3 amino acid sequences across patients and discarded sequences fulfilling the criterion (present in one column; sum across all other columns = 0), thereby retaining only clonotypes shared by at least two individuals. Custom visual representations, including scatter plots depicting clonotype frequencies, were generated in Python using the Matplotlib package (v.3.10.0).

### Quantification and statistical analysis

Statistical significance was assessed through unpaired two-sided *t*-test for comparison of two groups (applying Welch’s correction where needed) or a two-sided Mann–Whitney test for non-normally distributed data. For comparison of more than two groups, statistical significance was assessed through one-way ANOVA (with Dunn’s multiple comparisons for non-normally distributed variables). Correlation analyses between continuous variables were performed using Pearson’s correlation coefficient.

All experiments were independently repeated at least three times. The exact number of independent experiments performed for each analysis is specified in the corresponding figure legends.

Statistics were performed with Prism software (v.9; GraphPad Software), and specific details for each analysis are reported in the respective figure legends.

### Reporting summary

Further information on research design is available in the Nature Portfolio Reporting Summary linked to this article.

## Supplementary information

Supplementary Information Supplementary Figs. 1 and 2 and Supplementary Tables 1–5.

Reporting Summary

Peer Review file

Supplementary Data Source data for Supplementary Fig. 1 (sheets 1 and 2): clinical characteristics of patient cohorts and correlation analysis between age, pulmonary function (FEV1pp) and CD4^+^ T cell subsets. Source data for Supplementary Fig. 2 (sheets 3 and 4): correlation analysis between virulence traits of clinical *P. aeruginosa* isolates and phenotypic responses of human moDCs. Source data for Supplementary Table 1 (sheet 5): subject cohorts enrolled in this study. Source data for Supplementary Table 2 (sheet 6): characteristics of the CF lung cohort. Source data for Supplementary Table 3 (sheet 7): characteristics of the non-CF lung cohort. Source data for Supplementary Table 4 (sheet 8): characteristics of the CF blood cohort. Source data for Supplementary Table 5 (sheet 9): characteristics of clonal *P. aeruginosa* clinical strains used in this study. Supplementary Data 1 (sheet 10): differential gene expression in CF blood T cell subsets. Differentially expressed genes (FDR < 0.01) identified in blood-derived T cell subsets from cystic fibrosis donors, comparing protective (cTh17 and Th1) and pathogenic (pTh17 and Th1/17^+^) populations. Expression values are reported as log(CPM) for each sample. Sample identifiers comprise a pseudonymized donor ID, tissue origin (B, blood) and cell subset. For each gene, log fold change (log(FC)), *P* value and FDR are provided. log(FC) is calculated as pathogenic versus protective subsets. Supplementary Data 2 (sheet 11): differential gene expression in CF lung T cell subsets. Differentially expressed genes (FDR < 0.01) identified in lung-derived T cell subsets from cystic fibrosis donors, comparing protective (cTh17 and Th1) and pathogenic (pTh17 and T_H_1/17+) populations. Expression values are reported as log(CPM) for each sample. Sample identifiers comprise a pseudonymized donor ID, tissue origin (L, lung) and cell subset. For each gene, log(FC), *P* value and FDR are provided. log(FC) is calculated as pathogenic versus protective subsets.

## Source data

Source Data Fig. 1 Statistical source data.

Source Data Fig. 2 Statistical source data.

Source Data Fig. 3 Statistical source data.

Source Data Fig. 4 Statistical source data.

Source Data Fig. 5 Statistical source data.

Source Data Extended Data Fig. 1 Statistical source data.

Source Data Extended Data Fig. 2 Statistical source data.

Source Data Extended Data Fig. 3 Statistical source data.

Source Data Extended Data Fig. 4 Statistical source data.

Source Data Extended Data Fig. 5 Statistical source data.

Source Data Extended Data Fig. 6 Statistical source data.

Source Data Extended Data Fig. 7 Statistical source data.

Source Data Extended Data Fig. 8 Statistical source data.

Source Data Extended Data Fig. 9 Statistical source data.

## Data Availability

The transcriptomic and TCR-sequencing data are available in the ArrayExpress database (https://www.ebi.ac.uk/biostudies/arrayexpress) under accession codes E-MTAB-15774 for RNA sequencing and E-MTAB-15769 for TCR–RNA sequencing. The processed RNA- and TCR-sequencing data are available in the Supplementary Information. Source data are provided with this paper.
