# Supplementary material for: Pseudomonas aeruginosa induces the generation of pathogenic IFNγ+Th17 cells that promote lung damage and chronic inflammation in cystic fibrosis
Source: Nat Microbiol. 2026 Sep 24;11(11):3208–23. doi: 10.1038/s41564-026-02469-2 (PMC13739742; doi:10.1038/s41564-026-02469-2)
Supplement: Supplementary file 1 [file 41564_2026_2469_MOESM1_ESM.pdf]

nature microbiology

Supplementary information

https://doi.org/10.1038/s41564-026-02469-2

Pseudomonas aeruginosa induces the generation of pathogenic IFNγ$^+$Th17 cells that promote lung damage and chronic inflammation in cystic fibrosis

In the format provided by the authors and unedited

TABLE-OF-CONTENTS:

- **Supplementary Figure 1:** Clinical characteristics of patient cohorts and correlation analysis between age, pulmonary function (FEV_1pp), and $CD4^+$ T cell subsets.
- **Supplementary Figure 2:** Correlation analysis between virulence-traits of clinical *P. aeruginosa* isolates and phenotypic responses of human moDCs.
- **Supplementary Table 1:** Subject cohorts enrolled in this study.
- **Supplementary Table 2:** Characteristic of CF Lung cohort.
- **Supplementary Table 3:** Characteristic of non-CF Lung cohort.
- **Supplementary Table 4:** Characteristic of CF Blood cohort.
- **Supplementary Table 5:** Characteristics of clonal *P. aeruginosa* clinical strains used in this study.

Supplementary Figure 1

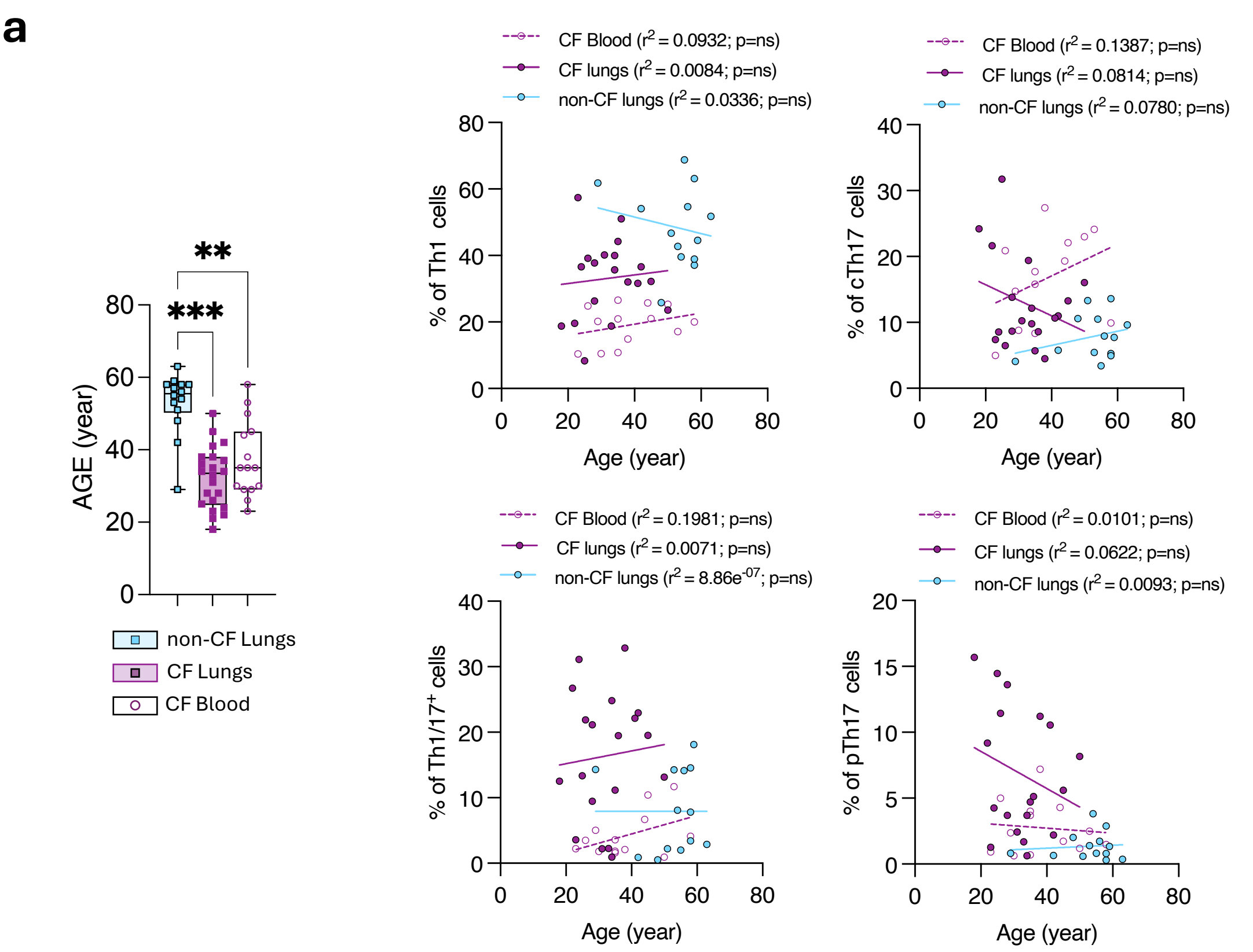

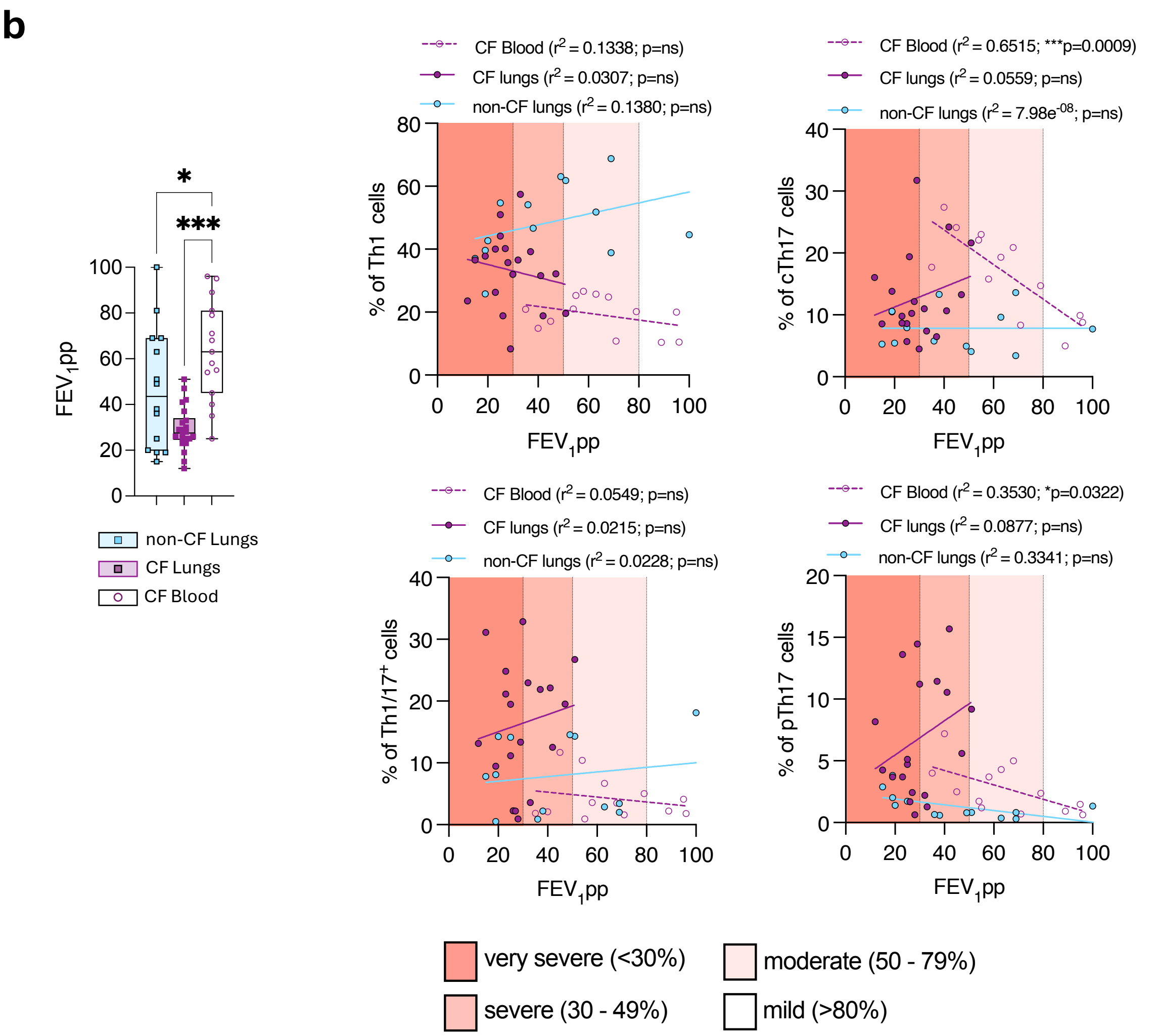

Supplementary Figure 2

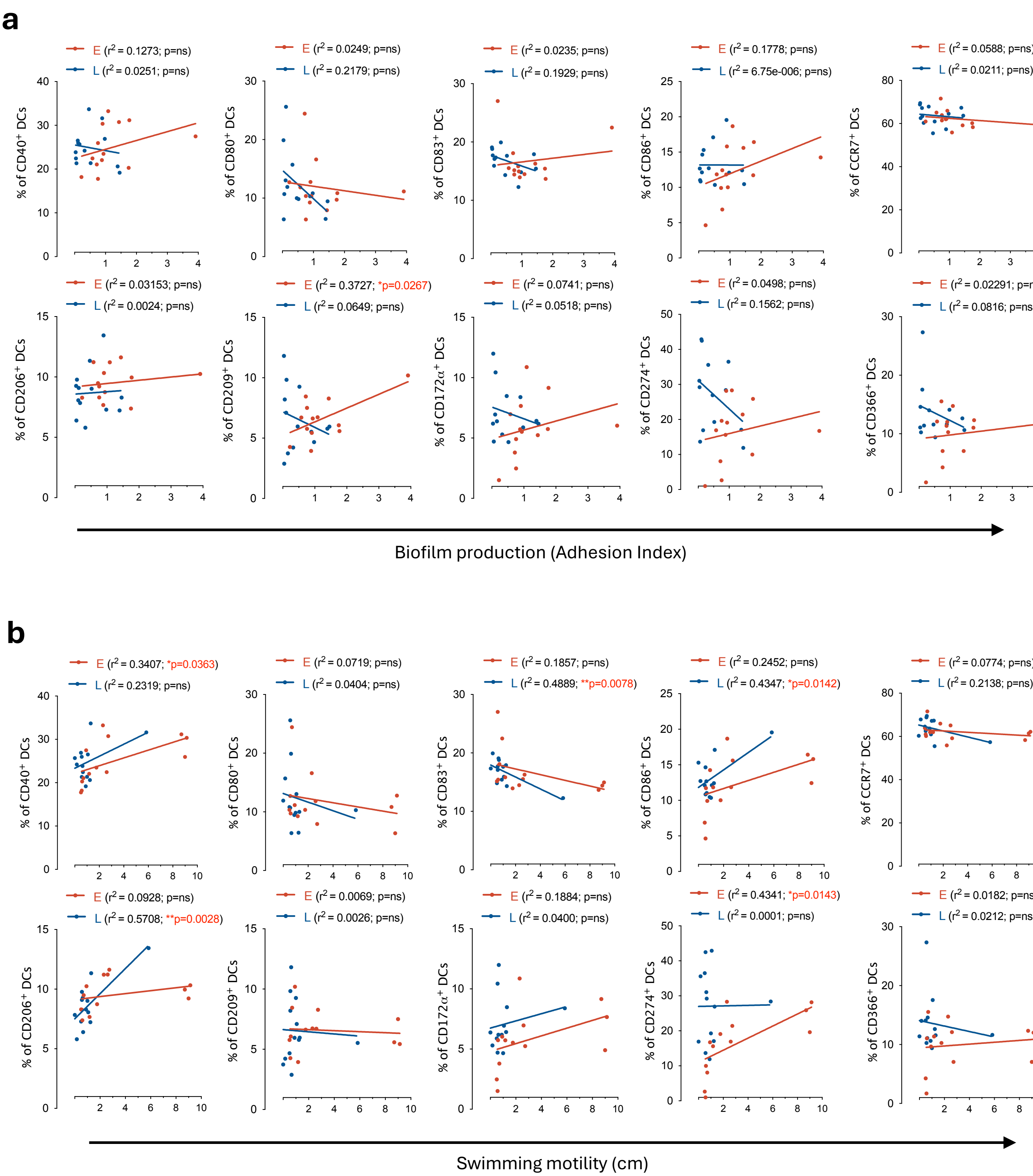

SUPPLEMENTARY FIGURE 1: Clinical characteristics of patient cohorts and correlation analysis between age, pulmonary function (FEV_1pp), and $CD4^+$ T cell subsets.

Age (**a**) and Forced expiratory Volume in 1 second percent predicted (FEV_1pp, **b**) distribution among the analysed cohorts: CF peripheral blood (n=15), CF lungs (n= 22), and non-CF lungs (n=14). Data are presented as Data are presented as box-and-whisker plots showing the median (center line) and minimum-to-maximum range (whiskers) and individual data points.
Statistical significance was assessed using one-way ANOVA and reported as *p < 0.05, ** p < 0.01, *** p < 0.001.
Correlation analysis between age (**a**) or FEV_1pp (**b**) and the percentage of the indicated T cell subsets across CF blood, CF lungs, and non-CF lungs. In panel **b**, background colour bands indicate ranges of FEV_1pp corresponding to distinct levels of lung disease severity in CF, from mild to very severe, as defined in clinical practice. Positive or negative correlations between variables were evaluated using Pearson correlation analysis. R^2 together with p values are indicated within each graph.

SUPPLEMENTARY FIGURE 2: Correlation analysis between virulence-traits of clinical *P. aeruginosa* isolates and phenotypic responses of human moDCs.

Correlation analysis between biofilm production (**a**), or swimming motility (**b**), of clonal *P. aeruginosa* clinical strains (early vs late) and surface markers expression in HD-derived moDCs at 24h post-infection. Positive or negative correlations between variables were determined using Pearson correlation analysis. R squares and p values for each correlation were reported in the graphs.

Supplementary Table 1: Subject cohorts enrolled in this study

CLINICAL CHARACTERISTICS	CF LUNG (n=22)	non-CF LUNG (n=14)	CF BLOOD (n=15)
Male/Female (n)	10/12	11/3	9/6
Age (mean ± SEM)	32.2 ± 1.79	52.9 ± 2.30	37.3 ± 2.70
LAS[1] (mean ± SEM)	41.9 ± 1.68	41.8 ± 1.98	-
FEV_1pp[2] (mean ± SEM)	29.3 ± 2.05	46.7 ± 7.12	63.6 ± 5.65
**Microbial colonization**[3]			
- Chronic[4] *Pseudomonas aeruginosa* (n)	17	0	11
- Chronic[4] *Staphylococcus aureus* (n)	10	0	7
- *Other intermittent pathogens* (n)	14	0	4
**Therapies**			
- CFTR Modulators (n)	5	0	3
- Inhaled antibiotics (n)	6	0	8
- Azithromycin (n)	15	1	10

[1] Lung Allocation Score (**LAS**)
[2] Forced Expiratory Volume in 1 second percent predicted (**FEV_1pp**)
[3] Lung colonization may include more than one pathogen per patient. Detailed microbiological profiles for individual patients are provided in Supplementary Tables 3–5.
[4] Chronic infection is defined, according to the European CF Society Patient Registry (ECFSPR), when >50% of respiratory samples collected during the last 12 months preceding the last reported culture are positive for the pathogen under consideration.

Supplementary Table 2: Characteristic of CF Lung cohort.

pwCF	CLINICAL CHARACTERISTICS				THERAPIES				PULMONARY INFECTIONS		
	CFTR variants[1]	FEV_1pp[2]	LAS[3]	Exacerbation Frequency[4]	Comorbidities[5]	CFTR modulators[6]	Inhaled Antibiotics[7]	AZM[8]	Chronic *P. aeruginosa*[9]	Chronic *S. aureus*[9]	Other intermittent pathogens[10]
**CF1_L**	F/F	26	33.5	≥ 3	PI	LUM/IVA	-	No	-	-	Ax, Sm, Af
**CF2_L**	MF/MF	15	52.65	≥ 3	PI	-	TOB	Yes	+	-	Ax
**CF3_L**	F/F	32	33.25	0–2	PI	LUM/IVA	-	Yes	+	-	Ax
**CF4_L**	F/MF	29	45.52	≥ 3	CFRD, PI, GERD	-	PIP/TAZ	Yes	-	MRSA	MAC
**CF5_L**	F/MF	29	45.42	≥ 3	CFRD, PI	-	COL	Yes	+	MRSA	-
**CF6_L**	MF/MF	25	38.40	≥ 3	PI	-	-	No	+	-	-
**CF7_L**	MF/MF	30	34.64	≥ 3	CFRD, PI, AH	-	-	Yes	+	MSSA	Af
**CF8_L**	Unknown	23	40.80	0–2	CFRD, PI	-	-	Yes	+	MRSA	-
**CF9_L**	F/F	28	37.14	≥ 3	CFRD, PI, GERD	-	-	No	-	MRSA	Ax
**CF10_L**	F/MF	19	47.56	0–2	CFRD, PI	-	AZT, LVX	Yes	+	-	-
**CF11_L**	F/F	33	45.71	≥ 3	PI, GERD	LUM/IVA	-	Yes	-	-	Ax
**CF12_L**	F/MF	51	45.83	≥ 3	PI, GERD	-	-	Yes	+	MSSA	Sm
**CF13_L**	F/MF	37	54.7	≥ 3	PI, GERD	-	TOB	No	+	-	-
**CF14_L**	MF/MF	26	36.6	≥ 3	CFRD, PI	-	-	Yes	+	-	-
**CF15_L**	F/MF	27	37.12	0–2	CFRD, PI, GERD	-	-	Yes	-	MSSA	Ps, Ax
**CF16_L**	F/MF	25	45.80	≥ 3	CFRD, PI	-	-	Yes	+	-	-
**CF17_L**	Unknown	12	36.92	≥ 3	PI	IVA	-	Yes	+	MRSA	Sm
**CF18_L**	MF/MF	25	37.84	≥ 3	PI	-	-	Yes	+	MRSA	Bm
**CF19_L**	F/MF	42	33.44	0–2	PI	ETI	-	No	+	MSSA	MAC
**CF20_L**	MF/MF	23	63.73	0–2	PI	-	-	No	+	-	-
**CF21_L**	F/F	41	40.98	0–2	PI	-	TOB	Yes	+	-	Ax, Sm
**CF22_L**	MF/RF	47	34.99	≥ 3	PI, GERD	-	-	No	+	-	Ax, MAC

[1]CFTR variants are classified according to the functional defect in **F** = F508del, **MF** = minimal function (Variants in classes I and II), **RF** = residual function (variants in classes IV, V and VI)

[2] Forced Expiratory Volume in 1 second percent predicted (**FEV_1pp**)

[3] Lung Allocation Score (**LAS**)

[4] Number of Exacerbation per year

[5] Pancreatic insufficiency (**PI**), Cystic fibrosis-related diabetes (**CFRD**), Gastroesophageal reflux disease (**GERD**), Arterial hypertension (**AH**)

[6] CFTR modulators: lumacaftor/ivacaftor (**LUM/IVA**), Elexacaftor/Tezacaftor/Ivacaftor (**ETI**)

[7] Tobramycin (**TOB**), Piperacillin-tazobactam (**PIP/TAZ**), Colistin (**COL**), aztreonam (**AZT**), levofloxacin (**LVX**)

[8] Azithromycin (**AZM**)

[9] Chronic infection is defined, according to the European CF Society Patient Registry (ECFSPR), when >50% of respiratory samples collected during the last 12 months preceding the last reported culture are positive for the pathogen under consideration. Methicillin-sensitive *Staphylococcus aureus* (**MSSA**); methicillin-resistant *Staphylococcus aureus* (**MRSA**)

[10] *Achromobacter xylosoxidans* (**Ax**); *Strenotrophomonas maltophilia* (**Sm**)*; Mycobacterium avium complex* (**MAC**); *Pandorea sputorum* (**Ps**); *Burkholderia multivoran* (**Bm**)*; Aspergillus fumigatus* (**Af**).

Supplementary Table 3: Characteristic of non-CF Lung cohort.

non-CF	CLINICAL CHARACTERISTICS				THERAPIES			PULMONARY INFECTIONS		
	Pathology[1]	FEV_1pp[2]	LAS[3]	Exacerbation Frequency[4]	Comorbidities[5]	Inhaled Antibiotics	AZM[6]	Chronic *P. aeruginosa*	Chronic *S. aureus*	Other intermittent pathogens
**PT1_L**	COPD	81	42.38	0–2	CAD	No	No	-	-	-
**PT2_L**	ILD	38	49.74	0–2	-	No	No	-	-	-
**PT3_L**	ILD	100	37.54	0–2	Obesity, GERD, AH	No	No	-	-	-
**PT4_L**	COPD	20	35.82	0–2	AH	No	No	-	-	-
**PT5_L**	COPD	15	36.87	0–2	GERD, AH	No	No	-	-	-
**PT6_L**	PPFE	19	45.42	0–2	GERD	No	No	-	-	-
**PT7_L**	ILD	69	35.27	0–2	GERD	No	No	-	-	-
**PT8_L**	COPD	19	31.88	≥ 3	-	No	No	-	-	-
**PT9_L**	ILD	25	36.85	0–2	-	No	No	-	-	-
**PT10_L**	ILD	63	35.83	0–2	DM	No	No	-	-	-
**PT11_L**	ILD	36	55.21	0–2	SSc, GERD	No	No	-	-	-
**PT12_L**	ILD	51	54.52	≥ 3	-	No	Yes	-	-	-
**PT13_L**	ILD	69	41.92	0–2	-	No	No	-	-	-
**PT14_L**	ILD	49	45.5	0–2	Obesity, AH	No	No	-	-	-

[1] Chronic obstructive pulmonary disease (**COPD**); Interstitial lung disease (**ILD**); Pleuroparenchymal fibroelastosis (**PPFE**)
[2] Forced Expiratory Volume in 1 second percent predicted (**FEV_1pp**)
[3] Lung Allocation Score (**LAS**)
[4] Number of Exacerbation per year
[5] Coronary artery disease (**CAD**); Gastroesophageal reflux disease (**GERD**), Diabetes mellitus (**DM**); Systemic sclerosis (**SSc**); Arterial hypertension (**AH**)
[6] Azithromycin (**AZM**)

Supplementary Table 4: Characteristics of CF Blood cohort.

pwCF	CLINICAL CHARACTERISTICS			THERAPIES				PULMONARY INFECTIONS		
	CFTR variants[1]	FEV_1pp[2]	Exacerbation Frequency[3]	Comorbidities[4]	CFTR modulators[5]	Inhaled Antibiotics[6]	AZM[7]	Chronic *P. aeruginosa*[8]	Chronic *S. aureus*[8]	Other intermittent pathogens[9]
**CF1_B**	F/MF	58	≥ 3	CFRD, PI	No	COL	Yes	+	-	Af
**CF2_B**	F/MF	68	≥ 3	CFRD, PI	No	-	Yes	+	-	-
**CF3_B**	F/MF	35	0–2	PI	No	-	Yes	-	MRSA	Ax
**CF4_B**	F/MF	55	≥ 3	CFRD, PI	No	-	Yes	+	-	Af
**CF5_B**	F/MF	40	≥ 3	CFRD, PI	No	COL	Yes	+	MRSA	-
**CF6_B**	MF/RF	63	0–2	-	No	COL	Yes	+	MSSA	-
**CF7_B**	MF/MF	25	≥ 3	PI	No	LVX	No	+	-	-
**CF8_B**	F/MF	54	≥ 3	CFRD, PI, CFLD	No	COL	No	+	-	-
**CF9_B**	F/MF	45	≥ 3	CFRD	No	COL	Yes	+	-	-
**CF10_B**	F/RF	79	0–2	PI	No	-	No	-	MRSA	-
**CF11_B**	F/RF	95	0–2	-	ETI	COL	Yes	+	MSSA	-
**CF12_B**	F/RF	81	0–2	-	ETI	COL	Yes	+	-	MABSC
**CF13_B**	F/RF	96	0–2	-	No	-	No	-	MSSA	-
**CF14_B**	MF/MF	71	≥ 3	CFRD	No	-	Yes	+	MSSA	-
**CF15_B**	F/F	89	0–2	PI	ETI	-	No	-	-	-

[1]CFTR variants are classified according to the functional defect in **F** = F508del, **MF** = minimal function (Variants in classes **I** and **II**), **RF** = residual function (variants in classes IV, V and VI)
[2] Forced Expiratory Volume in 1 second percent predicted (**FEV_1pp**)
[3] Number of Exacerbation per year
[4] Cystic fibrosis-related diabetes (**CFRD**), Pancreatic insufficiency (**PI**), Cystic Fibrosis Liver Disease (**CFLD**)
[5] CFTR modulators: Elexacaftor/Tezacaftor/Ivacaftor (**ETI**)
[6] Colistin (**COL**), levofloxacin (**LVX**)
[7] Azithromycin (**AZM**)
[8] Chronic infection is defined, according to the European CF Society Patient Registry (ECFSPR), when >50% of respiratory samples collected during the last 12 months preceding the last reported culture are positive for the pathogen under consideration. Methicillin-sensitive *Staphylococcus aureus* (**MSSA**); methicillin-resistant *Staphylococcus aureus* (**MRSA**)
[9] *Aspergillus fumigatus* (**Af**); *Achromobacter xylosoxidans* (**Ax**); *Mycobacterium abscessum complex* (**MABSC**)

Supplementary Table 5: Characteristics of clonal *P. aeruginosa* clinical strains used in this study.

CF Patient	*P. aeruginosa* strain	Year of isolation (Persistence in years)	Clone Type
–	PAO1#	–	–
Pa01	S47 (E)	1984 (0)	**DK01**
	S41 (L)	2012 (28)	
Pa02	S44 (E)	1984 (0)	
	S45 (L)*	2016 (32)	
Pa03	S27 (E)	1984 (0)	**DK02**
	S28 (L)	2017 (33)	
Pa04	S31 (E)	1991 (0)	
	S32 (L)	2015 (24)	
Pa05	S26 (E)	1973 (0)	
	S9 (L)	2008 (35)	
Pa06	S42 (E)	2006 (0)	**DK06**
	S38 (L)	2021 (15)	
Pa07	S36 (E)	2004 (0)	
	S37 (L)*	2021 (17)	
Pa08	S43 (E)	2006 (0)	**DK19**
	S33 (L)	2021 (15)	
Pa09	S34 (E)	2006 (0)	
	S35 (L)	2021 (15)	
Pa10	95 (E)	2006 (0)	**DK12**
	461 (L)	2010 (4)	
Pa11	205 (E)	2004 (0)	**DK27**
	214 (L)	2009 (5)	
Pa12	220 (E)	2009 (0)	**DK31**
	216 (L)	2012 (3)	
Pa13	276 (E)	2009 (0)	**DK36**
	285 (L)*	2012 (3)	

Reference strain

E = early isolate

L= late isolate

* Mucoid phenotype
