# Supplementary material for: Pseudomonas aeruginosa induces the generation of pathogenic IFNγ+Th17 cells that promote lung damage and chronic inflammation in cystic fibrosis
Source: Nat Microbiol. 2026 Sep 24;11(11):3208–23. doi: 10.1038/s41564-026-02469-2 (PMC13739742; doi:10.1038/s41564-026-02469-2)
Supplement: Supplementary file 2 [file 41564_2026_2469_MOESM2_ESM.pdf]

nature portfolio

Corresponding author(s): Moira Paroni
Clelia Peano

Last updated by author(s): Jul 22, 2026

# Reporting Summary

Nature Portfolio wishes to improve the reproducibility of the work that we publish. This form provides structure for consistency and transparency in reporting. For further information on Nature Portfolio policies, see our Editorial Policies and the Editorial Policy Checklist.

## Statistics

For all statistical analyses, confirm that the following items are present in the figure legend, table legend, main text, or Methods section.

| n/a | Confirmed | |
|---|---|---|
| ☐ | ☒ | The exact sample size (*n*) for each experimental group/condition, given as a discrete number and unit of measurement |
| ☐ | ☒ | A statement on whether measurements were taken from distinct samples or whether the same sample was measured repeatedly |
| ☐ | ☒ | The statistical test(s) used AND whether they are one- or two-sided<br>*Only common tests should be described solely by name; describe more complex techniques in the Methods section.* |
| ☒ | ☐ | A description of all covariates tested |
| ☐ | ☒ | A description of any assumptions or corrections, such as tests of normality and adjustment for multiple comparisons |
| ☐ | ☒ | A full description of the statistical parameters including central tendency (e.g. means) or other basic estimates (e.g. regression coefficient) AND variation (e.g. standard deviation) or associated estimates of uncertainty (e.g. confidence intervals) |
| ☐ | ☒ | For null hypothesis testing, the test statistic (e.g. *F*, *t*, *r*) with confidence intervals, effect sizes, degrees of freedom and *P* value noted<br>*Give P values as exact values whenever suitable.* |
| ☒ | ☐ | For Bayesian analysis, information on the choice of priors and Markov chain Monte Carlo settings |
| ☒ | ☐ | For hierarchical and complex designs, identification of the appropriate level for tests and full reporting of outcomes |
| ☐ | ☒ | Estimates of effect sizes (e.g. Cohen's *d*, Pearson's *r*), indicating how they were calculated |

*Our web collection on statistics for biologists contains articles on many of the points above.*

## Software and code

Policy information about availability of computer code

Data collection

The plate-based absorbance detections were performed using SAFAS microplate reader - version V3.25 software.
Cell-sorting and Flow Cytometric analysis were performed with FACSAria III (BD Biosciences), Aurora CS (Cytek), FACS CANTOII (BD Biosciences), and Northern Light (Cytek).
Confocal microscopy images were captured using a Nikon Yokogawa CSU W1 spinning disk confocal microscope.
RNA- and TCR-sequencing were performed using the NovaSeq6000 (Illumina).

| | |
|---|---|
| Data analysis | Statistical analyses and graph plotting were carried out using Graphpad Prism v.10.<br>Confocal microscopy images were processed using ImageJ v1.54.<br>Data analysis of flow cytometry was performed with FlowJo v10.8.1. and CRUSTY (https://crusty.humanitas.it) for clustering analysis.<br>RNA sequencing: reads were aligned to the "analysis-set" GRCh38.p14 reference assembly of the human genome using STAR, and gene expression levels were quantified by RSEM on the Refseq annotation of the reference GRCh38 assembly of the human genome. Reads counts were batch-corrected for donors using Combat-seq. Differential gene expression analyses were performed by the exact test for a difference in means between two groups of negative binomial random variables, as implemented by the exactTest function in edgeR. Functional enrichment analyses for the identification of underlying molecular pathways were executed by GSEA version 4.4.0. Genes were ranked for GSEA by a signed $\log_2$ fold-change weighted by $-\log_{10}$ FDR (logFDR) to incorporate both effect size and statistical significance.<br>TCR-RNA sequencing: Raw FASTQ files were processed with MiXCR (version 4.7.0) employing the analyze takara-human-rna-tcr-umi-smarter-v2 pipeline. Clonotypes were assembled based on CDR3 sequences utilizing the --assemble-clonotypes by CDR3 parameter and the human genome (--species hsa).<br>Repertoire diversity, clonal expansion, and clonotype distributions were examined individually for TCR α and β chains employing the Immunarch R package (version 1.0.0). The overlap among repertoires was quantitatively assessed utilizing VDJtools (version 1.2.1). |

For manuscripts utilizing custom algorithms or software that are central to the research but not yet described in published literature, software must be made available to editors and reviewers. We strongly encourage code deposition in a community repository (e.g. GitHub). See the Nature Portfolio guidelines for submitting code & software for further information.

## Data

Policy information about availability of data

All manuscripts must include a data availability statement. This statement should provide the following information, where applicable:

- Accession codes, unique identifiers, or web links for publicly available datasets
- A description of any restrictions on data availability
- For clinical datasets or third party data, please ensure that the statement adheres to our policy

RNA- and TCR-sequencing data generated in this work have been deposited in the ArrayExpress database (https://www.ebi.ac.uk/biostudies/arrayexpress) under the accession numbers E-MTAB-15774 for RNA-seq, and E-MTAB-15769 for TCR-RNA-seq. Additional data supporting the findings of this study are available from the corresponding author upon request (moira.paroni@unimi.it).

## Research involving human participants, their data, or biological material

Policy information about studies with human participants or human data. See also policy information about sex, gender (identity/presentation), and sexual orientation and race, ethnicity and racism.

| | |
|---|---|
| Reporting on sex and gender | Data were collected randomly from subjects of both biological sexes. The sex of participants (male/female) was recorded in a pseudo-anonymized manner, as summarized in Supplementary Table 2. No sex- or gender-disaggregated analyses were performed due to limited sample size; therefore, data were analyzed collectively. Gender identity and presentation were not assessed in this study. Dendritic cells and Th-cell subsets, isolated from blood and lungs of subjects with or without Cystic Fibrosis, were used for all downstream analyses. |
| Reporting on race, ethnicity, or other socially relevant groupings | Race and ethnicity were not tracked in this study. |
| Population characteristics | see above |
| Recruitment | Human blood samples from CF and healthy donor, as well as lungs from CF and non-CF subjects undergoing lung transplantation for end-stage lung diseases, were collected from patients enrolled at the Thoracic Surgery and Lung Transplant Unit, and at the CF center of the Foundation IRCCS Ca' Granda, Ospedale Maggiore Policlinico, Milan-Italy. |
| Ethics oversight | Ethics statement<br>The studies involving human participants were carried out in compliance with the Declaration of Helsinki protocols and were reviewed and approved by the Ethics commission (Milano Area 2, code 15_2021). Written informed consent was obtained from all human participants included in the study.<br>Human bronchial epithelial cells were obtained from the facility (Servizio Colture Primarie) of the Italian Foundation for Cystic Fibrosis Research (FFC Ricerca).<br>The procedures for obtaining human bronchi from explanted lungs to establish epithelial cell cultures were approved by the appropriate Ethics Committee under registration ANTECER 042-09/07/2018.<br>All donors signed an informed consent using a form that was also approved by the Ethics Committee.<br>Personal data from all subjects were anonymized, and all data were handled in accordance with applicable privacy laws. |

Note that full information on the approval of the study protocol must also be provided in the manuscript.

# Field-specific reporting

Please select the one below that is the best fit for your research. If you are not sure, read the appropriate sections before making your selection.

☒ Life sciences ☐ Behavioural & social sciences ☐ Ecological, evolutionary & environmental sciences

For a reference copy of the document with all sections, see nature.com/documents/nr-reporting-summary-flat.pdf

# Life sciences study design

All studies must disclose on these points even when the disclosure is negative.

| | |
|---|---|
| Sample size | No statistical methods were used to pre-determine sample size. Sample size was determined according to established best practices in the respective field for each analysis. |
| Data exclusions | No data was excluded. |
| Replication | At least three independent experiments, each with a minimum of three replicates, were performed for all in vitro and tissue culture assays. The number of independent experiments is reported in the figure legends and in the Methods section. |
| Randomization | Not applicable. |
| Blinding | Not applicable. |

# Reporting for specific materials, systems and methods

We require information from authors about some types of materials, experimental systems and methods used in many studies. Here, indicate whether each material, system or method listed is relevant to your study. If you are not sure if a list item applies to your research, read the appropriate section before selecting a response.

## Materials & experimental systems

| n/a | Involved in the study | |
|---|---|---|
| ☐ | ☒ | Antibodies |
| ☒ | ☐ | Eukaryotic cell lines |
| ☒ | ☐ | Palaeontology and archaeology |
| ☒ | ☐ | Animals and other organisms |
| ☒ | ☐ | Clinical data |
| ☒ | ☐ | Dual use research of concern |
| ☒ | ☐ | Plants |

## Methods

| n/a | Involved in the study | |
|---|---|---|
| ☒ | ☐ | ChIP-seq |
| ☐ | ☒ | Flow cytometry |
| ☒ | ☐ | MRI-based neuroimaging |

## Antibodies

| | |
|---|---|
| Antibodies used | Immunostaining antibodies for flow cytometric and immunofluorescence analyses:<br>Anti-human CD4 (RPA-T4; BD Biosciences, 746541); anti-human CD127 (HIL-7R-M21; BD Biosciences, 566398); anti-human CD25 (2A3; BD Biosciences, 564467); anti-human CD196 (G034E3; BioLegend, 353401); anti-human CD183 (G025H7; BioLegend, 353705); anti-human CD195 (2D7/CCR5; BD Biosciences, 562576); anti-human CD154 (24-31; BioLegend, 310832); anti-human IL-17A (BL168; BioLegend, 512310); anti-human IFNγ (4S.B3; BioLegend, 502542); anti-human BATF3 (841702; Novus Biologicals, FAB7437Z); anti-human C/EBPδ (polyclonal; Novus Biologicals, NBP2-41192AF405); anti-human PPARγ (polyclonal; Novus Biologicals, NBP2-22106PE); anti-human CD14 MicroBeads (Miltenyi Biotec, 130-097-052); anti-human ZO-1 (ZO1-1A12; Thermo Scientific™, 33-9100; dilution 1:500); anti-human acetylated tubulin (IgG2 6-11B-1; Sigma-Aldrich, T7451; dilution 1:300); goat anti-mouse IgG1 Alexa Fluor™ 546 conjugate (Thermo Scientific™, A-21123); goat anti-mouse IgG2b Alexa Fluor™ 647 conjugate (Thermo Scientific™, A-21242).<br>anti-human CD40(5C3; BioLegend, 334330),anti-human CD80 (2D10; BioLegend, 305206), anti-human CD83 (HB15e; BioLegend, 305330), anti-human CD86 (bu63; BioLegend, 374208), anti-human CCR7 (G043H7; BioLegend, 353272), anti-human CD206 (15-2; BioLegend, 321152), anti-human CD209 (9E9A8; BioLegend, 330106), anti-human CD172a (SE5A5; BioLegend, 323814), anti-human CD274 (29E.2A3; BioLegend, 329722), anti-human CD366 (A18087E; BioLegend, 364806). |
| Validation | All the commercial antibodies were used according to manufacturer's protocol, titrated and used at the optimal working dilution. |

## Plants

Seed stocks

Report on the source of all seed stocks or other plant material used. If applicable, state the seed stock centre and catalogue number. If plant specimens were collected from the field, describe the collection location, date and sampling procedures.

Novel plant genotypes

Describe the methods by which all novel plant genotypes were produced. This includes those generated by transgenic approaches, gene editing, chemical/radiation-based mutagenesis and hybridization. For transgenic lines, describe the transformation method, the number of independent lines analyzed and the generation upon which experiments were performed. For gene-edited lines, describe the editor used, the endogenous sequence targeted for editing, the targeting guide RNA sequence (if applicable) and how the editor was applied.

Authentication

Describe any authentication procedures for each seed stock used or novel genotype generated. Describe any experiments used to assess the effect of a mutation and, where applicable, how potential secondary effects (e.g. second site T-DNA insertions, mosiacism, off-target gene editing) were examined.

## Flow Cytometry

### Plots

Confirm that:

☒ The axis labels state the marker and fluorochrome used (e.g. CD4-FITC).

☒ The axis scales are clearly visible. Include numbers along axes only for bottom left plot of group (a 'group' is an analysis of identical markers).

☒ All plots are contour plots with outliers or pseudocolor plots.

☒ A numerical value for number of cells or percentage (with statistics) is provided.

### Methodology

Sample preparation

Peripheral Th-cell subsets were isolated from heparinized blood samples after density gradient separation by Lympholyte. Pulmonary Th-cell subsets were isolated from explanted lung tissues, which were processed immediately after transplantation in calcium- and magnesium-free Hanks' balanced salt solution [HBSS, Euroclone], supplemented with 1% penicillin/streptomycin and 300 µg/mL gentamycin (HBSS-P/S/G). Tissues were incubated for 15 minutes in HBSS containing dithiothreitol [DTT, 0.16 mg/ml, Sigma-Aldrich] to remove viscous mucus and pulmonary bacteria. After extensive washing, samples were incubated for additional 60 min at 37°C in HBSS-P/S/G supplemented with 1mM EDTA (Sigma). Tissues were then digested for 1-3h at 37°C in RPMI 1640 medium containing 1mg/ml Collagenase D (Roche) in a 5% $CO_2$ incubator. Pulmonary mononuclear cells released from lung tissues were filtered through a 70-µm cell strainer and washed with complete RPMI 1640 medium supplemented with 10% FBS, before cell sorting. Total lymphocytes were stained, and Th1 and Th17 cell subsets were sort-purified according to the expression of specific surface markers combination within CD4+IL-7R +CD25low cells: Th1 cells (CCR6-CXCR3+), cTh17 cells (CCR6+CXCR3-CCR5-), pTh17 cells (CCR6+CXCR3-CCR5+), Th1/17- cells (CCR6+CXCR3+CCR5-), Th1/17+ cells (CCR6+CXCR3+CCR5+).
Intracellular cytokine expression was evaluated in cTh17 and Th1 cells following polyclonal stimulation with 50 ng/mL Phorbol 12-myristate 13-acetate (PMA) and 1 µg/mL Ionomycin for 2h. Brefeldin A (BFA, 10 µg/mL) was then added for additional 5-6h, after which cells were fixed with 2% paraformaldehyde solution (PFA). Following permeabilization with PBS-0.5% saponin, cells were stained with anti-CD40L, anti-IL-17A, and anti-IFNγ for 30 min at 4°C. Background activation of cTh17 or Th1 cells stimulated with autologous uninfected moDCs was subtracted to calculate antigen-dependent cells trans-differentiation, after the same polyclonal stimulation.
To analyse the expression of the transcription factors PPARγ, C/EBPδ, and BATF3 among cTh17 cells expressing IL-17 and/or IFNγ, cells were restimulated with PMA/Ionomycin and stained intracellularly using the FOXP3 Fix/Perm kit (eBioscience) with antibodies against PPARγ, C/EBPδ, and BATF3, according to the manufacturer's protocol.
Human monocyte-derived dendritic cells (moDCs) were generated from peripheral blood monocytes of healthy donors and CF subjects by CD14-positive selection (CD14 MicroBeads, Miltenyi Biotec) following density gradient separation. moDCs were differentiated for 7 days in the presence of recombinant human GM-CSF (50 ng/mL) and IL-4 (20 ng/mL). The immunophenotypic activation of moDC following 24 h infection with P. aeruginosa clinical strains was evaluated by flow cytometry assessing the expression of CD40, CD80, CD83, CD86, CCR7, CD206 (mannose receptor, MR), CD209 (DC-SIGN), CD172a (SIRPα), CD274 (PD-L1) and CD366 (TIM-3).

Instrument

Cell sorting was performed using a FACSAria III (BD Biosciences) or an Aurora CS (Cytek).
Flow cytometric acquisition was carried out with a FACSCanto II (BD Biosciences) or a NorthernLight cytometer (Cytek).

Software

Software: BD FACS DIVA and SpectroFlo®.
Data analysis: FlowJo v10.8.1. and CRUSTY (https://crusty.humanitas.it) for clustering analysis.

Cell population abundance

The abundance of the relevant Th-cell populations varied among individual human samples and can be evaluated in Fig. 1c and 1d of the main manuscript. The purity of each sorted sample was assessed immediately after sorting using a purity gating strategy and was consistently greater than 90%.

Gating strategy

A first physical gate was applied on SSC-A vs FSC-A, followed by singlet discrimination using FSC-H vs FSC-A. Within the $CD4^+$ population, a subsequent gate was set on IL-$7R^+$CD25^low cells, excluding regulatory T cells (CD25^high). Th1 and Th17 cell subsets were then sorted based on CXCR3, CCR6, and CCR5 expression, as shown in Extended Data Fig. 1a.

☒ Tick this box to confirm that a figure exemplifying the gating strategy is provided in the Supplementary Information.
