# Supplementary material for: Pseudomonas aeruginosa induces the generation of pathogenic IFNγ+Th17 cells that promote lung damage and chronic inflammation in cystic fibrosis
Source: Nat Microbiol. 2026 Sep 24;11(11):3208–23. doi: 10.1038/s41564-026-02469-2 (PMC13739742; doi:10.1038/s41564-026-02469-2)
Supplement: Supplementary file 3 [file 41564_2026_2469_MOESM3_ESM.pdf]

nature portfolio

Peer Review File

Pseudomonas aeruginosa induces the generation of pathogenic IFNγ⁺Th17 cells that promote lung damage and chronic inflammation in Cystic Fibrosis

Corresponding Author: Professor Moira Paroni

Version 0:

Decision Letter:

8th January 2026

*Please ensure you delete the link to your author homepage in this e-mail if you wish to forward it to your co-authors.

Dear Professor Paroni,

Thank you for your patience while your manuscript "Pseudomonas aeruginosa drives pathogenic IFNγ⁺Th17 cell generation with divergent transcriptomic signatures and TCR clonotypes in Cystic Fibrosis" was under peer-review at Nature Microbiology. It has now been seen by 3 referees, whose expertise and comments you will find at the end of this email. Although they find your work of some potential interest, they have raised a number of concerns that will need to be addressed before we can consider publication of the work in Nature Microbiology.

In particular, the reviewers want you to provide more information on the cohorts and methods, acknowledge the technical limitations, add clarifications, temper conclusions, add quantifications, look for correlation between the transcriptomics modules with clinical features, and add more mechanistic insights on the role of DCs.

Should further experimental data allow you to address these criticisms, we would be happy to look at a revised manuscript.

We are committed to providing a fair and constructive peer-review process. Please do not hesitate to contact us if there are specific requests from the reviewers that you believe are technically impossible or unlikely to yield a meaningful outcome.

We strongly support public availability of data. Please place the data used in your paper into a public data repository, if one exists, or alternatively, present the data as Source Data or Supplementary Information. If data can only be shared on request, please explain why in your Data Availability Statement, and also in the correspondence with your editor. For some data types, deposition in a public repository is mandatory - more information on our data deposition policies and available repositories can be found at https://www.nature.com/nature-research/editorial-policies/reporting-standards#availability-of-data.

Please include a data availability statement as a separate section after Methods but before references, under the heading "Data Availability". This section should inform readers about the availability of the data used to support the conclusions of your study. This information includes accession codes to public repositories (data banks for protein, DNA or RNA sequences, microarray, proteomics data etc…), references to source data published alongside the paper, unique identifiers such as URLs to data repository entries, or data set DOIs, and any other statement about data availability. At a minimum, you should include the following statement: "The data that support the findings of this study are available from the corresponding author upon request", mentioning any restrictions on availability. If DOIs are provided, we also strongly encourage including these in the Reference list (authors, title, publisher (repository name), identifier, year). For more guidance on how to write this section please see:
http://www.nature.com/authors/policies/data/data-availability-statements-data-citations.pdf

If revising your manuscript:

* Include a "Response to referees" document detailing, point-by-point, how you addressed each referee comment. If no action was taken to address a point, you must provide a compelling argument. This response will be sent back to the referees along with the revised manuscript.

* If you have not done so already we suggest that you begin to revise your manuscript so that it conforms to our Article format instructions at http://www.nature.com/nmicrobiol/info/final-submission. Refer also to any guidelines provided in this letter.

* Include a revised version of any required reporting checklist. It will be available to referees (and, potentially, statisticians) to aid in their evaluation if the manuscript goes back for peer review. A revised checklist is essential for re-review of the paper.

When submitting the revised version of your manuscript, please pay close attention to our href="https://www.nature.com/nature-portfolio/editorial-policies/image-integrity">Digital Image Integrity Guidelines. and to the following points below:

-- that unprocessed scans are clearly labelled and match the gels and western blots presented in figures.
-- that control panels for gels and western blots are appropriately described as loading on sample processing controls
-- all images in the paper are checked for duplication of panels and for splicing of gel lanes.

EXTENDED DATA FIGURES

When re-submitting your manuscript, please ensure that any supplementary figures and tables that are crucial to the manuscript's conclusions are converted into Extended Data figures and tables to increase visibility of these data. Extended Data figures and tables are online-only (present in the online PDF and full-text HTML versions of the paper), peer-reviewed display items that provide essential background to the article but are not included in the main article due to space constraints. A maximum of ten Extended Data display items (figures and tables) is permitted.

Finally, please ensure that you retain unprocessed data and metadata files after publication, ideally archiving data in perpetuity, as these may be requested during the peer review and production process or after publication if any issues arise.

Please use the link below to submit a revised paper:

Link Redacted

Note: This url links to your confidential homepage and associated information about manuscripts you may have submitted or be reviewing for us. If you wish to forward this e-mail to co-authors, please delete this link to your homepage first.

Nature Microbiology is committed to improving transparency in authorship. As part of our efforts in this direction, we are now requesting that all authors identified as 'corresponding author' on published papers create and link their Open Researcher and Contributor Identifier (ORCID) with their account on the Manuscript Tracking System (MTS), prior to acceptance. This applies to primary research papers only. ORCID helps the scientific community achieve unambiguous attribution of all scholarly contributions. You can create and link your ORCID from the home page of the MTS by clicking on 'Modify my Springer Nature account'. For more information please visit please visit www.springernature.com/orcid.

If you wish to submit a suitably revised manuscript we would hope to receive it within 6 months. If you cannot send it within this time, please let us know. We will be happy to consider your revision, even if a similar study has been accepted for publication at Nature Microbiology or published elsewhere (up to a maximum of 6 months).

In the meantime we hope that you find our referees' comments helpful.

Yours sincerely,

[redacted]

****

Reviewer Expertise:

Referee #1: Cystic fibrosis, Pseudomonas aeruginosa
Referee #2: Mucosal immunity, Th17
Referee #3: Cystic Fibrosis, Th17, transcriptomics

Reviewer Comments:

Reviewer #1 (Remarks to the Author):

This manuscript by Dusetti et al. aims to provide direct evidence linking the activation of IFNγ-producing Th17 subsets to

lung tissue damage and to address the current lack of evidence supporting the suspected pathogenic role in CF, particularly through IL-17–mediated neutrophil recruitment and epithelial barrier disruption. The authors should be commended for their comprehensive, well-structured and muti-facetted approach. The key observations include: (a) identification of Th1/17⁺ and pTh17 cells as pathogenic effectors in CF lungs, with preferential expansion in P. aeruginosa infected airways and associated compromise of epithelial integrity; (b) demonstrate that these subsets exhibit transcriptional reprogramming and clonal expansion within the lung microenvironment; and (c) evidence that P. aeruginosa persists within dendritic cells, driving IL-1β and IL-23 production that primes IFNγ⁺ Th17 differentiation. According to the authors, these findings reveal a significant ability of P. aeruginosa to reprogram protective cTh17 cells into pathogenic Th1/17⁺ and pTh17 effectors, thereby sustaining epithelial damage and chronic inflammation in CF.
The manuscript presents compelling evidence however there are several concerns and areas that need greater clarity surrounding cohort definitions, methodology and acknowledgement of technical limitations. Please see below our point-by-point response.

Major concern:
1. Greater clarity is needed regarding the patient cohorts and their characteristics.
a. Firstly, for the Healthy donor / non-CF lung cohort. There is almost no information provided for this group. Given that these individuals were undergoing lung transplantation, it is likely that they had substantial underlying pathology. The manuscript would benefit from more detail about this cohort, including relevant co-morbidities and, where possible, the underlying diagnoses that led to lung transplantation.
b. The CF cohorts:
i. For the lung group, there are more bacteria listed than patients, implying co-infection in some people; please provide more detail
ii. The Pa infected group is reported to have higher numbers of cells of interest than the others. This may well be bacteria-specific, but without more information on the individuals, we cannot rule out a confounder; as Pa is associated with poorer clinical outcomes than some of the other infections, and to be commoner in people with ‘severe’ CFTR variants, we need to see more detailed clinical metadata (suggest at least: sex, age, CFTR variant status, FEV1, on CFTR modulators/ not, on anti-inflammatories (incl azithromycin)/ not) and if there are differences between the Pa+ and – groups, to be reassured that these have been adjusted for in the analysis.
iii. Is there any overlap between the CF lung and CF blood cohorts? With its stricter exclusion criteria were patients in the CF blood group derived from the same individuals as the CF lung cohort or represents an entirely separate group. A similar uncertainty applies to the healthy donor cohorts.
1. Why does the CF blood cohort lack any microbial data? We need this information to support interpretation of the data. This point is especially important given that the authors highlight a compartmentalised lung effect when comparing CF lung with CF blood; however, the observed differences may also reflect variation in infection status rather than purely tissue-specific effects.
2. It would also be valuable for the authors to explain why the stricter exclusion criteria used for the CF blood cohort were not applied consistently across all groups, and to discuss any implications this may have for interpreting the findings.
iv. A potential recommendation to better profile the cohorts would perhaps be an additional supplementary table listing individual patients and their characteristics. This would be highly informative for clinically focused readers and would provide helpful context for interpreting variability across cohorts.
c. Finally, in line with the above, given the use of explanted tissues, there is an inherent bias toward advanced CF disease. How do the authors think disease severity might have influenced their findings and generalisability? Would similar effects be expected in mild or moderate disease, and how might this impact the proposed use of Th1/17+ and pTh17 subsets as alternative targets for CF immune therapies aimed at restoring immune homeostasis?
d. A growing challenge in both clinical management of CF and CF research is the classification of chronic infection, particularly following the introduction of highly effective modulator therapy. It would be helpful if the authors could clarify how chronic infection was defined in this study, including any specific criteria used. Additionally, please provide details on the proportion of patients with Pseudomonas aeruginosa in isolation compared to those with polymicrobial cultures. The table suggests that from 19 patients, 28 pathogens (including AF) were identified, could the authors therefore provide further details since their analysis would suggest a more binary classification of Pa positive in isolation vs other pathogens rather than polymicrobial cultures.
e. It would help the reader if the section headers included CF or non-CF
2. Figures (see more in minor, but taken together, this is an overarching issue, so flagged here)
a. In Fig 1a why are there such small n, given that similar data appear elsewhere to include the full group sizes?
b. Fig 1b: please review the legend and make this figure more understandable and add details, eg CF/ non-CF; describe what the reader should be expected to take away from the heatmap. Same for other potentially non-intuitive figs throughout.
3. Analysis:
a. The term ‘correlate’ is used quite extensively; in some cases, the authors mean ‘two groups separated on the basis of a feature (eg Pa+/-) had statistically different values of something. I think ‘associated with’ would be more accurate
b. In other places there seem to have been multiple ‘correlations’ explored, without details of which test was used and no mention of whether any adjustment has been made for the multiple comparisons.
4. Conclusions: largely well-written, but the authors have not included their own evaluation of their study having not included any details of potential strengths and more importantly limitations to their study. This I believe is an important part of any discussion and should be addressed by the authors.

Minor issues:

The authors have appropriately addressed technical variation through the use of ComBat-seq. However, it is unclear whether similar measures were taken to account for donor-related variability (e.g., age, disease severity, treatment history). Clarification on whether steps were implemented to mitigate these potential confounders would be important especially given the limited number if replicates per group.

Can the authors clarify and justify the chosen co-culture duration for the P. aeruginosa infection assays of moDCs? The manuscript states (lines 173–174) that selective expansion of Th1/17+ and pTh17 cells occurs only in chronically infected CF lungs. However, the model used involves co-culturing cells with P. aeruginosa for 90 minutes, with a maximum readout at 24 hours. Is this timeframe sufficient to reflect chronic infection dynamics?

Could the authors clarify why TEER measurements were not included to assess barrier integrity alongside structural markers such as ZO-1?

Figures: Please ensure figures and captions are looked at carefully, there are some areas that need closer attention and would benefit from being revised. Examples include 1D: the blue is easy, but then the use of the three shades of purple and mix of filled/ empty symbols with empty/ filled boxes is very confusing.

Please can the authors review the figure captions. There appears to be some inconsistency with information provide, namely numbers per group/cohorts used in the figure or analysis. This appears to be included in some of the figure legends yet it is missing from others. Please can the authors ensure consistency and provide these details.

Figures 2A and S2A/B:
Please could the authors expand the figure captions for the heatmaps in Figures 2a and S2a/b to provide sufficient contextual detail. According to the methodology, the analysis appears to include a small number of samples (CF blood: n = 3; CF lung: n = 4; non-CF lung: n = 4). Furthermore, it would be helpful if the authors could indicate what proportion of these samples were classified as chronic P. aeruginosa or not. If possible, it would be nice to add this information directly on the figures to assist interpretation.
In addition, as stated in lines 136–139, the authors note that when considering CF samples alone, there is a greater differentiation between CF blood and CF lung. However, this separation appears to arise primarily from the revised hierarchical clustering performed after excluding the non-CF group. In contrast, the original clustering including all groups suggests a lesser degree of separation, with CF blood samples clustering more closely with the non-CF lung group. This is an interesting observation; it would benefit the manuscript if the authors clarified this point more explicitly in the manuscript and corresponding figure legends.

The manuscript, whilst well written, can at times be difficult to follow, due to the number of analytic approaches incorporated and often assumed understanding. Efforts should therefore be made to improve readability and accessibility for wider audiences with perhaps less of a fundamental science background.

Line 88-90: “Our findings highlight Th1/17+ and pTh17 subsets, and the immunological processes upstream of their generation, as promising targets for novel and more selective immunomodulatory therapies in CF, offering a potential alternative to current broad-spectrum anti-inflammatory approaches.” This sentence requires more cautious interpretation. While the data supports the first part of the statement, the latter part was not the primary aim of the study and is therefore speculative. Please revise to clearly indicate that this is a putative interpretation rather than a conclusion drawn from the study.

Line 84: TCR repertoire – Please ensure use of ‘T-cell receptor repertoire’ ahead of abbreviation.

Table S2: Please can the authors clarify what is meant by the one patient listed in the non-CF lung group with CF related diabetes. This is likely reflecting either a typo or mislabelling of a non-CF lung patient with diabetes as CFRD.

Reviewer #2 (Remarks to the Author):

In this paper by Dusetti et al, the authors identify two pathogenic Th17 subsets—Th1/17^{+} and pTh17 cells which are selectively enriched in CF lungs specially colonized by Pseudomonas aeruginosa. The authors show that these pathogenic IFNγ+Th17 subsets are derived from cTh17 precursors. Using ALI cultures, the authors show that upon activation they drive disruption of the pulmonary epithelial integrity and trigger exaggerated cytokine and chemokine responses. Using bulk RNA seq of sorted populations from CF and non-CF samples, transcriptomic and TCR profiling reveals functional details regarding the clonal selection of Th1/17^{+} and pTh17 cells in CF lungs. This to me, is the most exciting part of the paper and the most novel- while there have been projections that Th17 and IL-17 have a role to play in CF pathology, the current study very elegantly using human samples from blood and lung to make this connection in a mechanistic way.

In the second part of the paper, they try and bridge the observation from early figs using human samples to using in vitro models – for examples using clinical P. aeruginosa strains that persist within dendritic cells they show that this induces IL-1β/IL-23 cytokine in DCs and promotes IFNγ-producing Th17 differentiation over classical Th1 lineage commitment – this is not especially surprising. It is however interesting that early isolates that probably lack lacking chronic adaptation modules can still potently reprogram cTh17 cells.

Major points:

1. The link between CF, Pseudomonas infection, and Th17 skewed inflammation is very nicely established, giving the work very high conceptual novelty.

2. The integration of lung vs blood comparisons, transcriptomics, and TCR clonotyping anchored to clinically relevant P. aeruginosa strains fits well- this is extremely difficult to do and I applaud the authors for their courage in sorting these population to carry out this difficult work.

3. The additional mechanistic studies in vitro while appealing and add some mechanistic angle is not entirely novel. I would keep the focus on the early data.

I have a few suggestions to improve some of the elements of the paper:

1. Can the ZO-1 staining and barrier integrity be quantitated and included- as it stands, unless you are an expert you wouldn't know what to look for- also add some highlights within the figures to guide the readers eye. .

2. For any of the readouts from CF patients can they look for correlation between their transcriptomics modules with clinical features such as lung function or exacerbation frequency?

3. They make very strong interpretations and conclusions- since these are ex vivo analysed cell types, they should temper it, so it is a well balanced read.

Reviewer #3 (Remarks to the Author):

Dusetti et al. examined Th17 cells in CF lungs and, using flow cytometry and scRNA-seq, identified distinct Th17 subsets. Two of these subsets are enriched and clonally expanded in CF patients, as demonstrated by single-cell V(D)J sequencing. Functionally, these cells disrupt epithelial integrity in co-culture with air–liquid interface epithelial cells and drive epithelial production of pro-inflammatory cytokines and chemokines. The authors further show that clinical P. aeruginosa isolates infect dendritic cells, inducing IL-1β and IL-23, which could underlie the in vivo generation of pathogenic Th17 cells. In an in vitro human Th17 culture system, these clinical isolates skew protective cTh17 cells toward pathogenic IFNγ-producing Th17 subsets. Overall, the data are novel and have the potential to reveal new targets for more selective immunomodulatory therapies in CF. However, the manuscript could be improved in the following aspects:

Nomenclature. The Th17 nomenclature is confusing. According to Fig. S1A, the authors focus on $IFN\gamma^{+}IL\text{-}17^{+}$ cells termed "Th1/17⁺," $CCR5^{+}IL\text{-}17^{+}$ cells termed "pTh17" (it is unclear what "p" stands for), and $CCR5^{-}IL\text{-}17^{+}$ cells termed conventional Th17 cells. Clearer definitions, justification for these terms, and consistent use throughout the manuscript would help readers follow the subsets being discussed.

Sample size in Fig. 1. It is not clear why only 3–4 patients per group are shown in Fig. 1 when 19 CF patients were recruited. The authors should either include data from all available patients or explain the criteria for inclusion/exclusion and whether the shown cohort is representative of the full study population.

Clonality and microbial status. In Fig. 2, the clonality analysis does not appear to account for Pseudomonas status. Are the expanded clones predominantly found in P. aeruginosa–colonized lungs, or do other bacterial species also drive similar clonal expansions? Stratifying the clonal data by microbial colonization (e.g., P. aeruginosa vs. other pathogens or polymicrobial states) would substantially strengthen the interpretation.

Mechanistic role of dendritic cells. In Fig. 5, additional mechanistic insight into dendritic cell responses would be valuable. Beyond showing that clinical isolates can induce $IFN\gamma^{+}IL\text{-}17^{+}$ cells, it would be informative to examine how these isolates reprogram DCs at the molecular level (e.g., transcriptomic changes, surface costimulatory molecules, cytokine/chemokine profiles). Such data would better define the DC-intrinsic pathways that promote pathogenic Th17 differentiation.

****

Version 1:

Decision Letter:

18th May 2026

*Please ensure you delete the link to your author homepage in this e-mail if you wish to forward it to your co-authors.

Dear Professor Paroni,

Thank you for your patience while your manuscript "*Pseudomonas aeruginosa* drives pathogenic IFNγ$^+$Th17 cell generation with divergent transcriptomic signatures and TCR clonotypes in Cystic Fibrosis" was under peer-review at Nature Microbiology. It has now been seen by 3 referees, whose expertise and comments you will find at the end of this email. You will see from their comments below that while they find your work of interest, there were still some issues. We are very interested in the possibility of publishing your study in Nature Microbiology, but would like to consider your response to these concerns in the form of a revised manuscript before we make a final decision on publication.

In particular, you will see that reviewer #1 wants you to do a comparison of cases with isolated P. aeruginosa infection and those with polymicrobial P. aeruginosa infection. The rest referees' reports are clear and the remaining issues should be straightforward to address.

We are committed to providing a fair and constructive peer-review process. Do not hesitate to contact us if there are specific requests from the reviewers that you believe are technically impossible or unlikely to yield a meaningful outcome.

If you have not done so already please begin to revise your manuscript so that it conforms to our Article format instructions at http://www.nature.com/nmicrobiol/info/final-submission/

The usual length limit for a Nature Microbiology Article is six display items (figures or tables) and 3,000 words. We have some flexibility, and can allow a revised manuscript at 3,500 words, but please consider this a firm upper limit. There is a trade-off of ~250 words per display item, so if you need more space, you could move a Figure or Table to Supplementary Information.

Some reduction could be achieved by focusing any introductory material and moving it to the start of your opening 'bold' paragraph, whose function is to outline the background to your work, describe in a sentence your new observations, and explain your main conclusions. The discussion should also be limited. Methods should be described in a separate section following the discussion, we do not place a word limit on Methods.

Nature Microbiology titles should give a sense of the main new findings of a manuscript, and should not contain punctuation. Please keep in mind that we strongly discourage active verbs in titles, and that they should ideally fit within 90 characters each (including spaces).

We strongly support public availability of data. Please place the data used in your paper into a public data repository, if one exists, or alternatively, present the data as Source Data or Supplementary Information. If data can only be shared on request, please explain why in your Data Availability Statement, and also in the correspondence with your editor. For some data types, deposition in a public repository is mandatory - more information on our data deposition policies and available repositories can be found at https://www.nature.com/nature-research/editorial-policies/reporting-standards#availability-of-data.

Please include a data availability statement as a separate section after Methods but before references, under the heading "Data Availability". This section should inform readers about the availability of the data used to support the conclusions of your study. This information includes accession codes to public repositories (data banks for protein, DNA or RNA sequences, microarray, proteomics data etc…), references to source data published alongside the paper, unique identifiers such as URLs to data repository entries, or data set DOIs, and any other statement about data availability. At a minimum, you should include the following statement: "The data that support the findings of this study are available from the corresponding author upon request", mentioning any restrictions on availability. If DOIs are provided, we also strongly encourage including these in the Reference list (authors, title, publisher (repository name), identifier, year). For more guidance on how to write this section please see:
http://www.nature.com/authors/policies/data/data-availability-statements-data-citations.pdf

To improve the accessibility of your paper to readers from other research areas, please pay particular attention to the wording of the paper's opening bold paragraph, which serves both as an introduction and as a brief, non-technical summary in about 150 words. If, however, you require one or two extra sentences to explain your work clearly, please include them even if the paragraph is over-length as a result. The opening paragraph should not contain references. Because scientists from other sub-disciplines will be interested in your results and their implications, it is important to explain essential but specialised terms concisely. We suggest you show your summary paragraph to colleagues in other fields to uncover any problematic concepts.

If your paper is accepted for publication, we will edit your display items electronically so they conform to our house style and will reproduce clearly in print. If necessary, we will re-size figures to fit single or double column width. If your figures contain several parts, the parts should form a neat rectangle when assembled. Choosing the right electronic format at this stage will speed up the processing of your paper and give the best possible results in print. We would like the figures to be supplied as vector files - EPS, PDF, AI or postscript (PS) file formats (not raster or bitmap files), preferably generated with vector-graphics software (Adobe Illustrator for example). Please try to ensure that all figures are non-flattened and fully editable. All images should be at least 300 dpi resolution (when figures are scaled to approximately the size that they are to be printed at) and in RGB colour format. Please do not submit Jpeg or flattened TIFF files. Please see also 'Guidelines for Electronic Submission of Figures' at the end of this letter for further detail.

Figure legends must provide a brief description of the figure and the symbols used, within 350 words, including definitions of any error bars employed in the figures.

When submitting the revised version of your manuscript, please pay close attention to our href="https://www.nature.com/nature-research/editorial-policies/image-integrity">Digital Image Integrity Guidelines. and to the following points below:

-- that unprocessed scans are clearly labelled and match the gels and western blots presented in figures.
-- that control panels for gels and western blots are appropriately described as loading on sample processing controls
-- all images in the paper are checked for duplication of panels and for splicing of gel lanes.

EXTENDED DATA FIGURES

When re-submitting your manuscript, please ensure that any supplementary figures and tables that are crucial to the manuscript's conclusions are converted into Extended Data figures and tables to increase visibility of these data. Extended Data figures and tables are online-only (present in the online PDF and full-text HTML versions of the paper), peer-reviewed display items that provide essential background to the article but are not included in the main article due to space constraints. A maximum of ten Extended Data display items (figures and tables) is permitted.

Finally, please ensure that you retain unprocessed data and metadata files after publication, ideally archiving data in perpetuity, as these may be requested during the peer review and production process or after publication if any issues arise.

Please include a statement before the acknowledgements naming the author to whom correspondence and requests for materials should be addressed.

Finally, we require authors to include a statement of their individual contributions to the paper -- such as experimental work, project planning, data analysis, etc. -- immediately after the acknowledgements. The statement should be short, and refer to authors by their initials. For details please see the Authorship section of our joint Editorial policies at http://www.nature.com/authors/editorial_policies/authorship.html

When revising your paper:

* include a point-by-point response to any editorial suggestions and to our referees. Please include your response to the editorial suggestions in your cover letter, and please upload your response to the referees as a separate document.

* ensure it complies with our format requirements for Letters as set out in our guide to authors at www.nature.com/nmicrobiol/info/gta/

* state in a cover note the length of the text, methods and legends; the number of references; number and estimated final size of figures and tables

* resubmit electronically if possible using the link below to access your home page:

Link Redacted

*This url links to your confidential homepage and associated information about manuscripts you may have submitted or be reviewing for us. If you wish to forward this e-mail to co-authors, please delete this link to your homepage first.

Please ensure that all correspondence is marked with your Nature Microbiology reference number in the subject line.

Nature Microbiology is committed to improving transparency in authorship. As part of our efforts in this direction, we are now requesting that all authors identified as 'corresponding author' on published papers create and link their Open Researcher and Contributor Identifier (ORCID) with their account on the Manuscript Tracking System (MTS), prior to acceptance. This applies to primary research papers only. ORCID helps the scientific community achieve unambiguous attribution of all scholarly contributions. You can create and link your ORCID from the home page of the MTS by clicking on 'Modify my Springer Nature account'. For more information please visit please visit www.springernature.com/orcid.

We hope to receive your revised paper within three weeks. If you cannot send it within this time, please let us know.

We look forward to hearing from you soon.

Yours sincerely,

[redacted]

****

Reviewers Comments:

Reviewer #1 (Remarks to the Author):

Overall I am very impressed with their detailed and very comprehensive response to our comments and the other reviewers' comments.

To recap, our primary concerns were primarily focused on:

Cohort definitions and patient characterisation
The definition of chronic infection and impact of polymicrobial infections

The authors have taken great steps to address this including individual patient tables with further granular detail on co-morbidities / disease severity etc. In addition to this they have conducted a correlation analysis to assess impact of possible clinical variables on the distribution of t-cell subsets. For this they showed that for cth17 and pTh17 cells in blood there was higher levels in those with more severe lung disease which they later expand upon with further analysis.

We would, however, welcome some further clarification. From Supplementary Table 3, it appears that there are 6 cases of chronic pulmonary P. aeruginosa infection in the CF lung cohort without co-culture of other pathogens, whereas the Extended Data Figure 2a appears to show more than 6 samples in the Pa+, Sa− group. This raises the possibility that the latter group may include cases with pathogens other than S. aureus. If that is the case, it would be helpful for the authors to state this explicitly and clarify the grouping strategy.

We fully appreciate, as the authors note in their response, that the sample size may limit the feasibility of analysing each pathogen separately. However, if possible, it may still be informative to compare cases with isolated P. aeruginosa infection and those with polymicrobial P. aeruginosa infection, including both S. aureus and other pathogens. Even if exploratory, such an analysis could help support the interpretation that the reported immune differences are associated with P. aeruginosa rather than reflecting polymicrobial infection more broadly.

Minor:
The authors have addressed the comments made with regards cohort definitions and the application of the stricter exclusion criterion in great detail and have partly incorporated this into the manuscript [line 442-445]. It would however perhaps be beneficial to the reader if the author included their justification that a stricter criterion was used to allow for an “unbiased assessment of peripheral immune cell frequencies and their comparison with healthy donors”.

We thank the authors who have diligently provided detailed individual patient characteristics where possible in order to provide greater cohort transparency. This information is vital for the reader to understand the cohorts better. In addition to the above, for table 2 it would also be useful for the authors to include in the table legend that for colonisation results reflect potentially > 1 cultured pathogen per patient.

Reviewer #2 (Remarks to the Author):

The authors have addressed my comments

Reviewer #3 (Remarks to the Author):

The authors have fully addressed my concerns.

****

Version 2:

Decision Letter:

Our ref: NMICROBIOL-25104127B

29th June 2026

Dear Dr. Paroni,

Thank you for submitting your revised manuscript "*Pseudomonas aeruginosa* drives pathogenic IFNγ^{+}Th17 cell generation with divergent transcriptomic signatures and TCR clonotypes in Cystic Fibrosis" (NMICROBIOL-25104127B). It has now been seen by the original referees and their comments are below. The reviewers find that the paper has improved in revision, and therefore we'll be happy in principle to publish it in Nature Microbiology, pending minor revisions to satisfy the referees' final requests and to comply with our editorial and formatting guidelines.

If the current version of your manuscript is in a PDF format, please email us a copy of the file in an editable format (Microsoft Word or LaTex)-- we can not proceed with PDFs at this stage.

We are now performing detailed checks on your paper and will send you a checklist detailing our editorial and formatting requirements in a couple of weeks. Please do not upload the final materials and make any revisions until you receive this additional information from us.

Thank you again for your interest in Nature Microbiology Please do not hesitate to contact me if you have any questions.

Sincerely,
[redacted]

Reviewer #1 (Remarks to the Author):

many thanks for this additional work; our comments have been adequately addressed

INTERNAL USE ONLY AIP modules required: ["Life Science"]

Open Access This Peer Review File is licensed under a Creative Commons Attribution 4.0 International License, which permits use, sharing, adaptation, distribution and reproduction in any medium or format, as long as you give appropriate credit to the original author(s) and the source, provide a link to the Creative Commons license, and indicate if changes were made.
In cases where reviewers are anonymous, credit should be given to 'Anonymous Referee' and the source.
The images or other third party material in this Peer Review File are included in the article's Creative Commons license,

unless indicated otherwise in a credit line to the material. If material is not included in the article's Creative Commons license and your intended use is not permitted by statutory regulation or exceeds the permitted use, you will need to obtain permission directly from the copyright holder.
To view a copy of this license, visit https://creativecommons.org/licenses/by/4.0/

Reviewer #1

This manuscript by Dusetti et al. aims to provide direct evidence linking the activation of IFNγ-producing Th17 subsets to lung tissue damage and to address the current lack of evidence supporting the suspected pathogenic role in CF, particularly through IL-17–mediated neutrophil recruitment and epithelial barrier disruption. The authors should be commended for their comprehensive, well-structured and muti-facetted approach. The key observations include: (a) identification of Th1/17$^+$ and pTh17 cells as pathogenic effectors in CF lungs, with preferential expansion in P. aeruginosa infected airways and associated compromise of epithelial integrity; (b) demonstrate that these subsets exhibit transcriptional reprogramming and clonal expansion within the lung microenvironment; and (c) evidence that P. aeruginosa persists within dendritic cells, driving IL-1β and IL-23 production that primes IFNγ$^+$ Th17 differentiation. According to the authors, these findings reveal a significant ability of P. aeruginosa to reprogram protective cTh17 cells into pathogenic Th1/17$^+$ and pTh17 effectors, thereby sustaining epithelial damage and chronic inflammation in CF.
The manuscript presents compelling evidence however there are several concerns and areas that need greater clarity surrounding cohort definitions, methodology and acknowledgement of technical limitations. Please see below our point-by-point response.

Major concern:

1. Greater clarity is needed regarding the patient cohorts and their characteristics.

a. Firstly, for the Healthy donor / non-CF lung cohort. There is almost no information provided for this group. Given that these individuals were undergoing lung transplantation, it is likely that they had substantial underlying pathology. The manuscript would benefit from more detail about this cohort, including relevant co-morbidities and, where possible, the underlying diagnoses that led to lung transplantation.

b. The CF cohorts:
i. For the lung group, there are more bacteria listed than patients, implying co-infection in some people; please provide more detail.

ii. The Pa infected group is reported to have higher numbers of cells of interest than the others. This may well be bacteria-specific, but without more information on the individuals, we cannot rule out a confounder; as Pa is associated with poorer clinical outcomes than some of the other infections, and to be commoner in people with 'severe' CFTR variants, we need to see more detailed clinical metadata (suggest at least: sex, age, CFTR variant status, FEV1, on CFTR modulators/ not, on anti-inflammatories (incl azithromycin)/ not) and if there are differences between the Pa+ and – groups, to be reassured that these have been adjusted for in the analysis.

We thank the reviewer for this important and constructive comment and fully agree that a detailed clinical characterization of the patient cohorts is essential to strengthen both the interpretation and the translational relevance of our findings.
To address this point, we have substantially expanded the description of all analysed cohorts and reorganized Supplementary Table 2 into three additional distinct tables (Supplementary Table 3-4-5), corresponding to: (i) CF lung, (ii) non-CF lung, and (iii) CF blood cohorts. These new 3 tables now report, within the limitations imposed by Italian privacy laws on patients, clinical and microbiological data at individual-level, including CFTR variants, lung function parameters (LAS and FEV_1pp, for lungs cohorts), microbial status at the time of sampling or transplantation, and ongoing therapies.

Dipartimento di Bioscienze
Via Celoria 26 - 20133 Milano, Italy
Tel 02503 15042 - Fax 02503 15044 - direzione.bioscienze@unimi.it

Due to the requirements of the local Ethics Committee, samples included in this study are subject to pseudonymization, which restricts the reporting of certain sensitive identifiers (specifically age and sex) at the individual-sample level when combined with other clinical variables, to avoid potential re-identification. For this reason, age and sex are reported in aggregated form in the separate summary Supplementary Table 2, in addition age distribution among all patient cohorts is displayed in the new Supplementary Fig.1. Similarly, detailed microbiological and clinical parameters are provided where ethically permissible, in aggregate form in Supplementary Table 2 and in pseudonymized form in Supplementary tables 3 - 4 - 5. For the same reason, CFTR genotypes are reported as functional classes (F, MF, and RF) rather than as individual mutations, which both complies with pseudonymization requirements and reflects established genotype–phenotype stratification in CF.

a. non-CF lung cohort: All non-CF lung samples were obtained from individuals undergoing lung transplantation for end-stage lung diseases, and they represent the most appropriate non-CF control tissue available for human lung–resident immune profiling. Relevant underlying diagnoses and comorbidities are now detailed in the new Supplementary Table 4, thus providing greater transparency regarding the pathological background of this cohort.

b.i. CF cohorts and microbial co-infection: As correctly noted by the reviewer, the number of bacterial species listed exceeds the number of CF lung samples, reflecting the presence of polymicrobial colonization in a subset of patients. We have now clarified this point by providing a more detailed breakdown of microbial status at the individual level in Supplementary Table 3 for CF-lung and Supplementary Table 5 for CF-blood.

b.ii. Potential confounders in Pa^{+} versus Pa^{-} CF cohorts: We acknowledge that, in addition to *P. aeruginosa* infection, also disease severity, CFTR genotype, and clinical outcomes potentially might affect our observations. To address the reviewer's concern, we have now expanded the available clinical metadata and performed additional stratified and correlative analyses within the CF and non-CF cohorts.
Specifically, we have explicitly examined the potential impact of key clinical variables on the immune phenotypes reported. We now include a new Supplementary Figure 1 presenting comprehensive correlation and stratification analyses between the frequency of all analysed T cell subsets and age (Figure S1a), lung function (FEV_1pp; Figure S1b), and microbial status, across all cohorts (Extended Data Fig. 2d), as requested. These analyses did not identify age- or lung-function–driven biases that could account for the enrichment of the identified T cell populations in Pa^{+} samples.

Together, these additions provide a more complete and transparent clinical framework for the studied cohorts and support the conclusion that the observed immunological differences are robust and not trivially explained by demographic or clinical confounders.

> iii. Is there any overlap between the CF lung and CF blood cohorts? With its stricter exclusion criteria were patients in the CF blood group derived from the same individuals as the CF lung cohort or represents an entirely separate group. A similar uncertainty applies to the healthy donor cohorts.

We thank the reviewer for raising this important point and apologize for any lack of clarity in the initial description of the cohorts.

The CF lung and CF blood samples represent **entirely distinct cohorts**, due to unavoidable clinical and ethical constraints associated with lung transplantation. Specifically, peripheral blood cannot be collected in the peri-transplant period, as CF patients are exposed to intensive immunosuppressive regimens both before and immediately after transplantation. These treatments profoundly alter

peripheral lymphocyte frequencies and phenotypes, precluding meaningful comparison with untreated individuals. Accordingly, the CF blood cohort was intentionally defined using strict exclusion criteria that ruled out patients receiving immunosuppressive therapies, in order to enable an unbiased assessment of peripheral immune cell frequencies and their comparison with healthy donors (HD). Importantly, the adoption of these criteria ensured that, in peripheral CF blood analyses, the primary biological variable under investigation was the presence of the CF disease, rather than treatment-related immunological perturbations.

Similarly, the non-CF **lung** cohort and the Healthy Donor (HD) **blood** cohort **do not originate from the same individuals.** Non-CF patients undergoing lung transplantation, as in pwCF, are exposed to intensive immunosuppressive regimens both before and immediately after surgery precluding the collection of untreated peripheral blood. Therefore, for the "non-CF" comparator blood cohort we exclusively used peripheral blood samples from untreated healthy volunteer donors, selected to minimize confounding factors related to ongoing therapies or underlying clinical conditions. This approach was adopted to ensure that comparisons between CF blood and HD blood reflected disease-associated differences rather than clinical or treatment-related heterogeneity.

We have now clarified these aspects in the Methods section (Study design) and supplementary tables by explicitly describing cohort composition and exclusion criteria, thereby eliminating potential ambiguities regarding cohort overlap and study design.

> iv. Why does the CF blood cohort lack any microbial data? We need this information to support interpretation of the data. This point is especially important given that the authors highlight a compartmentalised lung effect when comparing CF lung with CF blood; however, the observed differences may also reflect variation in infection status rather than purely tissue-specific effects.

To address this concern and strengthen data interpretation, we have now included detailed microbiological information for each CF blood sample at the time of collection, specifying infection status (including *Pseudomonas aeruginosa* and other relevant pathogens) in the new Supplementary Table 5.

We also performed stratified analyses correlating the frequency of all T cell subsets with infection status, both within CF lung and CF blood cohorts, and in relation to disease severity (FEV_1pp). These analyses are presented in the new Extended Data Fig. 2, which is entirely dedicated to these stratifications.

Importantly, these results demonstrate that while infection status contributes to variation in Th1/17$^+$ and pTh17 frequencies within the CF lung, it does not affect their peripheral blood counterparts, thus confirming that the observed differences between CF lung and CF blood primarily reflect tissue-specific effects. The inclusion of these dedicated supplementary figures now allows the readers to directly assess these relationships.

> v. It would also be valuable for the authors to explain why the stricter exclusion criteria used for the CF blood cohort were not applied consistently across all groups, and to discuss any implications this may have for interpreting the findings.
> vi. A potential recommendation to better profile the cohorts would perhaps be an additional supplementary table listing individual patients and their characteristics. This would be highly informative for clinically focused readers and would provide helpful context for interpreting variability across cohorts.

v. As outlined in our responses to the previous comments, the stricter exclusion criteria applied to the CF blood cohort were driven by ethical and clinical considerations, rather than by possible inconsistencies in study design.

vi. We have now added three Supplementary Tables (suppl. Tables 3–5) providing detailed individual-level information for each patient, including key clinical characteristics and microbiological status at the time of sampling. In addition, we performed comprehensive stratified analyses of clinical, microbiological, and immunological parameters and included these results in two dedicated Figures (Supplementary Figure 1 and Extended Data Fig. 2).
We trust that these additions can fully satisfy the reviewer's concerns and that they will facilitate the interpretation of inter-individual variability across cohorts.

> vii. Finally, in line with the above, given the use of explanted tissues, there is an inherent bias toward advanced CF disease. How do the authors think disease severity might have influenced their findings and generalisability? Would similar effects be expected in mild or moderate disease, and how might this impact the proposed use of Th1/17+ and pTh17 subsets as alternative targets for CF immune therapies aimed at restoring immune homeostasis?

We fully agree with the reviewer that the use of explanted lung tissue introduces an inherent bias toward advanced CF disease, and we appreciate the opportunity to clarify how disease severity may influence interpretation and generalizability of our findings.

Access to human CF lung tissue is, by necessity, restricted to the time of lung transplantation, and therefore reflects end-stage disease. Indeed, it is not ethically or clinically feasible to obtain lung samples from patients with mild or moderate CF disease, and this represents a well-recognized limitation of human CF lung immunology studies.
To explore potential alternatives, we previously evaluated sputum-derived immune cells; however, these samples predominantly reflect the upper airways and are overwhelmingly enriched in innate immune cells—neutrophils, macrophages, and dendritic cells—with lymphocytes representing only ~1–2% of total immune cells. A representative gating strategy of CF-sputum sample is shown in the Figure provided below. As such, sputum samples provide insufficient T cell numbers to support efficient cell-sorting, which requires at least 5–10 x 10^6 total lymphocytes to obtain adequate numbers of Th17 cell subsets for subsequent transcriptomic and functional analysis. Therefore, lung tissue remains indispensable as the only suitable source for accurate immunophenotyping and subsequent functional analyses.

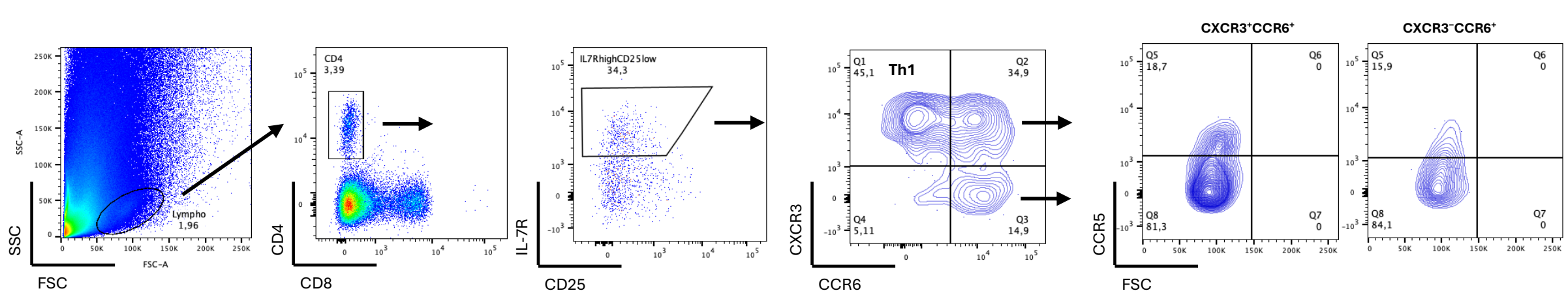

Moreover, while immunophenotypic analysis of sputum samples may provide a partial indication of Th17 subset distribution at intermediate stages of disease, these samples do not fully capture the immunological landscape of the lung. Importantly, addressing this aspect is beyond the primary scope of the present study, which is focused on defining the mechanisms underlying *P. aeruginosa*-dependent Th17 cell reprogramming within the CF lung environment.
Importantly, although our ex vivo analyses capture immune landscapes at advanced disease stages, the functional and mechanistic insights obtained from complementary in vitro systems strongly suggest that our observations are relevant beyond end-stage disease. Specifically, using air–liquid interface (ALI) cultures of primary human airway epithelial cells and antigen-specific stimulation assays, we demonstrate that early clinical isolates of *P. aeruginosa* are already able to drive the differentiation of protective *peripheral* cTh17 cells toward IFNγ-producing Th17 subsets. This result indicates that the emergence of Th1/17^{+} and pTh17 cells is not merely a late consequence of terminal disease but likely

begins during early infection and disease progression. Consistently, ALI experiments show that IFNγ-producing Th17 cells can directly induce epithelial damage, supporting a causal role for these subsets in lung pathology. Collectively, these data suggest that the accumulation of Th1/17^{+} and pTh17 cells observed in advanced CF lungs reflects amplification of pathogenic mechanisms rather than phenomena unique to end-stage disease.
From a therapeutic perspective, our results support the concept that selectively targeting IFNγ-producing Th17 subsets—while sparing protective cTh17 responses against *P. aeruginosa*—may represent a promising strategy to limit immune-mediated lung damage across disease stages. While longitudinal validation in CF patients with mild or moderate disease is not currently feasible at the tissue level, our data provide a strong mechanistic rationale supporting the relevance of these pathways beyond end-stage CF.
We have now expanded the Discussion to explicitly address disease severity, generalizability, and therapeutic implications, and to clearly acknowledge both the limitations and the broader translational significance of our findings.

viii. A growing challenge in both clinical management of CF and CF research is the classification of chronic infection, particularly following the introduction of highly effective modulator therapy. It would be helpful if the authors could clarify how chronic infection was defined in this study, including any specific criteria used. Additionally, please provide details on the proportion of patients with Pseudomonas aeruginosa in isolation compared to those with polymicrobial cultures. The table suggests that from 19 patients, 28 pathogens (including AF) were identified, could the authors therefore provide further details since their analysis would suggest a more binary classification of Pa positive in isolation vs other pathogens rather than polymicrobial cultures.

We agree with the reviewer that the precise definition of chronic infection is an important point, particularly in the light of the increasing complexity of defining chronic infection in CF in the era of highly effective CFTR modulators.

In our study *chronic infection* was defined according to the European CF Society Patient Registry (ECFSPR), which defines "*chronic infection*" when >50% of respiratory samples collected during the last 12 months preceding the last reported culture are positive for the pathogen under consideration.
It is important to note that the Italian CF Registry (RIFC) applies slightly different definitions: RIFC assigns *chronic infection* when a given pathogen is isolated in ≥3 respiratory cultures over the reference year. While these definitions largely overlap, minor differences can create discrepancies in patient classification. Moreover, epidemiological data from the RIFC indicate that, among adult Italian pwCF, the most prevalent chronic airway infections are due to *P. aeruginosa* (~36%) and *S. aureus* (~34%). However, RIFC data do not specify whether these infections occur as isolated monomicrobial infections or as part of polymicrobial colonization.
In our cohort, *P. aeruginosa* was consistently and persistently detected in pwCF classified as Pa^{+}, fully meeting both ECFSPR and RIFC criteria for chronic infection. In contrast, the detection of *S. aureus* was heterogeneous and frequently fell at the threshold of chronicity. In several cases, *S. aureus* was isolated in 2–3, up to 5 respiratory cultures, sometimes non-consecutively, making the classification of chronic versus intermittent infection inherently ambiguous. Consequently, in our opinion, stratification based primarily on *S. aureus* would be less robust and potentially misleading.
Nevertheless, to maintain methodological consistency and comparability, we have now stratified *S. aureus* chronic infection according to ECFSPR criteria and included these data in the Supplementary Tables 3-5 to explicitly report the full spectrum of pathogens detected in each patient.
As requested by the reviewer, the definition of *chronic infection* is now explicitly reported in the Supplementary Tables, where microbial status for each CF patient in both lung and blood cohorts is individually stratified, including the presence of *S. aureus* (MSSA vs MRSA) and other intermittently detected pathogens, providing full transparency regarding microbial complexity.

Moreover, to further clarify microbial status, we have also included a new Extended Data Fig.2 specifically focused to microbial stratification, in which the percentage of T-cell subsets in CF lung or CF blood samples are stratified according to the combined presence of *P. aeruginosa* and/or *S. aureus*. Even upon this further stratification, our data confirm that Th1/17^{+} cells were significantly enriched in the Pa^{+} group compared to Pa^{-} patients, irrespective of *S. aureus* co-detection. Similarly, lung-resident pTh17 cells were largely restricted to Pa^{+} samples, regardless of *S. aureus* status, with the exception of a single CF patient (Extended Data Fig.2a).
Although this pattern indicates a predominant association between these pathogenic Th17 subsets and *P. aeruginosa*, further stratification by combined pathogen status yielded very small group sizes (n=1–2 in some groups), limiting statistical power and precluding robust conclusions. Therefore, the primary stratification in the study remained Pa^{+} versus Pa^{-}, which captures the dominant immunological effect of *P. aeruginosa* while maintaining sufficient sample size for meaningful analysis.

Overall, we find that our approach—primary stratification by chronic *P. aeruginosa* infection combined with explicit reporting of additional microbiological findings—provides a biologically and clinically meaningful framework for interpreting immune phenotypes in CF lungs, while fully acknowledging the complexity of individual patient microbiomes.

ix. It would help the reader if the section headers included CF or non-CF

We thank the reviewer for this helpful suggestion.

For all immunophenotyping analyses comparing Th17 subsets between CF and non-CF cohorts—including RNA-seq and TCR clonotyping—the cohort identity is already explicitly indicated in the section titles and figure legends, ensuring that readers can immediately identify the relevant group.
In contrast, for the experiments involving the screening of the large panel of clinical *P. aeruginosa* strains (26 clinical isolates plus the reference strain PAO1) on human dendritic cells and subsequent Th17 differentiation, it was not feasible to perform these assays directly on cells from pwCF. Due to ethical considerations and limitations on the volume of biological material that can be collected from pwCF for diagnostic purposes, obtaining sufficient numbers of primary DCs for these large-scale screening was not possible. Consequently, these experiments were necessarily performed using cells isolated from blood of healthy donors. This point has now been explicitly indicated at the beginning of the Results section describing these screening experiments, as well as in the corresponding figure legends.
Instead, for experiments examining transcription factor activation during the transdifferentiation of cTh17 cells, we used cells from CF patients (as already reported in the figure legends), as these analyses involved a limited number of bacterial strains and required demonstrating activation patterns directly relevant to CF pathology. We have additionally specified in all figure legends whether CF- or HD-derived cells were used.

2. Figures (see more in minor, but taken together, this is an overarching issue, so flagged here)

a. In Fig 1a why are there such small n, given that similar data appear elsewhere to include the full group sizes?

A similar concern was also noted by Reviewer 3, and we fully agree that clarification of this aspect is important.
The smaller sample size in Figure 1a reflects the specific experimental purpose and technical requirements of this analysis, which differ from those of the subsequent immunophenotyping experiments shown elsewhere in the manuscript (e.g., Figures 2c-d, RNA-seq, TCR-seq).

Specifically, Fig. 1a shows intracellular cytokine staining for IFNγ and IL-17 following short-term PMA/ionomycin stimulation of total $CD4^+$ T cells, performed on blood samples from HD and CF patients, and on lung samples from CF and non-CF patients. This assay was designed as an exploratory functional readout to establish the presence and relative abundance of <u>$IFN\gamma^+/IL17^+$ co-producing $CD4^+$ T cells</u> across compartments. Importantly, this type of stimulation-based cytokine assay requires relatively modest cell numbers, as robust quantification of cytokine-producing cells can be achieved with fewer than 1×10^6 cells per sample.

In contrast, the subsequent immunophenotyping analyses—and particularly those involving cell-sorting experiments—were performed *ex vivo*, without PMA/ionomycin stimulation, and relied on multistep gating strategies based on the combined expression of multiple surface markers. These analyses require substantially larger numbers of cells (typically ≥5–10 × 10^6 $CD4^+$ T cells per sample) to ensure adequate representation of rare subsets and to obtain sufficient numbers of Th cell populations for sorting. Therefore, not all CF and non-CF lung samples yielded sufficient cell numbers to be included in <u>**both**</u> the cytokine assays <u>**and**</u> the more extensive immunophenotyping experiments. Some samples were consequently prioritized for cell sorting and transcriptomic analyses, which have higher cell number requirements.

Thus, the sample size in Fig. 1a reflects objective technical constraints and the distinct cellular requirements of the experimental approaches, rather than selective sample exclusion.

Specifically, samples yielding insufficient numbers of lymphocytes and/or total $CD4^+$ T cells were excluded because the resulting numbers of $IFN\gamma^+$ and/or $IL\text{-}17^+$ single- and double-positive cells were too low to be biologically meaningful, as they were calculated from fewer than 2,000 total $CD4^+$ T-cell events (see example in figure below showing one excluded sample).

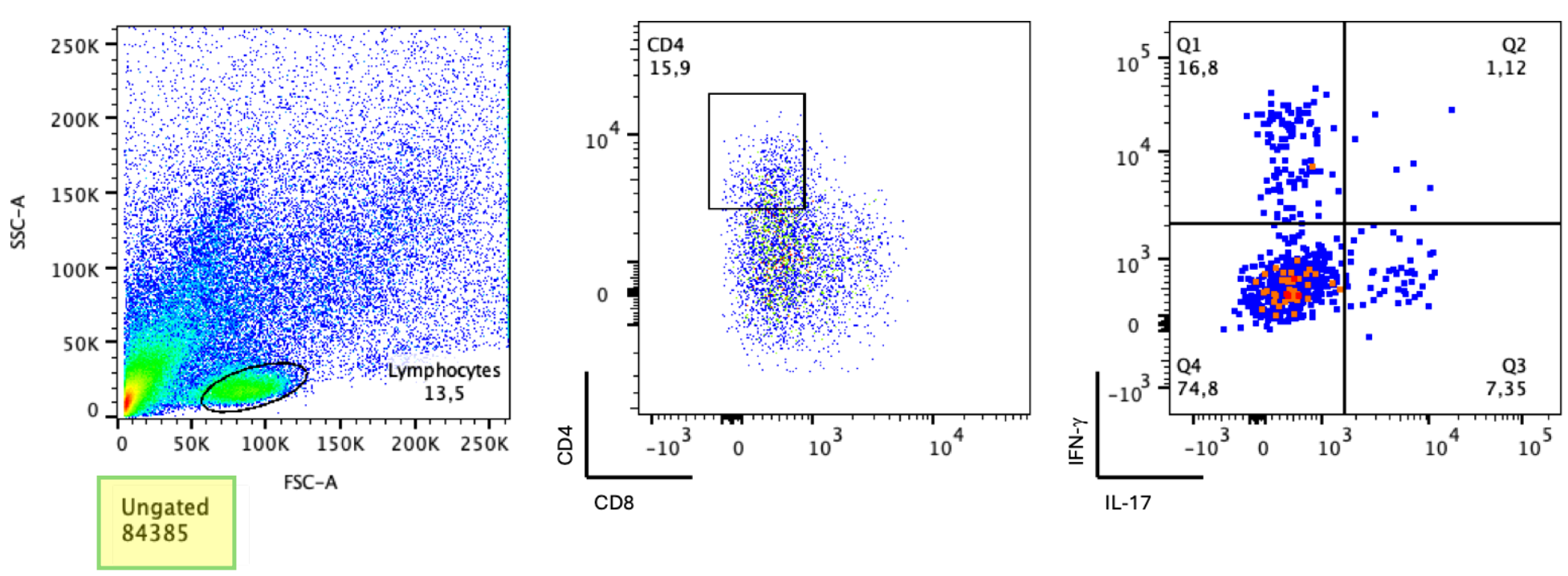

To directly address the reviewer's concern, we have now expanded Fig. 1a to include 4 additional blood samples (2 CF and 2HD), as well as <u>3 CF lung</u> and <u>1 non-CF lung</u> samples, that were initially excluded due to insufficient cells to support <u>***both***</u> cytokine secretion assays <u>***and***</u> comprehensive immunophenotyping. For all these samples, which yielded sufficient lymphocytes and $CD4^+$ T cells to allow reliable analysis of cytokine production only, <u>all available cells were used exclusively for cytokine secretion assays and could not be allocated to parallel immunophenotyping experiments</u>.

The inclusion of these additional samples in our analysis did not affect the overall results.

b. Fig 1b: please review the legend and make this figure more understandable and add details, eg CF/ non-CF; describe what the reader should be expected to take away from the heatmap. Same for other potentially non-intuitive figs throughout.

We have now revised the figure legends for Fig. 1b and the corresponding Extended Data Fig. 1b associated with the CRUSTY-based clustering analysis to improve clarity and accessibility for the reader. Specifically, the legends now explicitly indicate whether samples derive from CF or non-CF patients and clearly describe the biological question addressed by the clustering.

In addition, we have expanded the legend text to guide the reader through the interpretation of the heatmap, explaining what each axis represents, how the clustering was performed using the CRUSTY software, and what the key take-home message of the analysis is—namely, the differential organization of T cell -related clusters between CF and non-CF samples.
Similar clarifications have been applied to other figures involving non-intuitive analytical approaches throughout the manuscript, with the aim of making the data more easily interpretable without altering the underlying analyses.

3. Analysis:

a. The term 'correlate' is used quite extensively; in some cases, the authors mean 'two groups separated on the basis of a feature (eg Pa+/-) had statistically different values of something. I think 'associated with' would be more accurate. In other places there seem to have been multiple 'correlations' explored, without details of which test was used and no mention of whether any adjustment has been made for the multiple comparisons.

We agree that in several instances the term "correlate(s) with" was used to describe statistically significant differences between groups stratified by a categorical feature (e.g. *P. aeruginosa*–positive versus –negative). In these cases, no formal correlation analysis was performed, and we agree that the term "associated with" more accurately reflects the nature of these comparisons. We have therefore revised the manuscript accordingly, replacing "correlate(s)" with "is/are associated with" wherever categorical group comparisons were conducted.
In contrast, in other parts of the study we explicitly tested correlations between continuous variables, such as inflammatory response and quantitative microbiological features of *P. aeruginosa* (Figures 3–4 and related Extended Data Figures 2, 6, 7). In these cases, formal correlation analyses were performed using Pearson correlation tests, as explicitly indicated in the figure legends. We have now further clarified this point in the Methods section, specifying that Pearson parametric correlation was used for statistical analyses involving continuous variables.

4. Conclusions: largely well-written, but the authors have not included their own evaluation of their study having not included any details of potential strengths and more importantly limitations to their study. This I believe is an important part of any discussion and should be addressed by the authors.

We thank the reviewer for this important and constructive comment. We agree that an explicit evaluation of the strengths and limitations of the study is essential to contextualize our findings. Accordingly, we have revised the Discussion to explicitly address the main strengths and limitations of our work throughout the text (*all changes are highlighted in red in the revised manuscript*). Specifically, we discuss the use of human CF lung explants, the cohort size constraints inherent to such studies, the focus on advanced-stage disease, and the current lack of identification of the specific antigens driving pathogenic Th17 skewing. At the same time, we highlight how the integration of *ex vivo* human data with mechanistic *in vitro* approaches strengthens the robustness of our conclusions. All newly added text has been highlighted in red for ease of reference.

Minor issues:

1. The authors have appropriately addressed technical variation through the use of ComBat-seq. However, it is unclear whether similar measures were taken to account for donor-related variability (e.g., age, disease severity, treatment history). Clarification on whether steps were

implemented to mitigate these potential confounders would be important especially given the limited number if replicates per group.

We thank the Reviewer for this suggestion. We repeated the analysis including age, sex, and treatment as additional covariates and obtained results that were unchanged from our original findings.
This outcome is expected given our study design. In the original analysis, donor identity was used for batch-effect correction because our objective was to identify cell-type–specific signatures that are robust across individuals. Age, sex, and treatment are donor-level attributes and are therefore fully (or near-fully) confounded with donor identity.
Consequently, once donor-specific effects are accounted for, these covariates do not provide additional independent information, and their inclusion does not alter the results.
Moreover, given the limited number of biological replicates per condition (n = 3–4), the statistical power to separately estimate individual variability and additional donor-level covariate effects is inherently limited.
Larger cohorts would be required to robustly disentangle these effects, which represents an important direction for future studies. We note that the isolation and sorting of these specific cell populations is technically demanding, which constrained the achievable sample size. Nevertheless, the consistency of the results across donors supports the robustness of the identified cell-type signatures.

2. Can the authors clarify and justify the chosen co-culture duration for the P. aeruginosa infection assays of moDCs? The manuscript states (lines 173–174) that selective expansion of Th1/17+ and pTh17 cells occurs only in chronically infected CF lungs. However, the model used involves co-culturing cells with P. aeruginosa for 90 minutes, with a maximum readout at 24 hours. Is this timeframe sufficient to reflect chronic infection dynamics?

We thank the reviewer for this important comment, which highlights the distinction between the in vivo expansion of IFNγ^{+} Th17 cells observed in chronically infected CF lungs and the in vitro models used to investigate its underlying mechanisms.

The statement that selective expansion of Th1/17^{+} and pTh17 cells occurs in chronically infected CF lungs refers exclusively to our *ex vivo analyses* of explanted CF lung tissues (Fig. 1D), where chronic *P. aeruginosa* infection was defined according to established clinical microbiological criteria. These findings therefore reflect long-term immune remodelling occurring in vivo.

In contrast, the *in vitro experiments* involving *P. aeruginosa* were designed to address two distinct and complementary mechanistic questions, each with its own appropriate temporal framework:

1) Short-term *P. aeruginosa* infection assays in human primary DCs were designed to address mechanistic questions related to bacterial uptake, intracellular persistence, and inflammatory response (Fig. 3). The 90-minute infection followed by gentamicin protection and readouts up to 24 hours represents the standard and widely adopted approach to assess intracellular survival and cytokine induction in DCs. Importantly, 24 hours corresponds to the peak of DC cytokine production while preserving sufficient cell viability, particularly when comparing early and late clinical *P. aeruginosa* isolates with distinct virulence properties. Indeed, some early isolates induced substantial DC death already at 24 hours (Extended Data Fig. 5), and extending the incubation beyond this time point would preclude accurate assessment of the inflammatory response. Moreover, prolonged in vitro infection would not reliably model chronic infection dynamics, as primary DCs do not proliferate and progressively lose viability in culture. Longer experiments would also require repeated media replacement, preventing accurate evaluation of cumulative cytokine production in the culture supernatant. Thus, this assay is specifically optimized to quantify *P. aeruginosa* intracellular persistence and inflammatory response of infected-DCs, rather than to mimic chronic infection per se.

2) <u>The generation of pathogenic Th17 subsets</u> was instead evaluated in a separate long-term co-culture experimental setting, in which *P. aeruginosa*-infected DC were cultured with T-cells for up to 10 days, with media replacement every 48 hours to support T-cell survival, proliferation and differentiation (Figure 4).

This timeframe reflects our previously observed kinetics of antigen-driven human Th17 cell transdifferentiation, which typically peak between days 7 and 10. As shown in the figure below (representative of the experiments performed to establish this assay), at day 4 a substantial fraction of undivided cells is still present, which do not express the activation marker CD40L (right panel, “undivided”), together with a relatively low number of proliferating cells, of which approximately 40% are CD40L^{+}. By day 7, the proportion of proliferating cells increases, accompanied by a higher frequency of CD40L expression, reaching around 60%. At day 10, the initial peak of undivided cells is no longer detectable, while proliferating cells become the predominant population and display robust CD40L expression, approaching 80%. For this reason, we selected day 10 as the most appropriate time point for assessing pTh17 transdifferentiation

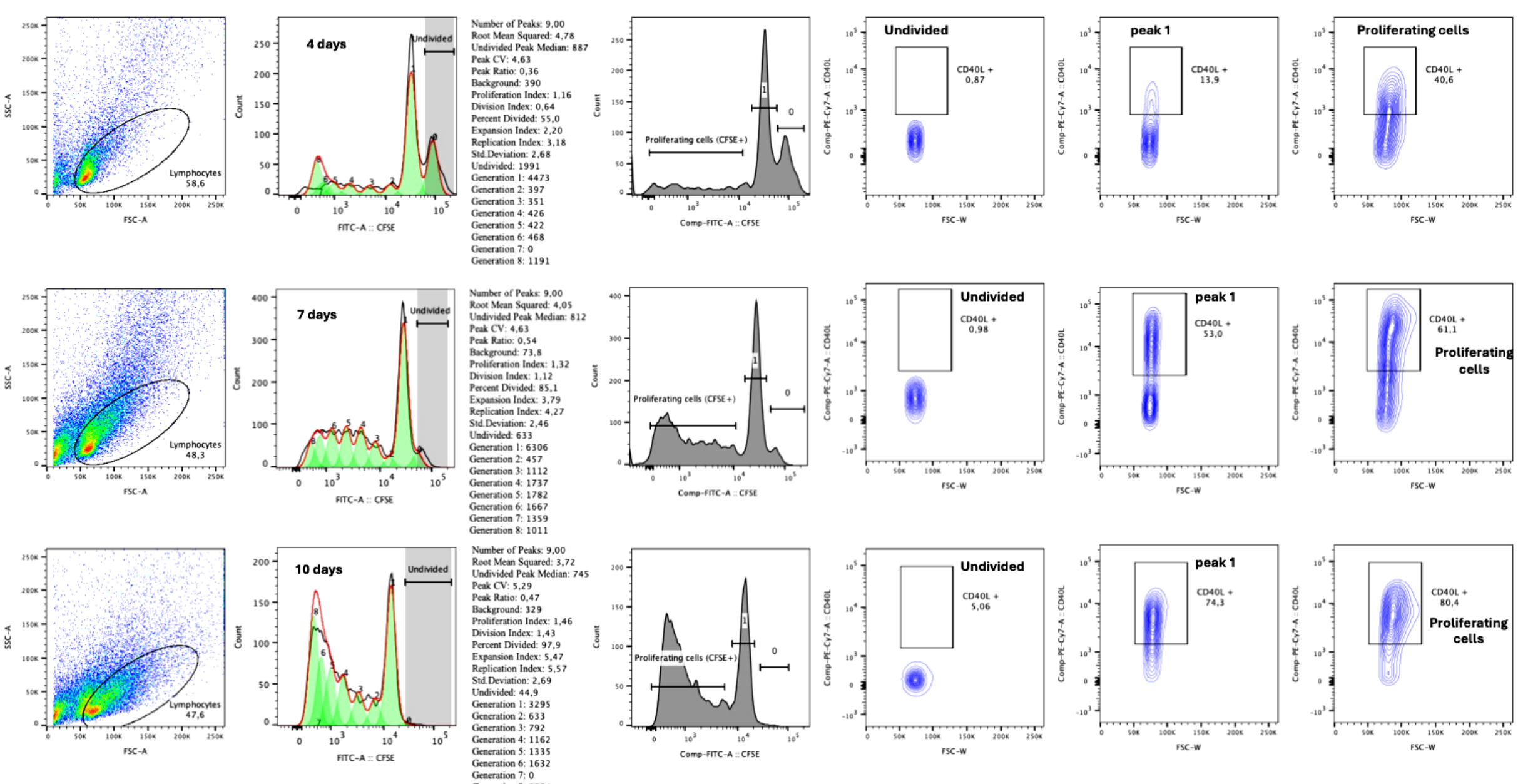

Together, these complementary approaches allow us to link *P. aeruginosa*–driven DC activation to subsequent pathogenic Th17 cell generation, without implying that the short-term in vitro infection model is intended to recapitulate the temporal complexity of chronic CF lung infection.

3. Could the authors clarify why TEER measurements were not included to assess barrier integrity alongside structural markers such as ZO-1?

We decided to do not include TEER measurements in the submitted version of the manuscript due to technical constraints inherent to the air–liquid interface (ALI) model. Accurate TEER measurements in air–liquid interface (ALI) cultures require the transient addition of culture medium to the apical compartment of Transwell to allow electrode contact, whereas in ALI conditions the apical part of epithelial layer is normally exposed to air throughout the entire experiment. This manipulation transiently disrupts ALI conditions and may introduce experimental perturbations to the epithelial layer, affecting epithelial morphology and tight-junction organization.

Dipartimento di Bioscienze
Via Celoria 26 – 20133 Milano, Italy
Tel 02503 15042 - Fax 02503 15044 – direzione.bioscienze@unimi.it

For this reason, TEER measurements were not performed on the same Transwell inserts used for immunofluorescence (ZO-1 staining) and cytokine quantification, in order to avoid experimental interference with structural and functional readouts.
Importantly, TEER measurements were performed during the experimental setup phase using independent Transwell inserts, cultured and treated under identical conditions. These measurements confirmed a significant reduction in barrier resistance in the presence of IFNγ^{+}Th17 subsets compared to cTh17 cells, supporting the structural alterations observed by ZO-1 staining.
We have reported these TEER values here for the reviewer's evaluation. However, given the potential perturbation of ALI conditions and the fact that TEER was not measured on the same inserts used for structural analyses, these data were not included in the manuscript to avoid overinterpretation.
In our view, TEER provides complementary functional confirmation of barrier impairment, whereas ZO-1 staining offers a more direct visualization of tight-junction disruption within the intact ALI system.

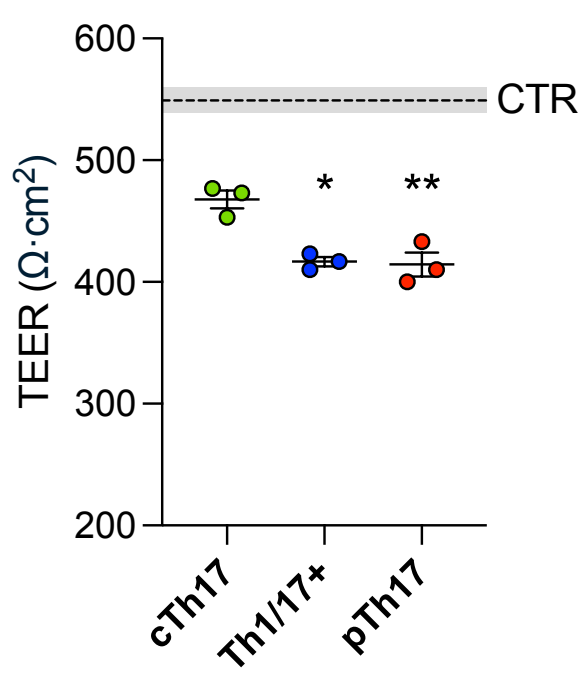

Statistical significance between epithelial cells untreated (CTR) or cultured in the presence of activated T-cell subsets was calculated using one-way ANOVA and reported as * $p < 0.05$, ** $p < 0.01$.

4. Figures: Please ensure figures and captions are looked at carefully, there are some areas that need closer attention and would benefit from being revised. Examples include 1D: the blue is easy, but then the use of the three shades of purple and mix of filled/ empty symbols with empty/ filled boxes is very confusing.

We agree with the reviewer that the original colour scheme and symbol used in Figure 1d is confusing, therefore we have revised Figure 1d and all related stratified analyses (new Supplementary Fig 1 and Extended Data Fig. 2) to ensure a clear and consistent graphical representation across the manuscript. Specifically, we simplified the colour palette to eliminate redundant shades, established a one-to-one correspondence between colours and experimental groups, and standardized the use of filled and open symbols/boxes to avoid ambiguity.
The figure legends have been updated accordingly to clearly define each visual element.
We believe these revisions substantially improve readability and facilitate accurate data interpretation.

5. Please can the authors review the figure captions. There appears to be some inconsistency with information provide, namely numbers per group/cohorts used in the figure or analysis. This appears to be included in some of the figure legends, yet it is missing from others. Please can the authors ensure consistency and provide these details.

We thank the reviewer for highlighting this important point.
Upon careful re-examination of all figure legends, we identified that although sample numbers were reported for several analyses, this information was not provided consistently across all figures and panels.
We have now systematically revised all figure legends, including both main and Extended Data Figures, to ensure uniform reporting of sample sizes. For each analysis, we explicitly define what n represents (e.g., n=number of samples, lung or blood samples, and the number of independent experiments). In

cases where sample numbers differ between panels or experimental conditions, these differences are now clearly specified in the corresponding legend.

6. Figures 2A and S2A/B: please could the authors expand the figure captions for the heatmaps in Figures 2a and S2a/b to provide sufficient contextual detail. According to the methodology, the analysis appears to include a small number of samples (CF blood: n = 3; CF lung: n = 4; non-CF lung: n = 4). Furthermore, it would be helpful if the authors could indicate what proportion of these samples were classified as chronic P. aeruginosa or not. If possible, it would be nice to add this information directly on the figures to assist interpretation.
In addition, as stated in lines 136–139, the authors note that when considering CF samples alone, there is a greater differentiation between CF blood and CF lung. However, this separation appears to arise primarily from the revised hierarchical clustering performed after excluding the non-CF group. In contrast, the original clustering including all groups suggests a lesser degree of separation, with CF blood samples clustering more closely with the non-CF lung group. This is an interesting observation; it would benefit the manuscript if the authors clarified this point more explicitly in the manuscript and corresponding figure legends.

We thank the reviewer for this helpful comment regarding the figure legends and the interpretation of the heatmaps.

The primary aim of our transcriptomic analyses was not to perform a global comparison of tissues (CF blood vs CF lung) or disease states (CF vs non-CF), but rather to focus on transcriptional signatures of specific pathogenic and protective T-cell subsets. Given the limited sample size (n = 3–4 per group), we deliberately adopted a cell subset–focused strategy to extract robust transcriptional signatures associated with pathogenic T-cell subsets while minimizing donor-driven variability.
We would like to clarify that we do not state that CF blood and CF lung samples show a greater separation at the clustering level when considering CF samples alone (Extended Data Fig. 2a and 2b). This CF-restricted analysis was an intentional analytical step aimed at disentangling disease-specific effects from compartment-specific transcriptional programs. By excluding the non-CF lung samples, we sought to directly compare pathogenic and protective T cell subsets within the same disease context and to assess compartment-dependent differences independently of inter-disease variability. Accordingly, the observed differences in Extended Data Fig. 4a-b reflect the direction and magnitude of differential gene expression within CF, rather than differences in sample clustering.
Specifically, the heatmaps in Extended Data Fig. 4a-b are based on distinct gene sets derived from separate analytical steps: Figure 2a shows a refined gene set defining a CF lung–specific pathogenic signature, whereas Extended Data Fig. 4a-b display DEGs derived from analyses restricted to CF blood or CF lung, respectively. Hierarchical clustering was applied only to genes and not to samples.
To enhance clarity and transparency, we have:
- expanded the figure legends of Figures 2a and Extended Data Fig. 4 to explicitly describe the analytical strategy, gene selection criteria, and clustering approach;
- Indicated sample sizes and infection status (all CF samples were from patients with chronic *P. aeruginosa* infection);
- Provided complete lists of DEGs for all comparisons in the supplementary material (**Supplementary data**), now referenced in the Results section.
We hope that these revisions clarify the analytical framework and improve the interpretability of the heatmaps.

7. The manuscript, whilst well written, can at times be difficult to follow, due to the number of analytic approaches incorporated and often assumed understanding. Efforts should therefore be made to improve readability and accessibility for wider audiences with perhaps less of a fundamental science background.

Dipartimento di Bioscienze
Via Celoria 26 – 20133 Milano, Italy
Tel 02503 15042 - Fax 02503 15044 – direzione.bioscienze@unimi.it

We thank the reviewer for this constructive comment.

To improve readability and accessibility for a broader audience, we have carefully revised Results section (with all additions highlighted in red in the revised manuscript) to:
- introduce clearer transitional sentences explaining the rationale underlying each experimental approach;
- explicitly link ex vivo observations in CF lungs with the subsequent mechanistic in vitro and functional assays;
- simplify sentence structure and streamline overly technical passages to enhance narrative flow without reducing scientific rigor.

Importantly, we have added concise guiding statements at the beginning of Results sections to clarify the biological question being addressed and how each experiment builds upon the previous one.
We believe these revisions substantially improve clarity and accessibility while preserving the depth and integrity of the work.

8. Line 88-90: "Our findings highlight Th1/17+ and pTh17 subsets, and the immunological processes upstream of their generation, as promising targets for novel and more selective immunomodulatory therapies in CF, offering a potential alternative to current broad-spectrum anti-inflammatory approaches." This sentence requires more cautious interpretation. While the data supports the first part of the statement, the latter part was not the primary aim of the study and is therefore speculative. Please revise to clearly indicate that this is a putative interpretation rather than a conclusion drawn from the study.

We agree that while our data support the identification of Th1/17^{+} and pTh17 subsets as relevant contributors to CF immunopathology, the therapeutic implications remain speculative and were not directly tested in this study. Accordingly, we have revised the sentence in the manuscript to make this interpretation more cautious and explicit. The revised sentence now reads:
" Collectively, our findings identify Th1/17^{+} and pTh17 subsets, and the immunological processes that drive their generation, as key components of CF lung immunopathology and suggest that these pathways may represent potential targets for more selective immunomodulatory strategies."
This revision clarifies that the therapeutic implications represent a hypothesis-generating interpretation rather than a conclusion supported by direct experimental evidence.

9. Line 84: TCR repertoire – Please ensure use of 'T-cell receptor repertoire' ahead of abbreviation.

We added "T-cell receptor (TCR) repertoire" at first mention in the text.

10. Table S2: Please can the authors clarify what is meant by the one patient listed in the non-CF lung group with CF related diabetes. This is likely reflecting either a typo or mislabelling of a non-CF lung patient with diabetes as CFRD.

We apologize for this typographical error. During preparation of Supplementary Tables, "CFRD" was inadvertently listed for one patient in the non-CF lung group. The entry should have referred simply to diabetes mellitus, unrelated to CFTR mutations. This has now been corrected in the revised Supplementary Table 4.

Reviewer #2:

In this paper by Dusetti et al, the authors identify two pathogenic Th17 subsets—Th1/17$^+$ and pTh17 cells which are selectively enriched in CF lungs specially colonized by Pseudomonas aeruginosa. The authors show that these pathogenic IFNγ+Th17 subsets are derived from cTh17 precursors. Using ALI cultures, the authors show that upon activation they drive disruption of the pulmonary epithelial integrity and trigger exaggerated cytokine and chemokine responses. Using bulk RNA seq of sorted populations from CF and non-CF samples, transcriptomic and TCR profiling reveals functional details regarding the clonal selection of Th1/17$^+$ and pTh17 cells in CF lungs. This to me, is the most exciting part of the paper and the most novel- while there have been projections that Th17 and IL-17 have a role to play in CF pathology, the current study very elegantly using human samples from blood and lung to make this connection in a mechanistic way.
In the second part of the paper, they try and bridge the observation from early figs using human samples to using in vitro models – for examples using clinical P. aeruginosa strains that persist within dendritic cells they show that this induces IL-1β/IL-23 cytokine in DCs and promotes IFNγ-producing Th17 differentiation over classical Th1 lineage commitment – this is not especially surprising. It is however interesting that early isolates that probably lack lacking chronic adaptation modules can still potently reprogram cTh17 cells.

Major points:

1. The link between CF, Pseudomonas infection, and Th17 skewed inflammation is very nicely established, giving the work very high conceptual novelty.
The integration of lung vs blood comparisons, transcriptomics, and TCR clonotyping anchored to clinically relevant P. aeruginosa strains fits well- this is extremely difficult to do and I applaud the authors for their courage in sorting these population to carry out this difficult work.

We sincerely thank the reviewer for this generous and thoughtful assessment of our work.
We greatly appreciate the recognition of the conceptual novelty of our study and the challenges involved in integrating lung and blood analyses with transcriptomics and TCR clonotyping, together with ex vivo reprogramming of pathogenic Th17 cells using clinically characterized *P. aeruginosa* isolates.
We are particularly grateful for the acknowledgment of the technical challenges involved in isolating and characterizing these rare T-cell subsets from human lung tissue.

2. The additional mechanistic studies in vitro while appealing and add some mechanistic angle is not entirely novel. I would keep the focus on the early data. I have a few suggestions to improve some of the elements of the paper:

- Can the ZO-1 staining and barrier integrity be quantitated and included- as it stands, unless you are an expert you wouldn't know what to look for- also add some highlights within the figures to guide the reader's eye.

We thank the reviewer for this valuable suggestion.
To enhance the interpretability and objectivity of the immunofluorescence analyses, we have now performed quantitative image analysis of ZO-1 staining using AI-assisted image analysis (Arivis Pro v.4.4 software). For each field, the maximum projections of the image stacks were generated, and segmentation masks were created using the AI-assisted Draw Object Tool, which automatically detects structures within selected regions. The software calculated both the total segmented area and the relative volume normalized to the total fields, providing an objective estimate of junctional continuity, structural integrity, and spatial representation in the 2D projection.

The quantitative analysis was specifically designed to assess junctional discontinuity and to capture areas of disruption and ZO-1-negative regions, which reflect epithelial barrier damage."

These analyses are now reported in the new Extended Data Figure 3 dedicated to quantification of epithelial damage. In addition, to facilitate interpretation and guide the reader's eye, arrows have been added to the main Figure 1e highlighting areas of junctional disruption and ZO-1-negative regions within the epithelial layer.

2. For any of the readouts from CF patients can they look for correlation between their transcriptomics modules with clinical features such as lung function or exacerbation frequency?

We thank the reviewer for this insightful suggestion.

To explore potential associations between transcriptomic modules and clinical parameters, we computed a per-sample signature Z-score based on the compartment-specific DEGs and assessed their relationships with FEV_1pp and exacerbation frequency across CF-derived cell subsets both in blood and lung. These analyses are now reported in the new Extended Data Figure 4c.

Although this analysis recapitulated compartment-specific transcriptional signature, stratification according to the clinical variables examined did not reveal consistent associations. Given the limited sample size and clinical heterogeneity of the cohort, we consider this analysis exploratory and not sufficiently powered to draw robust conclusions regarding clinical correlations. We anticipate that larger, well-characterised cohorts will be required to more reliably evaluate the relationship between these transcriptional signatures and clinical outcomes.

We have included a description of the signature score calculation in the Methods and added a statement in the Results section to clarify these findings.

3. They make very strong interpretations and conclusions- since these are ex vivo analysed cell types, they should temper it, so it is a well-balanced read.

We acknowledge that some of the original language could be interpreted as overstating causality based on ex vivo and in vitro analyses. We have revised both the Results and Discussion sections to temper conclusions, using more cautious phrasing such as "may contribute," "could represent," or "suggests a potential role.".

We also clarified that while our data support a role for $Th1/17^+$ and pTh17 cells in CF lung immunopathology, further in vivo studies are needed to establish causality.

We believe that these revisions ensure a more balanced interpretation of our results, accurately reflecting the scope of our *ex vivo/in vitro* findings while preserving the scientific insights.

Reviewer #3:

Dusetti et al. examined Th17 cells in CF lungs and, using flow cytometry and scRNA-seq, identified distinct Th17 subsets. Two of these subsets are enriched and clonally expanded in CF patients, as demonstrated by single-cell V(D)J sequencing. Functionally, these cells disrupt epithelial integrity in co-culture with air–liquid interface epithelial cells and drive epithelial production of pro-inflammatory cytokines and chemokines. The authors further show that clinical P. aeruginosa isolates infect dendritic cells, inducing IL-1β and IL-23, which could underlie the in vivo generation of pathogenic Th17 cells. In an in vitro human Th17 culture system, these clinical isolates skew protective cTh17 cells toward pathogenic IFNγ-producing Th17 subsets. Overall, the data are novel and have the potential to reveal new targets for more selective immunomodulatory therapies in CF.
However, the manuscript could be improved in the following aspects:

1. Nomenclature. The Th17 nomenclature is confusing. According to Figure S1A, the authors focus on IFNγ$^+$IL-17$^+$ cells termed "Th1/17$^+$," CCR5$^+$IL-17$^+$ cells termed "pTh17" (it is unclear what "p" stands for), and CCR5$^-$IL-17$^+$ cells termed conventional Th17 cells. Clearer definitions, justification for these terms, and consistent use throughout the manuscript would help readers follow the subsets being discussed.

We thank the reviewer for raising this point and for the opportunity to clarify the nomenclature of the Th17 subsets analyzed in this study.

The terminology used here derives from the established literature and from our previous works characterizing human Th17 heterogeneity in chronic inflammatory diseases. In particular, IFNγ-producing Th17 cells are widely referred to as "Th1/17" cells, as they display an intermediate phenotype between classical Th1 and conventional Th17 cells. Specifically, Th1/17 cells co-produce the Th1- and Th17-signature cytokines IFNγ and IL-17, co-express the surface markers CXCR3 and CCR6, and express the lineage-defining transcription factors T-bet and RORγt, thus representing an intermediate functional and transcriptional state. In the literature, IFNγ-producing Th17 cells have been extensively described as one of the major pathogenic CD4$^+$ T-cell populations involved in several autoimmune and chronic inflammatory diseases.
In this context, we previously demonstrated that within the Th1/17 (CXCR3$^+$CCR6$^+$) compartment, the expression of the homing and activation marker CCR5 further discriminates functionally distinct subsets displaying different activation profiles and antigen specificities (Paroni M. et al., J Allergy Clin Immunol. 2017). Analogously, within the Th17 (CXCR3$^-$CCR6$^+$) compartment, CCR5 expression identifies two biologically divergent populations. CCR5$^-$ Th17 cells co-produce IL-17 and the regulatory cytokine IL-10 but not IFNγ and closely resemble the protective or "conventional" Th17 cells described in multiple murine models of chronic inflammation. We therefore refer to these cells – here and in our previous work – as conventional Th17 (cTh17). In contrast, CCR5$^+$ Th17 cells co-produce the *highest levels* of IFNγ and IL-17 among all CD4+ T-cell subsets, lack IL-10 expression, and display a distinct transcriptional and functional profile. Notably, this phenotype closely resembles the pathogenic Th17 subset described in murine models of chronic inflammatory disease. Based on these functional, transcriptional, and antigen-specific properties, we previously defined these cells as pro-inflammatory Th17 cells (Paroni M. et al., JCC 2023) and continue to refer to them in the present manuscript as pTh17 (pro-inflammatory Th17 cells).
To improve clarity for readers, we have now explicitly defined each subset, including marker combinations and abbreviations, in the Methods section and in all relevant figure legends, and ensured consistent terminology throughout the manuscript.

2. Sample size in Figure 1. It is not clear why only 3–4 patients per group are shown in Figure 1 when 19 CF patients were recruited. The authors should either include data from all available patients or explain the criteria for inclusion/exclusion and whether the shown cohort is representative of the full study population.

We thank the reviewer for raising this point. A similar concern was also noted by Reviewer 1, and we fully agree that clarification of this aspect is important.

The smaller sample size in Figure 1a reflects the specific experimental purpose and technical requirements of this analysis, which differ from those of the subsequent immunophenotyping experiments shown elsewhere in the manuscript (e.g., Figs. 2C/D, RNA-seq, TCR-seq).
In particular, in Figure 1a intracellular cytokine staining for IFNγ and IL-17 was performed after short-term polyclonal (PMA/ionomycin) stimulation of total $CD4^+$ T cells obtained from blood (HD and CF) and lung samples (CF and non-CF). This experiment was conceived as an initial functional assessment aimed at determining whether $IFN\gamma^+IL\text{-}17^+$ co-producing $CD4^+$ T cells could be detected and differ across the different compartments analyzed. Importantly, this type of stimulation-based cytokine assay requires relatively low cell numbers, as robust quantification of cytokine-producing cells can be achieved with fewer than 1×10^6 cells per sample.
By contrast, the downstream immunophenotyping experiments—and especially those involving cell sorting—were conducted ex vivo without PMA/ionomycin stimulation and required multistep gating strategies based on multiple surface markers. These analyses demand substantially larger cell inputs (generally ≥5–10×10^6 $CD4^+$ T cells per sample) to ensure adequate representation of rare subsets and to recover sufficient numbers of Th17 populations for sorting and downstream transcriptomic analyses. As a consequence, not all lung samples from CF and especially from non-CF donors yielded enough cells to support **both** the cytokine assay **and** the more extensive immunophenotyping workflow. In several cases, available samples were therefore prioritized for sorting and transcriptomic profiling, which impose stricter cell number requirements.
Accordingly, the number of samples shown in Figure 1a reflects technical and biological constraints related to cell yield rather than any selective exclusion of samples. More specifically, samples yielding insufficient numbers of lymphocytes and/or total $CD4^+$ T cells were excluded because the resulting numbers of $IFN\gamma^+$ and/or $IL\text{-}17^+$ single- and double-positive cells were too low to be biologically meaningful, as they were calculated from fewer than 2,000 total $CD4^+$ T-cell events (see example in figure below showing one excluded sample).

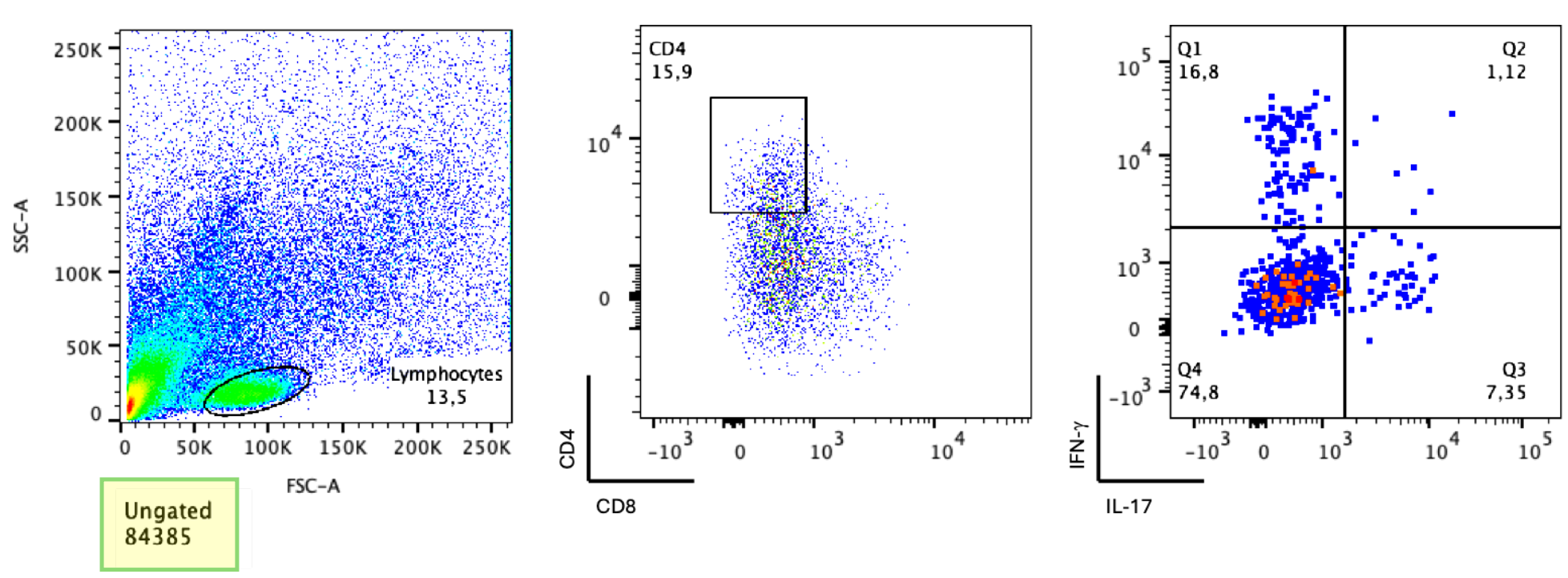

To directly address the reviewer's concern, we have now expanded Figure 1a to include additional 4 blood samples (2 CF and 2HD), as well as 3 CF lung and 1 non-CF lung samples, that were initially excluded due to insufficient cells to support ***both*** cytokine secretion assays ***and*** comprehensive immunophenotyping. For all these samples, which yielded sufficient lymphocytes and $CD4^+$ T cells to

allow reliable analysis of cytokine production only, all available cells were used exclusively for cytokine secretion assays and could not be allocated to parallel immunophenotyping experiments.

> 3. Clonality and microbial status. In Figure 2, the clonality analysis does not appear to account for Pseudomonas status. Are the expanded clones predominantly found in P. aeruginosa–colonized lungs, or do other bacterial species also drive similar clonal expansions? Stratifying the clonal data by microbial colonization (e.g., P. aeruginosa vs. other pathogens or polymicrobial states) would substantially strengthen the interpretation.

We thank the reviewer for this important point regarding the relationship between TCR clonality and microbial colonization status.

To minimize potential confounding variables, the samples used for **both** RNA-seq **and** TCR sequencing analyses come from the same sorted populations and were deliberately selected from CF patients with chronic *Pseudomonas aeruginosa* lung infection.
This approach was motivated by two main reasons:
- First, our immunophenotyping data revealed that lungs chronically colonized by *P. aeruginosa* typically yielded higher numbers of IFNγ^{+}Th17 cell subsets (**Figure 1d, new Extended Data Figure 2a**), which facilitates the sorting of sufficient cell number for downstream transcriptomic and TCR analyses.
- Second, restricting the cohort to *P. aeruginosa*-positive patients allowed us to avoid introducing additional microbiological heterogeneity that could confound the interpretation of transcriptional and clonal expansion patterns.

To improve transparency regarding the microbial status of the study population, we have now expanded the Supplementary Table 2 into three separate, more detailed Supplementary tables corresponding to CF blood, CF lung, and non-CF lung cohorts. These tables now explicitly report, for each patient, the presence of chronic lung infection with *P. aeruginosa* and/or *Staphylococcus aureus*, as well as the occurrence of other pathogens detected as intermittent infections.
As shown in these tables and given the limited number of lung samples analyzed for TCR clonotypes, further stratification based on polymicrobial colonization would not be statistically meaningful. Importantly, the deliberate selection of *P. aeruginosa*–colonized CF lungs for the transcriptomic and clonotype analyses was intended precisely to strengthen the biological interpretation of the data by reducing microbiological variability.
We have now clarified this point in the revised manuscript and in the description of the patient cohorts.

> 4. Mechanistic role of dendritic cells. In Figure 5, additional mechanistic insight into dendritic cell responses would be valuable. Beyond showing that clinical isolates can induce IFNγ^{+}IL-17^{+} cells, it would be informative to examine how these isolates reprogram DCs at the molecular level (e.g., transcriptomic changes, surface costimulatory molecules, cytokine/chemokine profiles). Such data would better define the DC-intrinsic pathways that promote pathogenic Th17 differentiation.

We thank the reviewer for this very good point regarding the mechanistic role of dendritic cells (DCs) in driving pathogenic Th17 differentiation.
We fully agree that understanding how *P. aeruginosa* clinical isolates reprogram DCs at the molecular level would provide valuable mechanistic insight. Indeed, this precise question—how *P. aeruginosa*-dependent DC reprogramming occurs at the transcriptomic level— is being addressed in a follow-up project led by our group, which is already funded to perform dual RNA-seq on CF-derived DCs infected with different and specific *P. aeruginosa* clinical strains. Given the extensive nature of these datasets and the additional validation required, we intend to include these data in a future separate manuscript.

Nevertheless, to provide additional mechanistic insight into DC activation in response to *P. aeruginosa* infection, beyond the cytokine and chemokine analyses already performed (now **Figure 3c**), we have

included an extensive assessment of surface markers on DCs infected with all clinical *P. aeruginosa* strains included in this study (**new Figure 3b**), as suggested by the Reviewer.
This analysis encompasses a broad panel of surface markers including co-stimulatory molecules (CD40, CD80, CD83, CD86), chemokine and pattern recognition receptors (CCR7, CD206, CD209) and inhibitory molecules (CD172a–SIRPa, CD274–PD-L1, CD366–TIM3), allowing us to directly compare the impact of early versus late clinical *P. aeruginosa* isolates on DC activation.
The results of these new data (new Figure 3b), together with the corresponding correlation analyses between surface marker expression and *P. aeruginosa* virulence traits (new Extended Data Figure 6 and Supplementary Figure 2), strengthen our model and provide a better understanding of the molecular reprogramming of DCs induced by *P. aeruginosa*.
In our view, these analyses provide further mechanistic insight without overextending the scope of the current manuscript, while reserving comprehensive transcriptomic profiling for a separate, forthcoming study.

Dipartimento di Bioscienze
Via Celoria 26 – 20133 Milano, Italy
Tel 02503 15042 - Fax 02503 15044 – direzione.bioscienze@unimi.it

Reviewer #1

Overall I am very impressed with their detailed and very comprehensive response to our comments and the other reviewers' comments.

To recap, our primary concerns were primarily focused on:
- Cohort definitions and patient characterisation
- The definition of chronic infection and impact of polymicrobial infections

The authors have taken great steps to address this including individual patient tables with further granular detail on co-morbidities / disease severity etc. In addition to this they have conducted a correlation analysis to assess impact of possible clinical variables on the distribution of t-cell subsets. For this they showed that for cth17 and pTh17 cells in blood there was higher levels in those with more severe lung disease which they later expand upon with further analysis.

We would, however, welcome some further clarification. From Supplementary Table 3, it appears that there are 6 cases of chronic pulmonary P. aeruginosa infection in the CF lung cohort without co-culture of other pathogens, whereas the Extended Data Figure 2a appears to show more than 6 samples in the Pa+, Sa− group. This raises the possibility that the latter group may include cases with pathogens other than S. aureus. If that is the case, it would be helpful for the authors to state this explicitly and clarify the grouping strategy.

We fully appreciate, as the authors note in their response, that the sample size may limit the feasibility of analysing each pathogen separately. However, if possible, it may still be informative to compare cases with isolated P. aeruginosa infection and those with polymicrobial P. aeruginosa infection, including both S. aureus and other pathogens. Even if exploratory, such an analysis could help support the interpretation that the reported immune differences are associated with P. aeruginosa rather than reflecting polymicrobial infection more broadly.

We would like to first clarify an important point regarding sample inclusion across the different analysis in relation to the Supplementary Tables: we included additional patients during the first revision process to increase cohort representation for IFNγ/IL-17 secretion analysis in Figure 1a, as requested by Reviewer #1 and #2.
These additional samples were analysed ***exclusively*** for IFNγ/IL-17 cytokine production due to limited cell availability and were, therefore, not subjected to downstream immunophenotyping of T-cell subset frequencies, as previously detailed in our response to both Reviewer #1 (Point 2a, first revision round) and to Reviewer #2 (Point 2, first revision round). Accordingly, it is important to note that not all patients reported in Supplementary Tables 3–5 are included in the analysis shown in Extended Data Fig. 2A.

Building on this clarification, the stratification shown in Extended Data Fig. 2a was originally designed, as requested in the first revision round, based on the presence of *P. aeruginosa* and/or *S. aureus* **chronic** lung infection. Accordingly, in the revised manuscript we explicitly clarified the clinical definition used for "chronic infection," which follows the European CF Society Patient Registry (ECFSPR) criteria, defining chronic infection as "the presence of the pathogen in >50% of respiratory samples collected during the 12 months preceding lung transplantation/blood collection". Moreover, we further described that additional bacterial species detected in respiratory cultures were classified as **intermittent co-infections**. As shown in Supplementary Table 3, these pathogens display a highly heterogeneous, patient-specific and sporadic distribution pattern, without consistent recurrence across subjects. This variability, together with the limited cohort size, precludes any biologically meaningful or statistically robust stratification by individual intermittent pathogens, as already noted in our previous response.

Dipartimento di Bioscienze
Via Celoria 26 – 20133 Milano, Italy
Tel 02503 15042 - Fax 02503 15044 - direzione.bioscienze@unimi.it

Nevertheless, to address this new Reviewer's request, we attempted exploratory stratifications incorporating intermittent co-infections **in addition to** chronic *P. aeruginosa* and/or *S. aureus* infection. However, this exploratory approach (see Figure below) results in a highly fragmented grouping structure with several subgroups of extremely limited size (including subgroups with n=1-2 patients), which – in our opinion - substantially limits data interpretability for the readers.

Of note, despite this heterogeneity and sample fragmentation, the enrichment of IFNγ⁺Th17 cell subsets in CF lungs remains consistently associated with chronic *P. aeruginosa* infection, but the resulting visual representation becomes overly complex and difficult to interpret in a biologically meaningful way, particularly when simultaneously considering lung and blood compartments.

A) LUNG

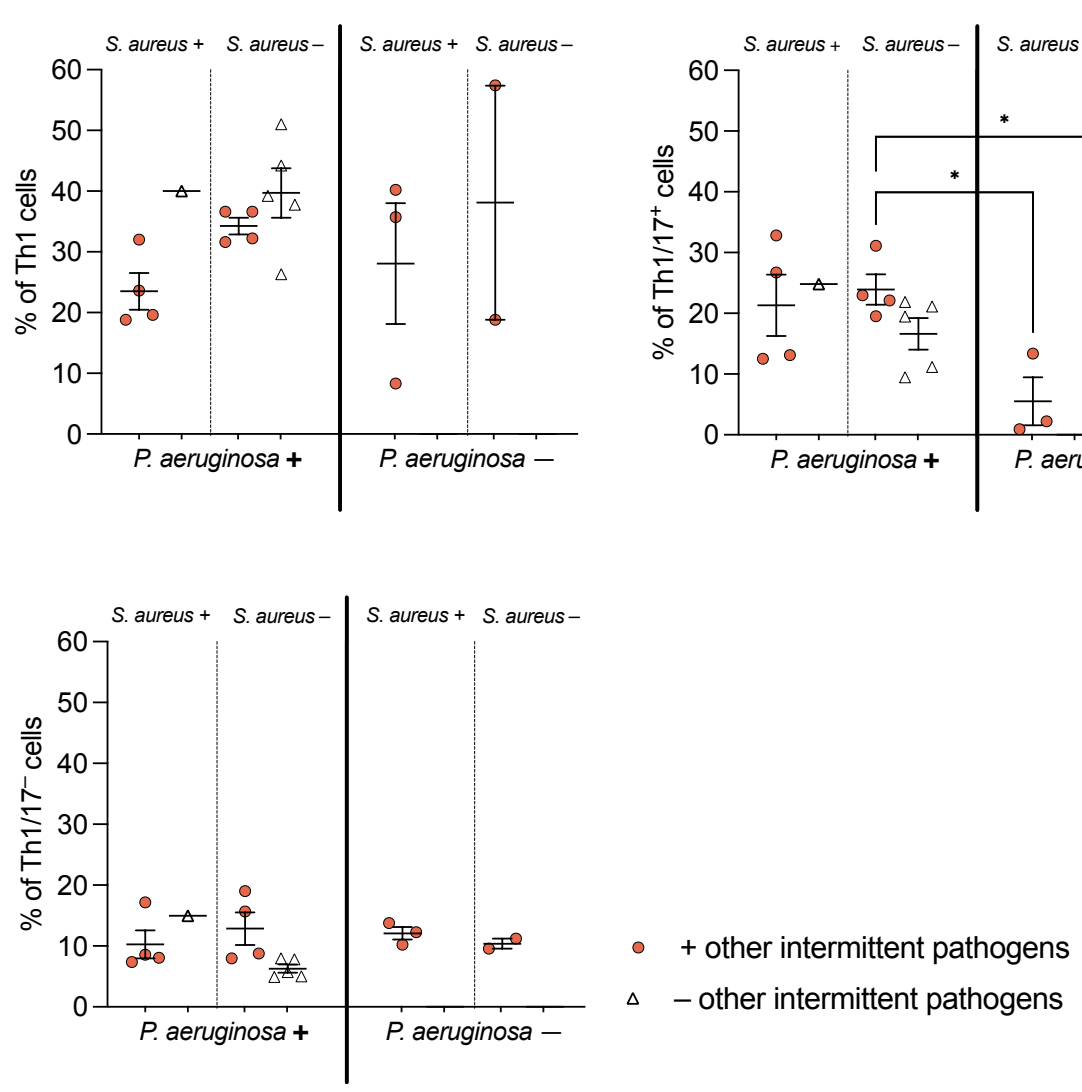

B) BLOOD

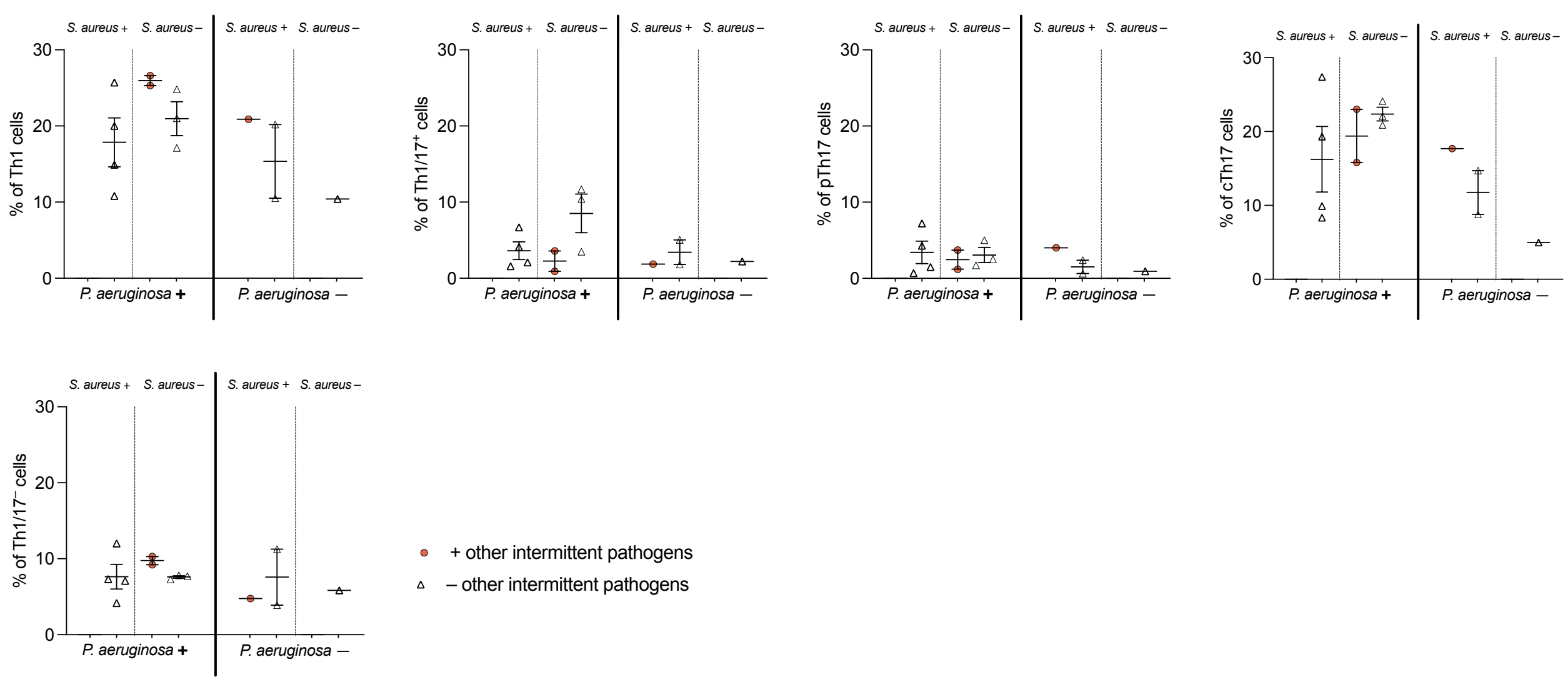

Next, we also explored the alternative stratification suggested by the Reviewer in this second round, by comparing "isolated *P. aeruginosa infection with polymicrobial P. aeruginosa infection, including both chronic S. aureus co-infection and intermittent pathogens"* (see Figure below).
However, this approach introduces additional interpretative limitations, as intermittent infections are temporally transient (often based on one or a few positive cultures within the year preceding sampling) and, therefore, not directly comparable to chronic lung colonization. Indeed, with this stratification that pools together chronic *S. aureus* and intermittent different pathogens, it becomes impossible to establish (neither scientifically nor visually) whether higher frequencies of T-cell subsets are associated with chronic *S. aureus* infection or, instead, reflect the contribution of additional intermittent pathogens co-detected with *P. aeruginosa*. Furthermore, as above, the resulting figure must be split into two separate figures, thereby reducing overall clarity and immediate visual interpretability, particularly when simultaneously considering lung and blood compartments.
Nevertheless, this alternative analysis also confirms that Th1/17$^+$ cell frequencies remain significantly higher in *P. aeruginosa*-positive patients compared with *P. aeruginosa*-negative subjects, irrespective of the presence of polymicrobial intermittent infections. However, this representation does not allow discrimination between the relative contribution of chronic *S. aureus* infection and that of additional intermittent pathogens.

A) LUNG

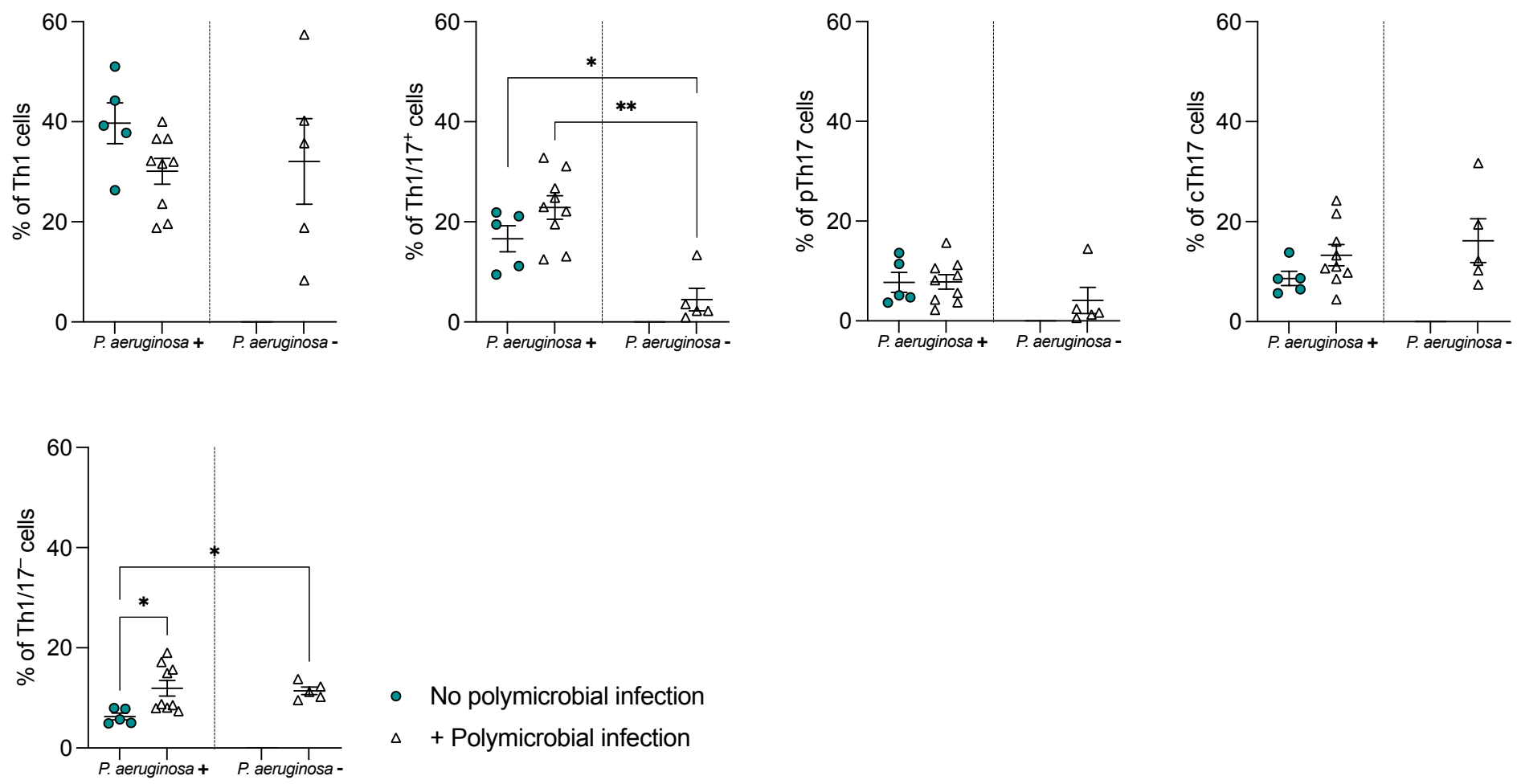

B) BLOOD

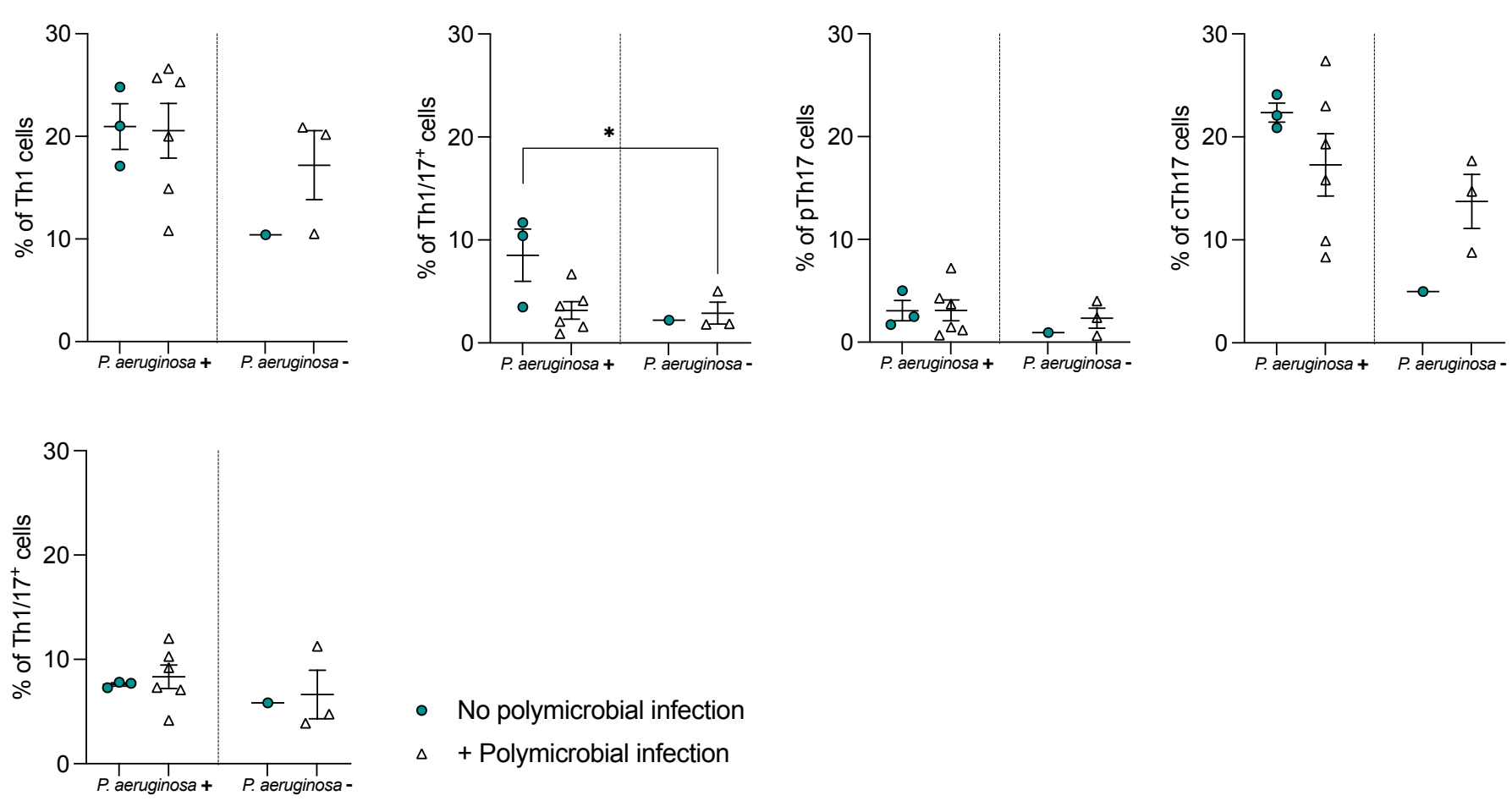

Dipartimento di Bioscienze
Via Celoria 26 – 20133 Milano, Italy
Tel 02503 15042 - Fax 02503 15044 – direzione.bioscienze@unimi.it

For all these reasons, we are convinced that the most informative and biologically coherent representation is to stratify CF patients based on **chronic** infection status (the dominant and clinically validated determinant of airway microbiology in CF), while simultaneously highlighting the presence or absence of intermittent polymicrobial infection **within** these groups.
Accordingly, we have now revised the Extended Data Fig. 2A, by stratifying CF patients with *P. aeruginosa* and/or *S. aureus* chronic infection according to the presence OR absence of intermittent polymicrobial infections (see figure below). Specifically, patients without intermittent co-infections (**empty circles**) are distinguished from those presenting episodic polymicrobial colonization (**full circles**), allowing the reader to visually appreciate the distribution of T-cell subsets across clinically relevant chronic AND intermittent infection categories.

This revised representation offers three key advantages:
1) it preserves clinically meaningful stratification based on chronic airway infection status (consistent with the cohort classification strategy requested during the first round of revision);
2) it explicitly integrates the presence of intermittent polymicrobial events without over-fragmenting the datasets;
3) it enables a clear visual comparison of T-cell subset frequencies across both lung and blood compartments in a single, interpretable figure.

Importantly, this updated analysis continues to support the central conclusion of the study, namely that chronic *P. aeruginosa* infection is the dominant factor associated with increased frequencies of pathogenic IFNγ^{+} Th17 cell subsets in CF lungs.

We hope that this revised analysis and the accompanying clarification fully address the Reviewer's concern and improve the clarity of cohort stratification in the manuscript.

NEW Extended Data Fig. 2 A

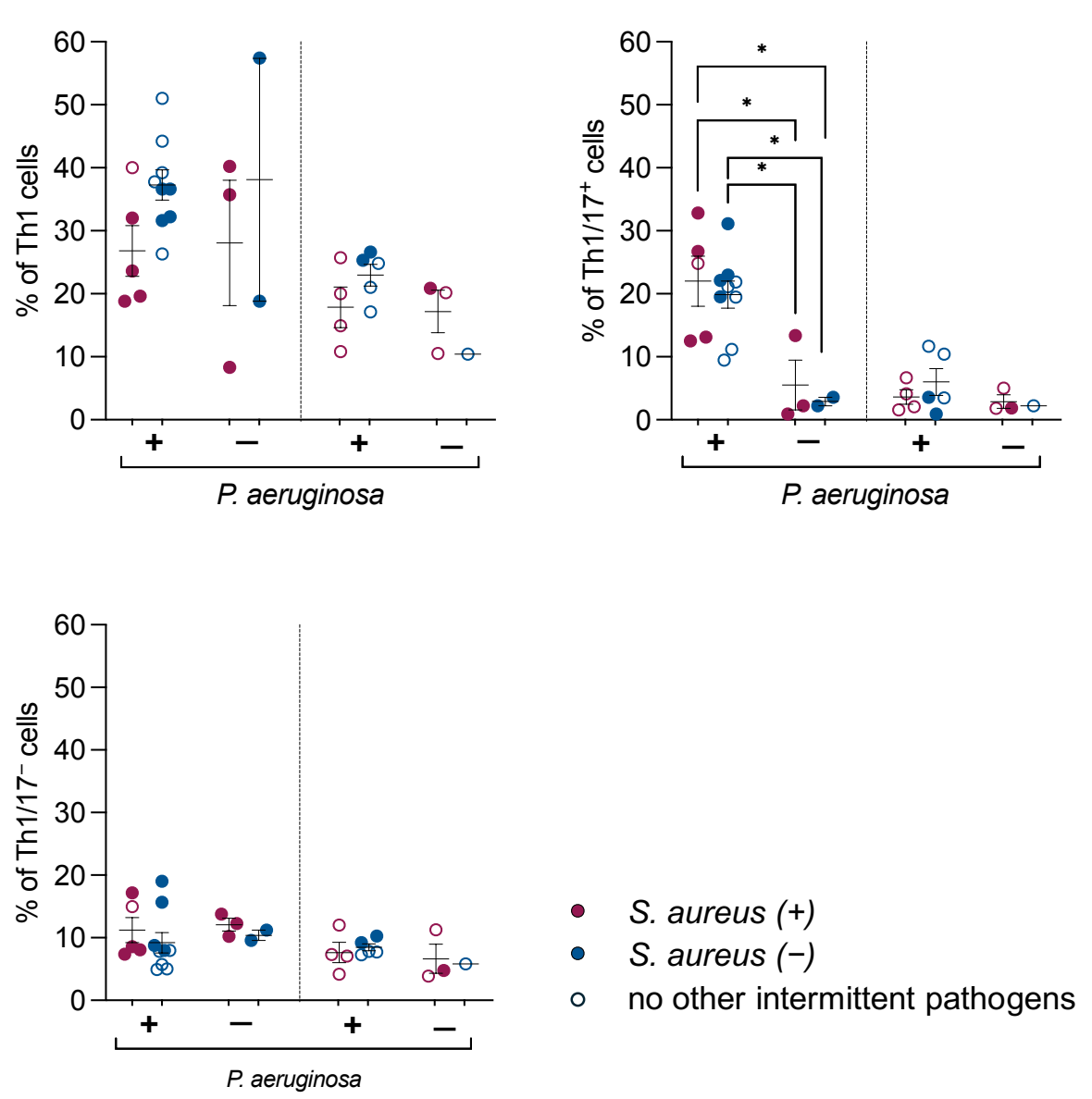

Minor:
The authors have addressed the comments made with regards cohort definitions and the application of the stricter exclusion criterion in great detail and have partly incorporated this into the manuscript [line 442-445]. It would however perhaps be beneficial to the reader if the author included their justification that a stricter criterion was used to allow for an "unbiased assessment of peripheral immune cell frequencies and their comparison with healthy donors".

We revised the sentence at lines 442–445 to explicitly clarify the rationale underlying the stricter exclusion criteria applied to peripheral blood analyses. The sentence now reads:
"A stricter exclusion criterion was specifically applied to the peripheral blood cohort to minimize potential confounding effects from concomitant inflammatory or infectious conditions and enable an unbiased comparison of circulating immune cell frequencies between CF subjects and HD."

We thank the authors who have diligently provided detailed individual patient characteristics where possible in order to provide greater cohort transparency. This information is vital for the reader to understand the cohorts better. In addition to the above, for table 2 it would also be useful for the authors to include in the table legend that for colonisation results reflect potentially > 1 cultured pathogen per patient.

We have now added the following statement to the legend of Table 2 to improve clarity:
"Lung colonization may include more than one pathogen per patient. Detailed microbiological profiles for individual patients are provided in Supplementary Tables 3–5."

Reviewer #2
The authors have addressed my comments

Reviewer #3
The authors have fully addressed my concerns

We are grateful to all the Reviewers for their constructive feedback and positive evaluation of the revised manuscript. We sincerely thank Reviewer #2 and Reviewer #3 for acknowledging the thoroughness of our revisions and for their supportive assessment of our work.

Dipartimento di Bioscienze
Via Celoria 26 – 20133 Milano, Italy
Tel 02503 15042 - Fax 02503 15044 – direzione.bioscienze@unimi.it
